# Repurposing Diels–Alder Chemistry to Access Low-Strain Monomers for Chemically Recyclable ROMP Polymers

**DOI:** 10.1021/acsmacrolett.6c00457

**Published:** 2026-09-23

**Authors:** Tarek Ibrahim, Md Aminur Rahman, Monica Kaczynski, Coogan Cardella, Hao Sun

**Affiliations:** Department of Chemistry and Chemical and Biomedical Engineering, Tagliatela College of Engineering, 8518 University of New Haven, West Haven, Connecticut 06516, United States

## Abstract

Diels–Alder reaction has been widely employed to access highly strained cyclic olefin monomers for efficient ring-opening metathesis polymerization (ROMP). However, the high ring strain energies (RSEs > 15 kcal/mol) of these traditional Diels–Alder monomers, while promoting efficient polymerization, compromise the depolymerizability of the resulting polymers, thereby limiting their chemical recyclability. Herein, we report a class of low-strain Diels–Alder monomers based on *trans*-succinimide-fused cyclohexenes (*t*SICHs), synthesized from readily available feedstocks, fumaric acid and 3-sulfolene. The relatively low RSEs of the *t*SICH monomers (6.1–6.2 kcal/mol) enable both efficient ROMP and ring-closing metathesis depolymerization (RCMD) through concentration-controlled metathesis equilibrium. All *t*SICH monomers achieved high conversions (>91%) during ROMP at an initial monomer concentration of 5 M, yielding polymers with high molecular weights (>100 kDa). Moreover, near-quantitative monomer regeneration (>95%) was achieved via RCMD at room temperature with an olefin concentration of 0.2 M. Critically, the resulting polymers exhibit highly tunable thermomechanical properties, affording a broad range of glass transition temperatures (30–78 °C) and tensile strains at break (3–550%). Overall, this work provides a new monomer design strategy that unlocks access to chemically recyclable polymers from Diels–Alder-derived ROMP monomers.

## Graphical abstract

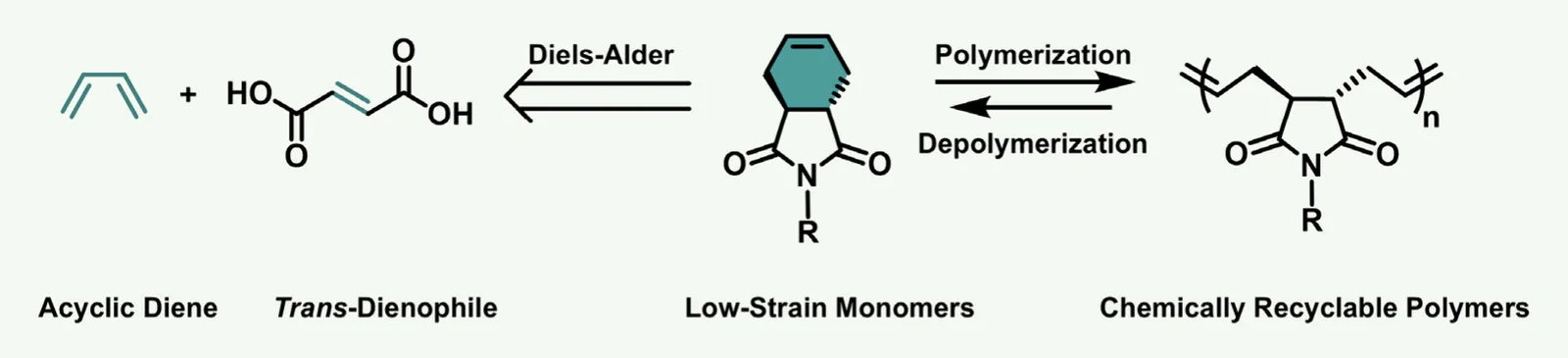

Ring-opening metathesis polymerization (ROMP) is a powerful and versatile polymerization technique that has enabled the synthesis of structurally diverse functional polymers for a wide range of industrial applications.^1^ In a typical enthalpy-driven ROMP process, the release of ring strain energy (RSE) from cyclic olefin monomers upon ring opening provides the enthalpic driving force for polymerization. Accordingly, highly strained cyclic olefin monomers are generally preferred for efficient ROMP.^2,3^ Diels–Alder chemistry represents one of the most common synthetic approaches for constructing high-strain ROMP monomers, in which cyclic dienes, such as cyclopentadiene and furan, react with dienophiles to generate strained bridged bicyclic olefins (Figure 1a). Leveraging the broad scope of cyclic dienes and dienophiles, numerous Diels–Alder-based ROMP monomers have been developed, including norbornene derivatives,^4–7^ oxanorbornene derivatives,^8–10^ thianorbornene,^11^ Vince lactacm,^12^ azanorbornenes,^13^ 7-oxa-2,3-diazanorbornenes,^14^ pyridinonorbornenes,^15^ benzonorbornadiene,^16^ and dicyclopentadiene.^17,18^ Among these monomers, norbornene and dicyclopentadiene have been widely employed for producing high-performance industrial polymers and thermosets.^19^

**Figure 1:**
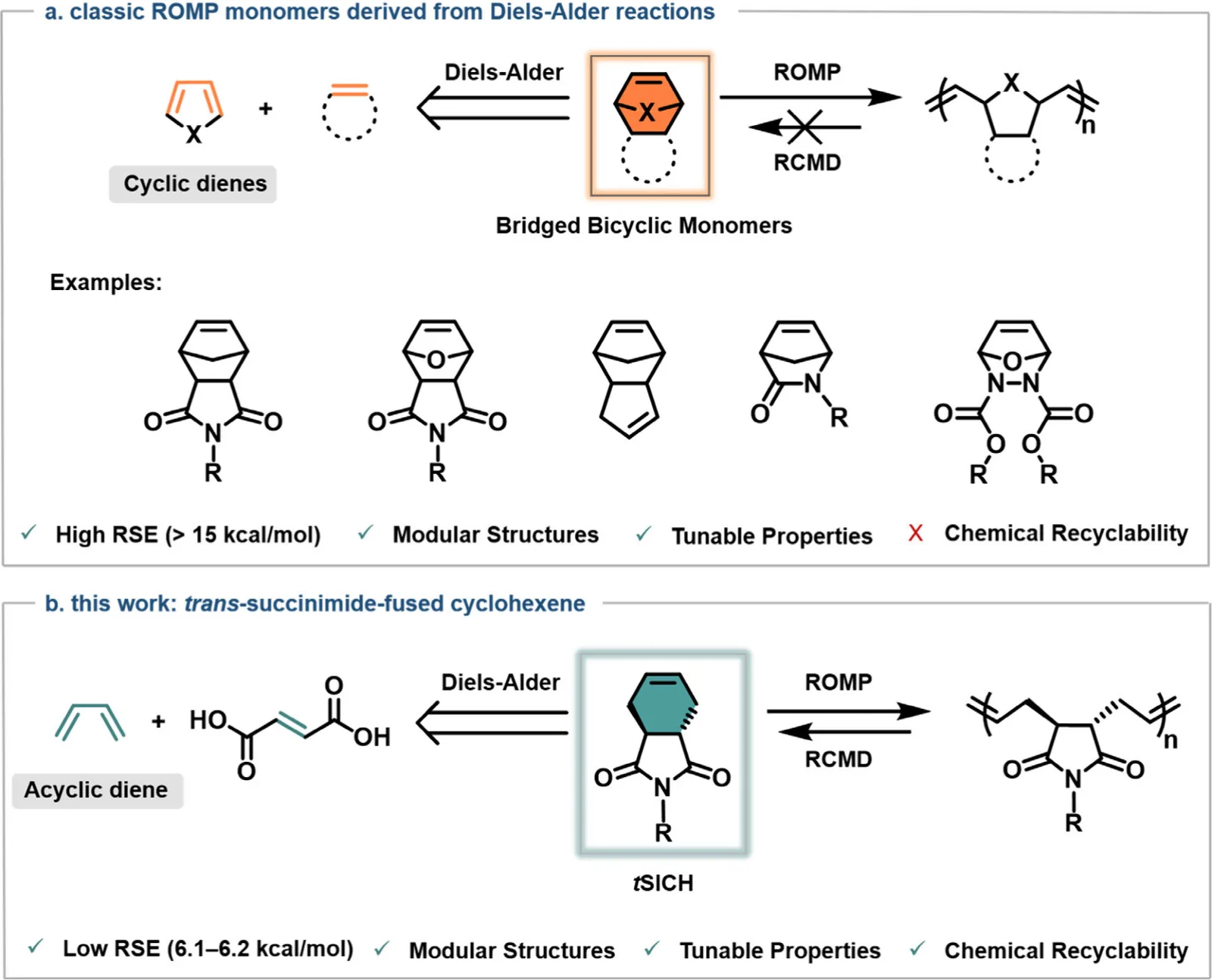
Diels–Alder-derived cyclic olefin monomers for ring-opening metathesis polymerization (ROMP). (a) Classic norbornene-based monomers with high ring-strain energies (RSEs) prepared by Diels–Alder reactions. Polymers derived from norbornene derivatives are not chemically depolymerizable. (b) *trans*-Succinimide-fused cyclohexene monomers (*t*SICH) developed in this work. The relatively low RSEs of *t*SICH enable chemical recycling of the resulting polymers through ROMP and ring-closing metathesis depolymerization (RCMD).

Chemical recycling to monomers is a promising approach toward a circular plastics economy.^20,21^ Despite the advantages of highly strained monomers in ROMP, the resulting polymers are challenging to chemically recycle back to their original monomers because depolymerization is thermodynamically unfavorable for these systems.^22^ For example, the high RSE of norbornene leads to an exceptionally high ceiling temperature for polynorbornene (>1000 K), rendering monomer recovery under practical conditions infeasible.^23^ To enhance the chemical recyclability of ROMP-derived polymers toward a circular plastics economy, recent studies have focused on developing low-strain cyclic olefin monomers with balanced polymerization thermodynamics that enable both ROMP and ring-closing metathesis depolymerization (RCMD) under mild conditions.^24,25^ To date, three general strategies have emerged for accessing such low-strain ROMP monomers. The first strategy involves the direct use of commercially available cyclic olefins with low RSEs, including cyclopentene derivatives,^26–31^ cycloheptene,^32,33^ 2-cycloheptenone,^34^ and 2,3-dihydrofuran,^35^ to generate chemically recyclable polymers. In the second strategy, commercially available cyclic olefins, such as 1,5-cyclooctadiene,^36–40^ 3-cyclohexene-1-carboxylic acid,^41^ and 1,4-cyclohexadiene,^42,43^ were structurally modified to tune the RSEs of the resulting monomers into the desired range for depolymerizable polymers. The third strategy employs ring-closing metathesis reactions to synthesize macrocyclic and medium-sized monomers with low RSEs.^44–48^

Among various low-strain ROMP monomers, six-membered cyclohexene-based monomers remain a relatively underexplored platform. While a few cyclohexene-derived monomers have recently been reported for the synthesis of depolymerizable polyhexenamers,^41–43^ the development of a generalizable and modular synthetic approach to this emerging class of polymers would be highly desirable. Given that the Diels–Alder reaction provides an atom-economical route to cyclohexene frameworks, we envisioned that this versatile chemistry could enable the modular design of low-strain cyclohexene monomers. Herein, we introduce a synthetic strategy that leverages Diels–Alder chemistry to access a new class of low-strain ROMP monomers (Figure 1b). Specifically, a Diels–Alder reaction between fumaric acid and 3-sulfolene (a precursor to 1,3-butadiene), followed by ring closure and imidization, affords a class of *trans*-succinimide-fused cyclohexene monomers (*t*SICHs) with low RSEs of 6.1–6.2 kcal/mol. Despite their relatively low strain, all *t*SICH monomers undergo efficient ROMP, achieving high monomer conversions (>91%) and high molecular weights (>100 kDa) at an initial monomer concentration of 5 M. Furthermore, RCMD of the resulting polymers at room temperature and an olefin concentration of 0.2 M leads to near-quantitative monomer recovery (>95%). The modular structures of *t*SICH monomers further enable systematic tuning of the thermomechanical properties of the resulting polymers, demonstrating their potential as sustainable polymer platforms with tailored performance.

To access Diels–Alder-derived ROMP monomers with lower ring-strain energies than norbornene derivatives, we selected the acyclic diene 1,3-butadiene as the diene component. In contrast to cyclic dienes such as cyclopentadiene or furan, this design would generate a cyclohexene framework rather than a bridged bicyclic structure, thereby substantially reducing the ring strain (Figure 1b). Nevertheless, the RSE of cyclohexene (2.5 kcal/mol) and its derivatives is known to be insufficient to drive ROMP.^49,50^ Recent studies have shown that fusion with a *trans*-cyclic carbonate increases the ring strain of cyclohexene derivatives by inducing ring distortion, thus rendering these monomers active toward ROMP.^42^ Inspired by this strategy, we envisioned that a *trans*-dienophile such as fumaric acid could be coupled with 1,3-butadiene to generate *trans*-cyclohexene structures. Subsequent ring closure through anhydride formation and imidization would afford a class of *trans*-succinimide-fused cyclohexene monomers featuring distorted succinimide ring and RSEs suitable for ROMP (Figure 1b).

To verify our hypothesis, we conducted computational analyses of *trans*-succinimide-fused cyclohexene and compared its ring-strain energy with those of exo-norbornene imide (exo-NBI) and *cis*-succinimide-fused cyclohexene (*c*SICH) (Figure 2 and Figures S42–S44). As expected, exo-NBI exhibits the highest RSE (15.1 kcal/mol) among the three monomers because of its significant angle strain (Figures 2a and S42). Compared to exo-NBI, the RSEs of both *t*SICH (5.9 kcal/mol) and *c*SICH (1.7 kcal/mol) are markedly lower, consistent with the reduced strain associated with the cyclohexene framework relative to the bridged bicyclic norbornene structure (Figures S43 and S44). To elucidate the origin of the RSE difference between *t*SICH and *c*SICH, we further analyzed their Newman projections along the C2–C3 bond. Notably, the carbonyl–C2–C3–carbonyl dihedral angle in *t*SICH is 29.03°, indicating distortion of the *trans*-fused succinimide ring, which is otherwise expected to adopt a planar conformation (Figure 2b). Upon ring opening, this dihedral angle decreases to 9.56°, indicating substantial release of the ring distortion and the associated strain (Table S5). In contrast, *c*SICH exhibits no detectable distortion of the succinimide ring, as evidenced by a carbonyl–C2–C3–carbonyl dihedral angle of 0° (Figure 2c). Moreover, ring opening of *c*SICH generates a structure with pronounced steric repulsion between the two alkyl chains, thereby destabilizing the ring-opened product and consequently lowering the calculated RSE of *c*SICH.

**Figure 2:**
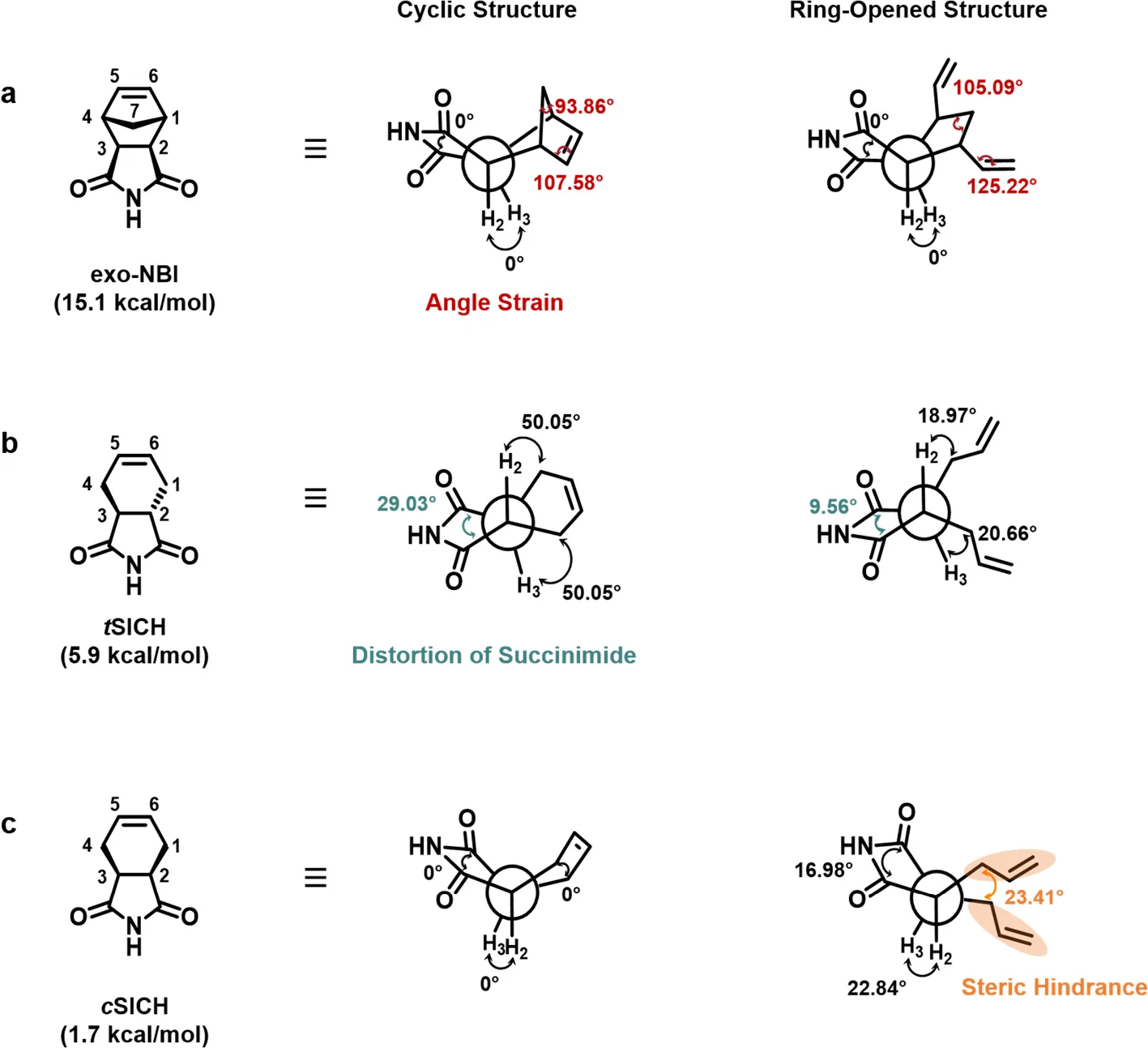
Conformational analysis of monomers and their ring-opened structures. (a–c) Structures and corresponding Newman projections along the C2–C3 bond for exo-norbornene imide (exo-NBI) (a), *t*SICH (b), and *c*SICH (c), along with their ethenolysis ring-opened products. Geometries and energies were optimized using density functional theory at the B3LYP/6–31G­(d) level.

The effect of *N*-substitution on the RSEs of *t*SICH monomers (M1–M4) was further examined. Computational analyses revealed that the *N*-substituents had minimal influence on the RSE values, which remained within a narrow range of 6.1–6.2 kcal/mol (Figures S45–S48). This finding highlights the structural modularity of the *t*SICH monomer platform while preserving monomer reactivity toward ROMP and RCMD (*vide infra*). The synthesis of *t*SICH monomers was accomplished in three straightforward steps (Figure 3a). A Diels–Alder reaction between fumaric acid and 3-sulfolene first afforded the *trans*-diacid intermediate, which was converted into *trans*-succinic anhydride-fused cyclohexene (*t*SACH) in 75% overall yield (Figures S1–S4). Subsequent imidization of *t*SACH with various primary amines gave rise to a series of *t*SICH monomers (M1–M5) (Figures S5–S15).

**Figure 3:**
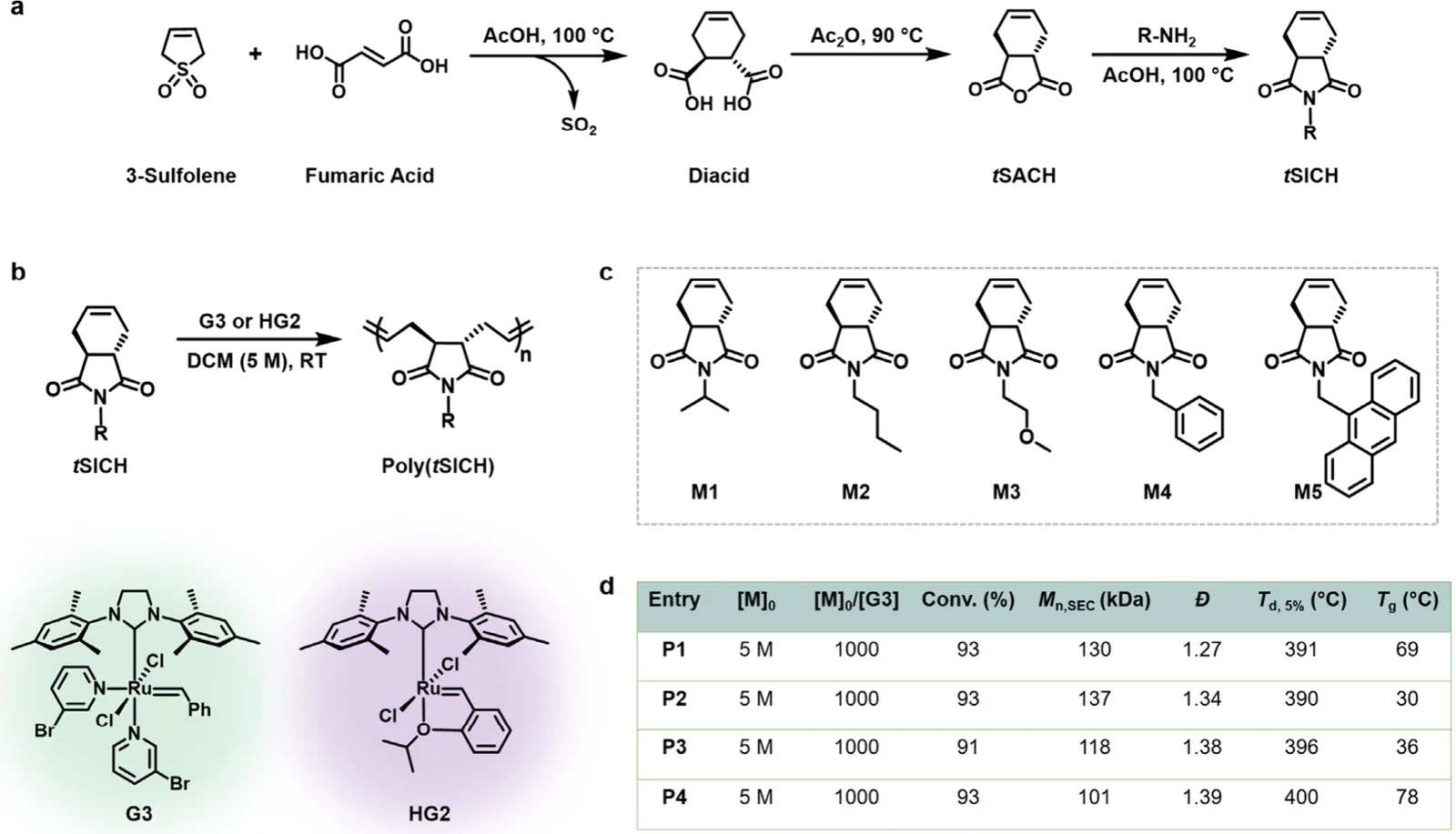
Synthesis of *t*SICH monomers and their corresponding polymers. (a) Three-step synthesis of *t*SICH monomers. (b) Synthesis of *t*SICH-based polymers via ring-opening metathesis polymerization in dichloromethane (DCM). Polymers P1–P4 were synthesized using the Grubbs third-generation catalyst (G3), whereas the copolymer P3-*r*-P5 was prepared using the Hoveyda–Grubbs second-generation catalyst (HG2). (c) Scope of the *t*SICH monomers investigated in this study. (d) Polymerization results and thermal properties of polymers P1–P4.

Because *t*SICH monomers possess relatively low RSEs, we reasoned that increasing the monomer concentration would favor higher monomer conversions. Accordingly, all polymerizations were conducted at an initial monomer concentration of 5 M and a monomer-to-initiator ratio of 1000:1 (Figure 3b). The polymerization kinetics study of the representative monomer M4 indicated that the polymerization reached equilibrium within 120 min (Figure S16). Notably, monomers M1–M4 reached conversions exceeding 91% in the presence of Grubbs third-generation catalyst (G3), affording polymers with high molecular weights (100–140 kDa) and relatively narrow molecular weight distributions (*Đ* < 1.4) (Figure 3c,d and Figures S17–S25). In contrast, the limited solubility of M5 at high monomer concentrations precluded its homopolymerization. Copolymerization of M5 and M3 in the presence of G3 resulted in negligible conversion. However, copolymerization of M5 (5 mol %) with M3 (95 mol %) using the Hoveyda–Grubbs second-generation catalyst (HG2) afforded the random copolymer P3-*r*-P5 with a molecular weight comparable to those of P1–P4 (Figures S26 and S27).

The thermal properties of the resulting polymers were further investigated by thermogravimetric analysis (TGA) and differential scanning calorimetry (DSC) (Figure 3d). TGA revealed excellent thermal stability for all polymers, with decomposition temperatures exceeding 390 °C (Figure 4a and Figure S28). DSC showed that the polymers exhibit glass transition temperatures (*T*_g_) spanning a wide range from 30 to 78 °C (Figure 4b). Notably, P1 and P4 displayed higher *T*_g_ values than P2 and P3, which can be attributed to their more rigid side chains. Similarly, increasing the M5 content in the random copolymer P3-*r*-P5 increased the *T*_g_, consistent with the incorporation of rigid anthracene side groups (Figure S29).

**Figure 4:**
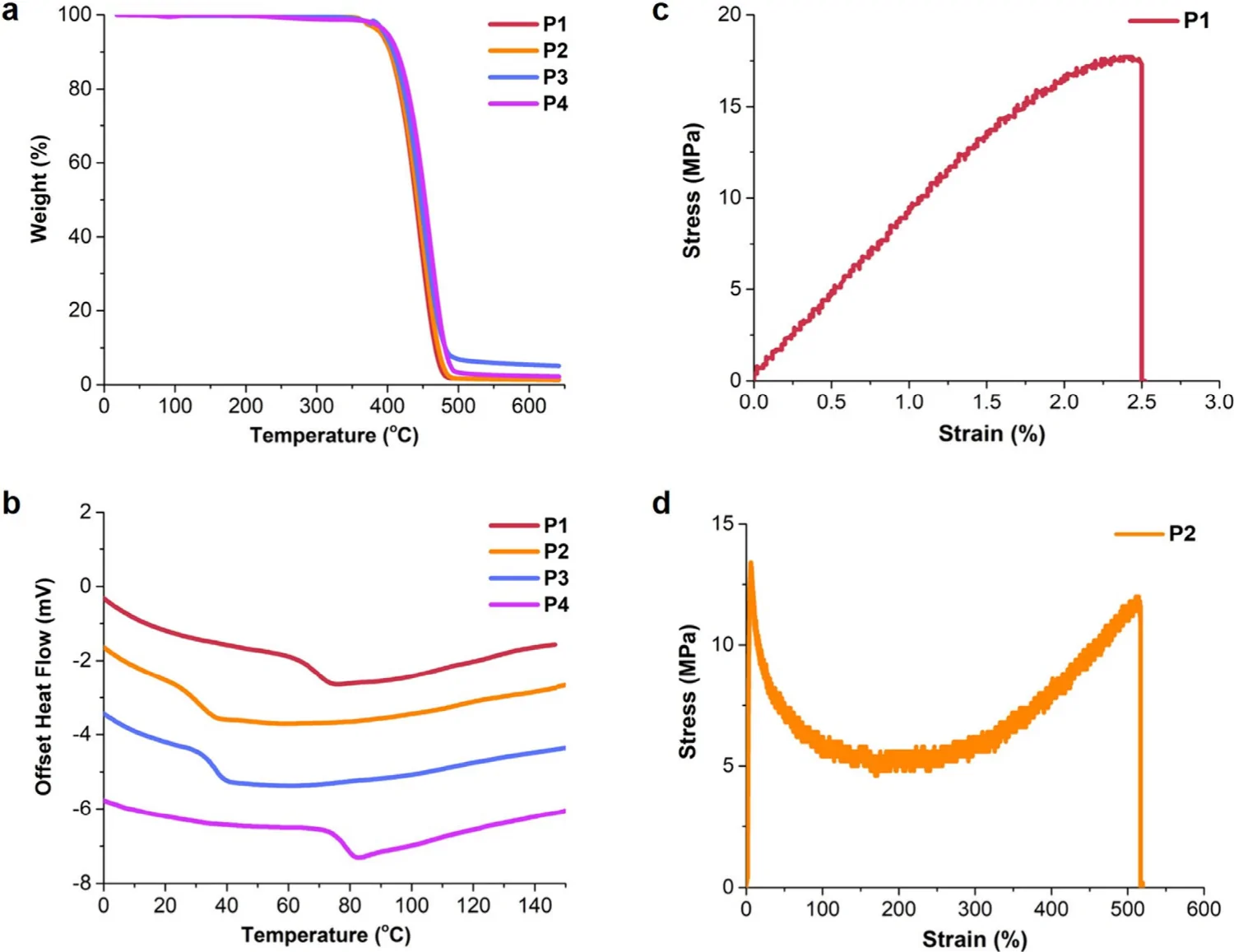
Thermomechanical properties of *t*SICH-based polymers. (a) Thermogravimetric analysis (TGA) curves of P1–P4. (b) Differential scanning calorimetry (DSC) thermograms of P1–P4. (c, d) Representative stress–strain curves of P1 and P2 measured at room temperature with a crosshead speed of 5 mm/min.

The highly tunable glass transition temperatures of the polymers developed in this study are expected to enable diverse mechanical properties for various applications. To evaluate their potential applications, the mechanical properties of representative polymers P1 and P2 with dramatically different *T*_g_ values were assessed by tensile testing (Figure 4c,d and Figures S30 and S31). Consistent with its relatively higher *T*_g_ (69 °C), P1 showed a strong and stiff mechanical response, with a Young’s modulus of 0.80 ± 0.11 GPa, an ultimate tensile strength of 17.6 ± 0.1 MPa, and a maximum strain of 3.3 ± 0.4% (Figure S30). In contrast, P2, with a *T*_g_ of 30 °C close to the testing temperature, displayed a highly ductile behavior, exhibiting a Young’s modulus of 0.67 ± 0.06 GPa, an ultimate tensile strength of 10.3 ± 2.0 MPa, and a maximum strain of 468 ± 64% (Figure S31). These results demonstrate that the tunable side-chain structures of *t*SICH monomers offer an effective strategy for tailoring the thermal and mechanical properties of the resulting polymers.

To shed light on the chemical recyclability of *t*SICH-derived polymers, we determined the ceiling temperatures (*T*_c_) of representative polymers P1 and P4 by analyzing their thermodynamic parameters of polymerization (Figures S32 and S33 and Tables S1 and S2). ^1^H NMR spectroscopy was used to determine the equilibrium monomer concentration ([M]_eq_) during solution polymerization at different temperatures (Tables S1 and S2). Using van’t Hoff analysis, the enthalpy changes (Δ*H*_p_) and entropy changes (Δ*S*_p_^o^) of polymerization under the standard state conditions were obtained from the slope and intercept of the corresponding plots, respectively (Figures S32 and S33). Notably, the experimentally observed Δ*H*_p_ values are smaller than the corresponding computed RSEs. This disparity between RSEs and Δ*H*_p_ is commonly observed in ROMP and can be attributed, in part, to the formation of cyclic oligomers,^36^ and the energy absorbed by polymer conformations.^51^

The standard-state ceiling temperatures, *T*_c_ (1 M), were subsequently calculated from the ratio of Δ*H*_p_ to Δ*S*_p_^o^, giving values of 197 °C for P1 and 161 °C for P4. While the *T*_c_ (1 M) values of P1 and P4 are substantially lower than that of polynorbornene, they remain above the practical operating temperatures of Grubbs catalysts for ring-closing metathesis depolymerization. Because ceiling temperatures are concentration-dependent,^52,53^ we further calculated the *T*_c_ values under relatively dilute conditions (0.2 M) (Supporting Information, Section 4.10). Under these conditions, the *T*_c_ values significantly decreased to – 22 °C for P1 and – 19 °C for P4 (Figures S32 and S33). Therefore, dilute conditions (<0.2 M) at ambient temperature were predicted to render depolymerization thermodynamically favorable.

Guided by the polymerization thermodynamic results, we further screened the depolymerization conditions for P4 by varying the temperature (20 and 35 °C), the loading of Grubbs second-generation catalyst (G2, 1, and 2 mol %), and polymer concentration (0.02–0.2 M of olefins) (Table S3). With a G2 loading of 2 mol %, near-quantitative depolymerization was achieved under all investigated conditions (Figure S34), in good agreement with the thermodynamic prediction based on the calculated ceiling temperature of P4 at 0.2 M (*vide supra*). Depolymerization kinetics revealed over 90% conversion within 3 h, with quantitative monomer regeneration achieved after 12 h using 0.2 M olefins at room temperature (Figure S35). Considering the reduced solvent consumption and energy input, a concentration of 0.2 M at 20 °C was selected as the preferred condition for subsequent depolymerization studies of other polymers (P1–P3 and P3-*r*-P5). Under these conditions, all polymers achieved quantitative monomer regeneration, as confirmed by both NMR and size-exclusion chromatography (SEC) analyses (Figure 5, Figures S36–S39, and Table S4).

**Figure 5:**
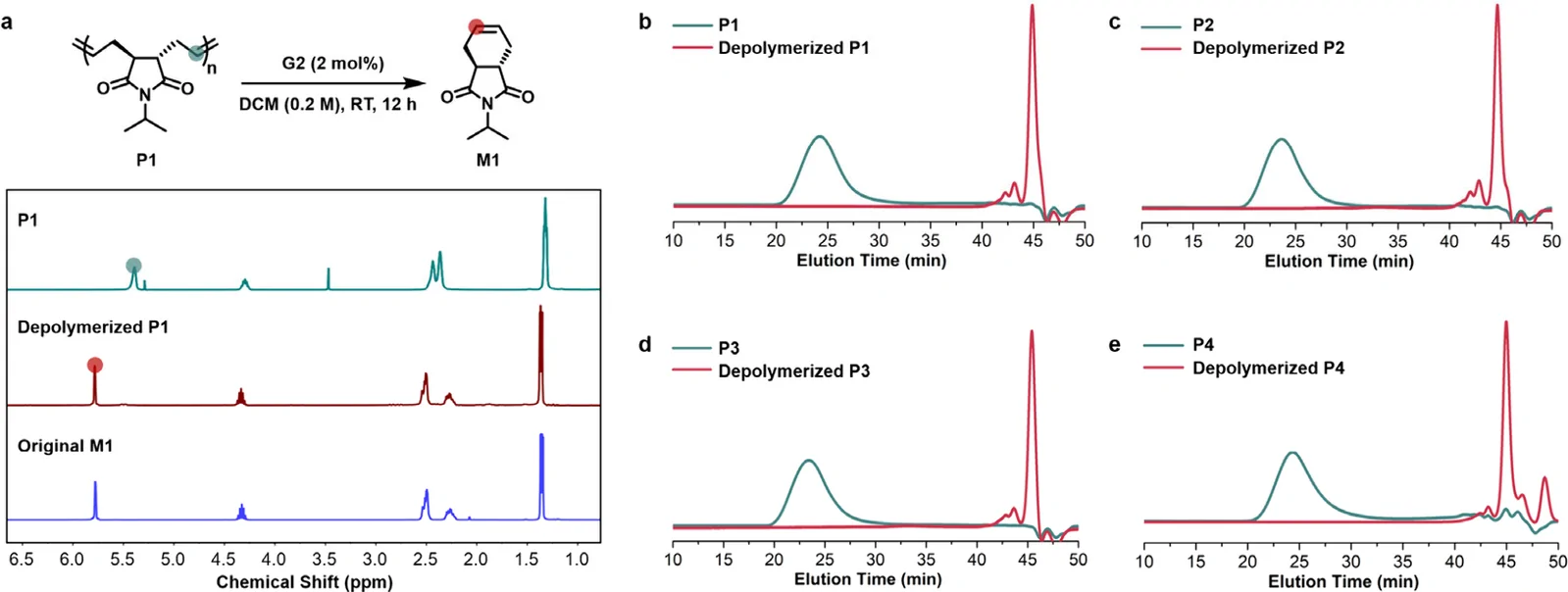
Depolymerization studies of *t*SICH-based polymers. (a) Schematic illustration of the depolymerization of P1. Quantitative monomer recovery was demonstrated by ^1^H NMR spectra of P1 before (green) and after (red) 12 h of stirring in DCM at 20 °C in the presence of Grubbs second-generation catalyst (G2). The ^1^H NMR spectrum of the original M1 (blue) is shown for reference. (b–e) Size exclusion chromatography (SEC) traces for P1 (b), P2 (c), P3 (d), and P4 (e) before (in green) and after (in red) depolymerization. In all cases, the SEC traces of polymers disappeared completely, accompanied by the appearance of monomer peaks, confirming quantitative depolymerization.

To expand the architectural scope of depolymerizable polymers in the *t*SICH system, we further prepared a cross-linked polymer network via UV-induced [4 + 4] dimerization of anthracene pendant groups of P3-*r*-P5 under 365 nm irradiation (Figure 6a). Solution-based cross-linking proceeded rapidly, reaching completion within 6 min, as confirmed by the disappearance of characteristic anthracene absorption bands in the UV–vis spectra (Figure 6b). In contrast, solid-state cross-linking, in which the polymer film was prepared prior to irradiation, proceeded more slowly and reached a lower degree of dimerization (65%) because of restricted chain mobility (Figure S40). Furthermore, heating the cross-linked film at 110 °C enabled regeneration of the linear polymer through dissociation of the anthracene dimers, as evidenced by the recovery of the anthracene absorption bands in the UV–vis spectra (Figure S41).

**Figure 6:**
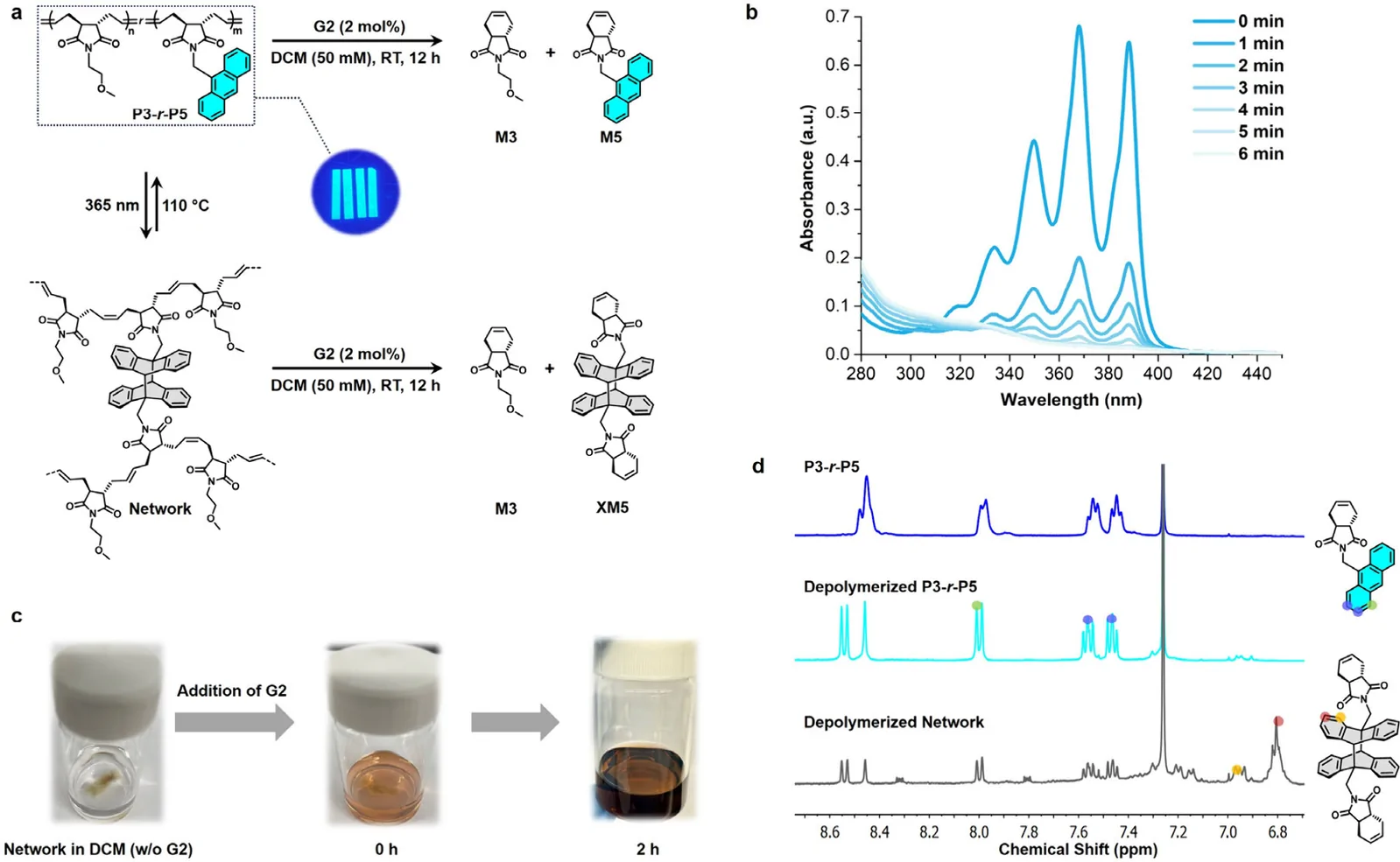
Depolymerization study of *t*SICH-based polymer network. (a) Schematic illustration of the UV-induced cross-linking, thermally induced de-cross-linking, and depolymerization processes of P3-*r*-P5 and its cross-linked network. An inset shows fluorescent P3-*r*-P5 films under 365 nm irradiation. (b) UV–vis spectra of a P3-*r*-P5 solution as a function of irradiation time under 365 nm light. (c) Photographs of the cross-linked P3-*r*-P5 film before and after depolymerization. The network was completely dissolved after 2 h of G2 addition. (d) Partial ^1^H NMR spectra of P3-*r*-P5 (blue), depolymerized P3-*r*-P5 (cyan), and depolymerization products of the cross-linked P3-*r*-P5 film (gray).

The depolymerizability of the cross-linked polymer film prepared via solid-state UV cross-linking was demonstrated under the same RCMD conditions used for the corresponding linear polymers. Upon addition of G2 to the polymer network immersed in DCM, rapid depolymerization occurred, resulting in the formation of a homogeneous solution after 2 h (Figure 6c). NMR analysis further verified the depolymerization of the network by revealing the characteristic signals of the M5 dimer (XM5) (Figure 6d). It is worth noting that a mixture of M5 and XM5 was observed in the depolymerized solution, which can be attributed to incomplete cross-linking during solid-state irradiation. Based on the NMR analysis, the ratio of XM5 to M5 is 57:43, corresponding to a cross-linking degree of 57%, which is in good agreement with the result obtained from the UV–vis spectroscopy (*vide supra*). These results demonstrate that *t*SICH-based polymer networks, even after cross-linking and becoming insoluble in organic solvents, can still undergo efficient depolymerization under mild RCMD conditions. Moreover, the reversible covalent nature of the anthracene dimer-based cross-linkers enables a complementary chemical recycling pathway for cross-linked polymer networks beyond backbone depolymerization (Figure 6a).

In summary, we demonstrate a class of Diels–Alder-derived ROMP monomers based on *trans*-succinimide-fused cyclohexenes with ring strain energies intermediate between highly strained norbornene and unstrained cyclohexene. This optimized strain balance enables efficient polymerization while preserving the ability for chemical depolymerization. By simply controlling the olefin concentrations, both polymerization and depolymerization can be performed with high efficiency (>90%), enabling the chemical recycling of these olefin-based polymers. This monomer design strategy is highly modular and allows for the introduction of various side-chain groups through *N*-substitution, providing access to polymer materials with tunable thermomechanical properties. Given the high atom economy and synthetic efficiency of Diels–Alder chemistry, we envision that repurposing this chemistry for the development of low-strain cyclic olefin monomers will open new opportunities for chemically recyclable polymers toward a circular plastics economy.

## Supplementary Material



## Notes

Supplementary figures and tables, characterization data, ^1^H and ^13^C NMR spectra of monomers and polymers, SEC analysis of polymers, TGA and DSC analyses, tensile testing results, computational studies, and detailed experimental protocols (PDF)
