# Supplementary material for: Repurposing Diels–Alder Chemistry to Access Low-Strain Monomers for Chemically Recyclable ROMP Polymers
Source: ACS Macro Lett. 2026 Sep 23;15(10):1594–601. doi: 10.1021/acsmacrolett.6c00457 (PMC13689908; doi:10.1021/acsmacrolett.6c00457)
Supplement: Supplementary file 1 [file mz6c00457_si_001.pdf]

Supplementary Information for

Repurposing Diels–Alder Chemistry to Access Low-Strain Monomers for Chemically Recyclable ROMP Polymers

Tarek Ibrahim,[#] Md Aminur Rahman,[#] Monica Kaczynski, Coogan Cardella, and Hao Sun*

Department of Chemistry and Chemical & Biomedical Engineering, Tagliatela College of Engineering, University of New Haven, West Haven, Connecticut 06516, USA

Corresponding to: Dr. Hao Sun, Email: hasun@newhaven.edu

[#]These authors contributed equally to this work.

Supplementary Information:

Materials and Instrumentation

Computational Study

Experimental Methods

Figures S1 to S48

Tables S1 to S5

Optimized Geometries of Monomers and Their Ring-Opened Structures from DFT Calculations

References

Table of Contents

1. Materials 3

2. Instrumentation 3

3. Computational Study 4

4. Experimental Methods 5

4.1. Synthesis of Compound 1 5

4.2. Synthesis of Compound 2 5

4.3. Synthesis of M1 6

4.4. Synthesis of M2 6

4.5. Synthesis of M3 7

4.6. Synthesis of M4 8

4.7. Synthesis of M5 8

4.8. General ROMP Procedure 9

4.9. Synthesis of Copolymer P3-*r*-P5 10

4.10. Investigation of Polymerization Thermodynamics 10

4.11. Depolymerization Procedure 11

4.12. Photoinduced Crosslinking of P3-*r*-P5 12

4.13. Thermally Induced Decrosslinking 12

5. Supporting Figures and Tables 13

6. Optimized Geometries of Monomers and Their Ring-Opened Structures from DFT Calculations 47

References 61

1. Materials

Fumaric acid (>99%), 3-sulfolene (98%), acetic acid (AcOH, > 99.7%), acetic anhydride (Ac_2O, >99%), n-butylamine (>99%), isopropylamine (99%), benzylamine (99%), anthracen-9-ylmethanamine (97%), 2-methoxyethylamine (>98%), methanol (99%), ethyl vinyl ether (99%), and triethylamine (TEA) (> 99.5%) were purchased from Fisher Scientific and used without purification. $CDCl_3$ (99.8 atom % D), DMSO-d_6 (99.9 atom % D), Grubbs catalyst 2nd generation (M204), Hoveyda-Grubbs catalyst (M720), and Grubbs catalyst 3rd generation (M300) were purchased from Sigma Aldrich and used without purification.

2. Instrumentation

Nuclear Magnetic Resonance (NMR): ^1H NMR spectra were recorded on a Bruker spectrometer (400 MHz) in deuterated solvents. Chemical shifts are given in ppm downfield from tetramethylsilane (TMS).

Size Exclusion Chromatography (SEC): The molecular weight and polydispersity of synthetic polymers were determined by a size exclusion chromatography (SEC) system (TOSOH EcoSEC HLC-8320) equipped with a set of Phenomenex Phenogel 5μ, 1K-75K, 300 x 7.80 mm in series with a Phenomex Phenogel 5μ, 10K-1000K, 300 x 7.80 mm columns following a guard column and two detectors including a RI detector and a UV detector. The measurements were performed using HPLC-grade THF as the eluent at a flow rate of 0.5 mL/min at 35 °C and a series of polystyrene standards for the calibration of the columns.

Differential Scanning Calorimetry (DSC): Differential scanning calorimetry (DSC) measurements were performed using a Guangdong Newgoer DSC-300C system under a nitrogen gas flow (100 mL/min). Two thermal cycles with heating and cooling rates of 10 °C/min were

performed. The glass transition temperatures were obtained from the second heating scans after removing the thermal history of polymers.

Thermogravimetric Analysis (TGA): Thermogravimetric analysis (TGA) was performed using a TA SDT Q600 system under a nitrogen gas flow (100 mL/min) with a heating rate of 10 °C/min. The temperature range for the analysis extended from ambient temperature to 650 °C.

Tensile Test: Tensile testing of polymers was performed using a Mark-10 F305-IMT system with a 200 N load at room temperature. A crosshead speed of 5 mm/min was used. P1 and P2 films were solvent-cast from a concentrated DCM solution in a covered Petri dish overnight, then dried under vacuum at 40 °C for two days. Rectangular samples (10 mm (L) × 5 mm (W)) were then cut from the polymer film using a razor blade.

UV-Vis Spectroscopy: UV-Vis spectra were obtained on a Shimadzu UV-2600i at 25 °C.

Gas Chromatography-Mass Spectrometry (GC-MS): Gas chromatography-mass spectrometry (GC-MS) analyses were performed on a Shimadzu 2010 series GC system equipped with a Shimadzu QP 2010s mass selective detector, an AOC-20i autosampler, and a Quadrex column (007 MPS5 - 25 m -0.25 mm – I.D. 0.5 µm).

3. Computational Study

The ring strain energies of cyclic olefin monomers were calculated using density functional theory (DFT) with Spartan software. To examine the various conformations available in both the monomers and their ring-opened products, an exhaustive conformer search was performed at the AM1 level of theory. The geometries and energies of the resulting conformers were then optimized at the B3LYP/6-31G(d) level of theory in the gas phase. The enthalpy changes (ΔH) were estimated as the enthalpy difference between the ring-opened products and the total enthalpy of the isolated reactants (monomer + ethylene), using their lowest-energy conformers.

4. Experimental Methods

4.1. Synthesis of Compound 1

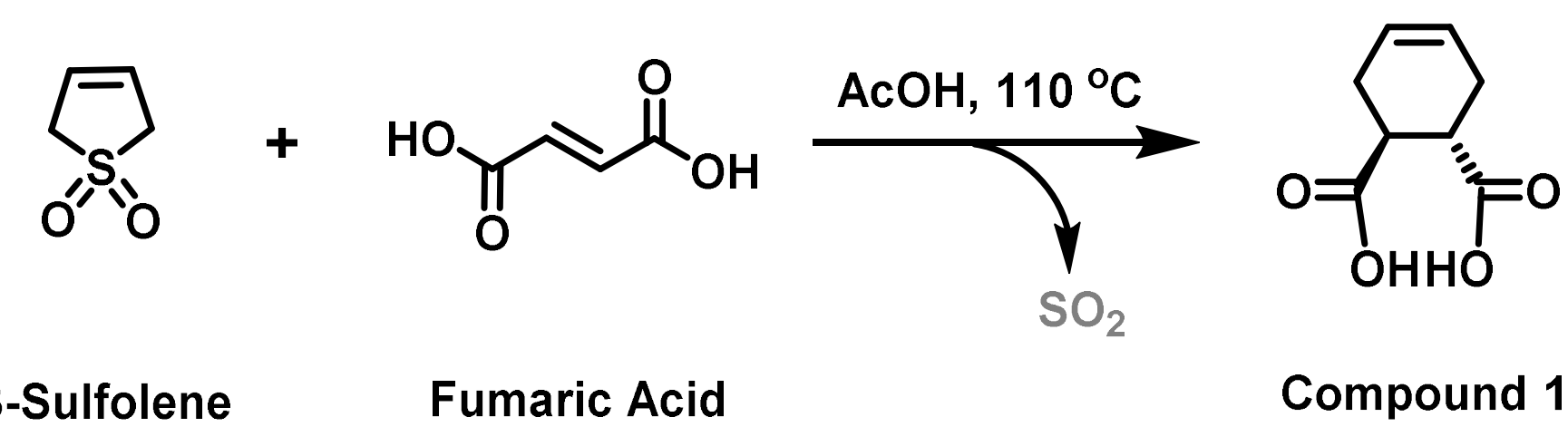

The synthesis of compound 1 was adapted from a previously reported literature procedure.[1] Fumaric acid (5.0 g, 0.043 mol) and 3-sulfolene (8.7 g, 0.073 mol) were refluxed in acetic acid (50 mL) at 110 °C for 40 h. After removal of the acetic acid under reduced pressure, *trans*-cyclohex-4-ene-1,2-dicarboxylic acid (Compound 1) was collected as a white solid (7.0 g, 96% yield). ^{1}H NMR (400 MHz, DMSO-d6) δ 5.75 – 5.58 (m, 2H), 2.63 – 2.53 (m, 2H), 2.39 – 2.25 (m, 2H), 2.11 – 2.00 (m, 2H). ^{13}C NMR (100 MHz, DMSO-d6) δ 176.34, 125.58, 41.25, 27.96.

4.2. Synthesis of Compound 2

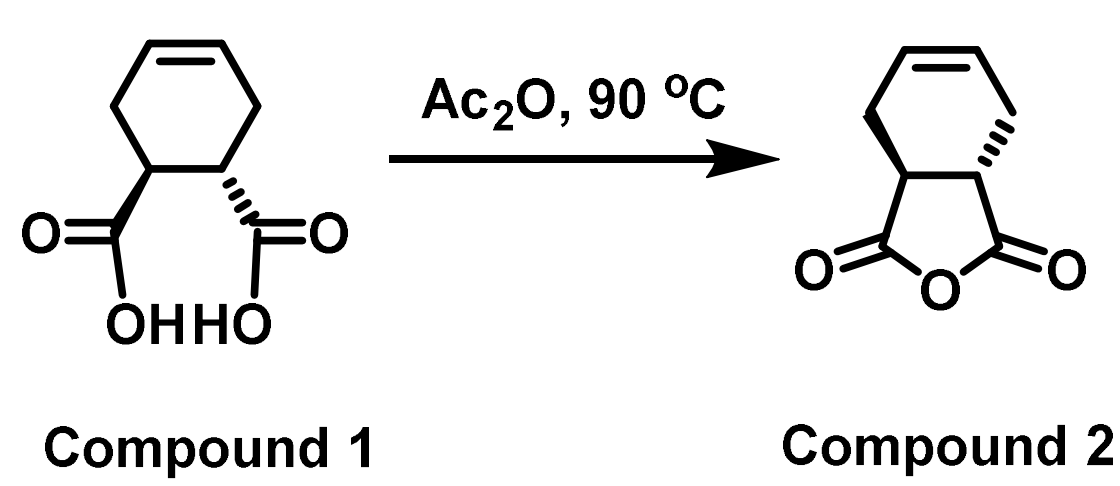

The synthesis of compound 2 was adapted from a previously reported literature procedure.[2] Compound 1 (5.0 g) was heated in acetic anhydride (50 mL) at 90 °C for 5 h. After completion of the reaction, the mixture was cooled to 0 °C. The precipitated anhydride (Compound 2) was collected by filtration, washed with hexane, and dried under reduced pressure to afford Compound 2 as a pale white solid (3.5 g, 78% yield). ^{1}H NMR (400 MHz, $CDCl_3$) δ 5.88 – 5.72

(m, 2H), 2.96 – 2.80 (m, 2H), 2.66 – 2.52 (m, 2H), 2.49–2.34 (m, 2H). ^{13}C NMR (100 MHz, $CDCl_3$) δ 170.31, 126.04, 42.93, 25.30.

4.3. Synthesis of M1

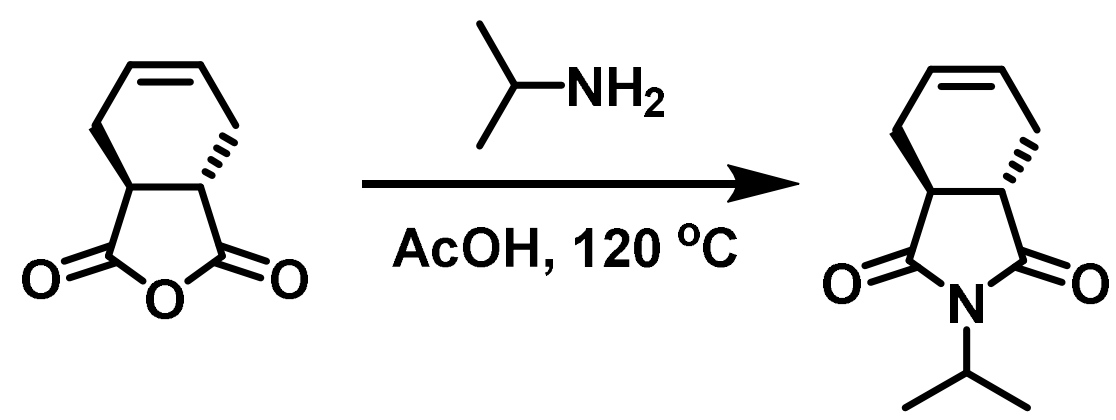

Compound 2 M1

To a solution of isopropylamine (1.6 mL, 19 mmol) in acetic acid (60 mL) was added Compound 2 (2.4 g, 16 mmol). The reaction mixture was stirred at 120 °C for 5 h and then cooled to room temperature. The acetic acid was removed under reduced pressure using rotary evaporation. The crude product was purified by column chromatography (ethyl acetate/hexane, 10:90, v/v) to afford M1 as a white solid (0.6 g, 20% yield). ^{1}H NMR (400 MHz, $CDCl_3$) δ 5.88 – 5.67 (m, 2H), 4.33 (hept, J = 6.9 Hz, 1H), 2.62 – 2.41 (m, 4H), 2.37 – 2.15 (m, 2H), 1.36 (dd, J = 6.9, 3.1 Hz, 6H). ^{13}C NMR (100 MHz, $CDCl_3$) δ 176.88, 126.80, 43.31, 43.05, 25.45, 20.05, 19.00.

4.4. Synthesis of M2

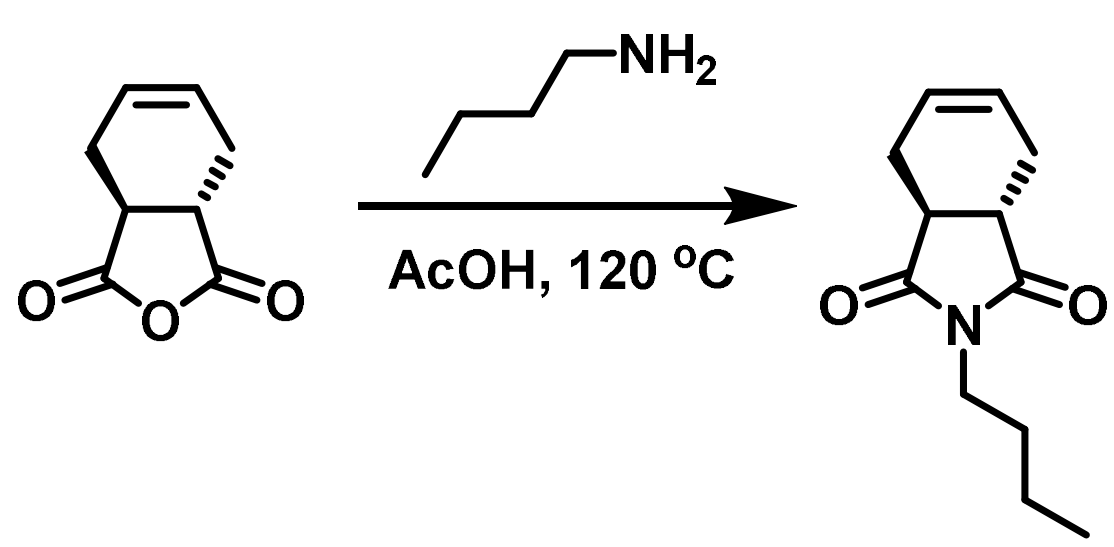

Compound 2 M2

To a solution of n-butylamine (1.5 mL, 15 mmol) in acetic acid (50 mL) was added Compound 2 (2.0 g, 13 mmol). The reaction mixture was stirred at 120 °C for 5 h and then cooled to room

temperature. The acetic acid was removed under reduced pressure using rotary evaporation. The crude product was purified by column chromatography (ethyl acetate/hexane, 10:90, v/v) to afford M2 as a white crystaline solid (1.1 g, 40% yield). ^{1}H NMR (400 MHz, $CDCl_3$) δ 5.90 – 5.63 (m, 2H), 3.61 – 3.32 (m, 2H), 2.61 – 2.47 (m, 4H), 2.39 – 2.16 (m, 2H), 1.59 – 1.42 (m, 2H), 1.38 – 1.18 (m, 2H), 0.90 (t, J = 7.3 Hz, 3H). ^{13}C NMR (100 MHz, $CDCl_3$) δ 176.87, 126.82, 43.33, 37.99, 30.05, 25.47, 20.09, 13.65.

4.5. Synthesis of M3

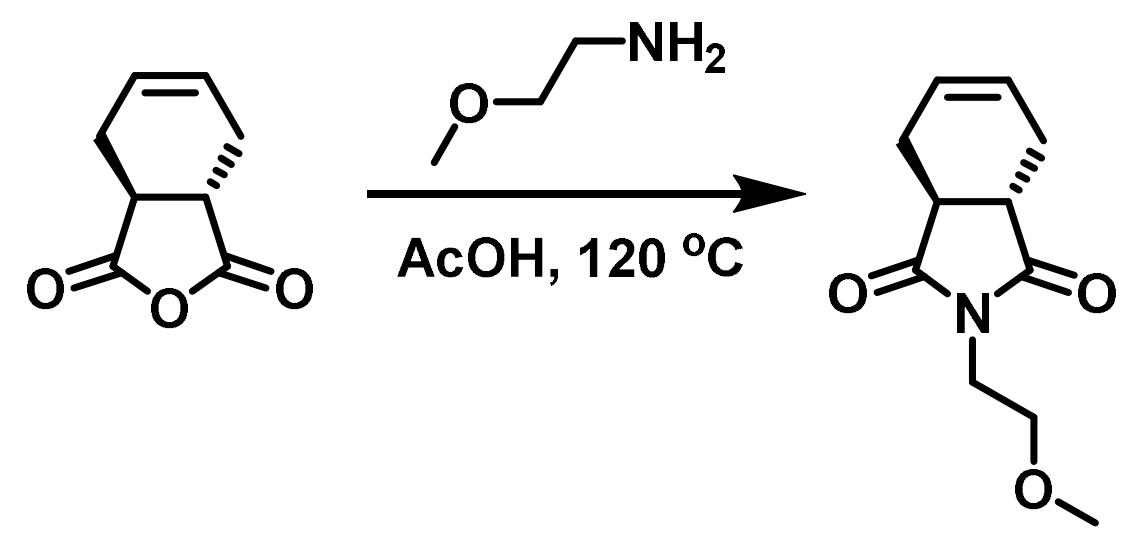

Compound 2 M3

To a solution of 2-methoxyethylamine (2.0 mL, 24 mmol) in acetic acid (50 mL) was added Compound 2 (3.0 g, 20 mmol). The reaction mixture was stirred at 120 °C for 5 h and then cooled to room temperature. The acetic acid was removed under reduced pressure using rotary evaporation. The crude product was purified by column chromatography (ethyl acetate/hexane, 10:90, v/v) to afford M3 as a white crystaline solid (2.4 g, 59% yield). ^{1}H NMR (400 MHz, $CDCl_3$) δ 5.89 – 5.69 (m, 2H), 3.77 – 3.58 (m, 2H), 3.57 – 3.46 (m, 2H), 3.29 (s, 3H), 2.66 – 2.46 (m, 4H), 2.38 – 2.20 (m, 2H). ^{13}C NMR (100 MHz, $CDCl_3$) δ 176.78, 126.77, 68.93, 58.57, 43.24, 37.40, 25.40.

4.6. Synthesis of M4

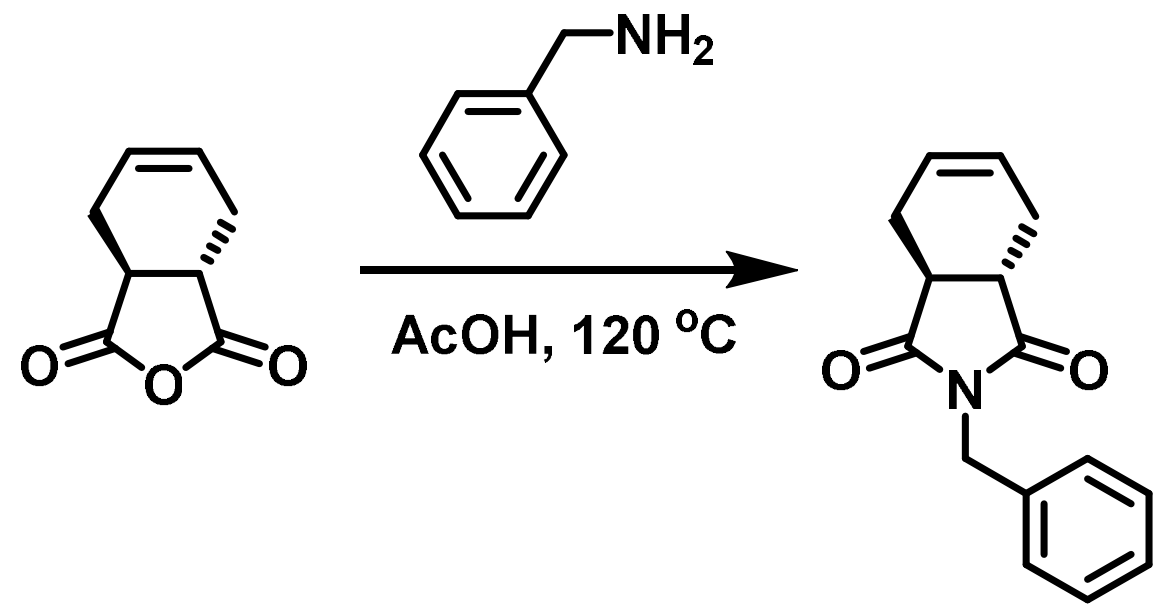

Compound 2 **M4**

To a solution of benzylamine (1.7 g, 16 mmol) in acetic acid (50 mL) was added Compound 2 (2.0 g, 13 mmol). The reaction mixture was stirred at 120 °C for 5 h and then cooled to room temperature. The acetic acid was removed under reduced pressure using rotary evaporation. The crude product was purified by column chromatography (ethyl acetate/hexane, 10:90, v/v) to afford M4 as a white solid (1.4 g, 43% yield). ^{1}H NMR (400 MHz, $CDCl_3$) δ 7.45 – 7.19 (m, 5H), 5.89 – 5.71 (m, 2H), 4.74 – 4.53 (m, 2H), 2.69 – 2.48 (m, 4H), 2.42 – 2.20 (m, 2H). ^{13}C NMR (100 MHz, $CDCl_3$) δ 176.44, 136.19, 128.72, 127.88, 126.76, 43.39, 41.85, 25.40.

4.7. Synthesis of M5

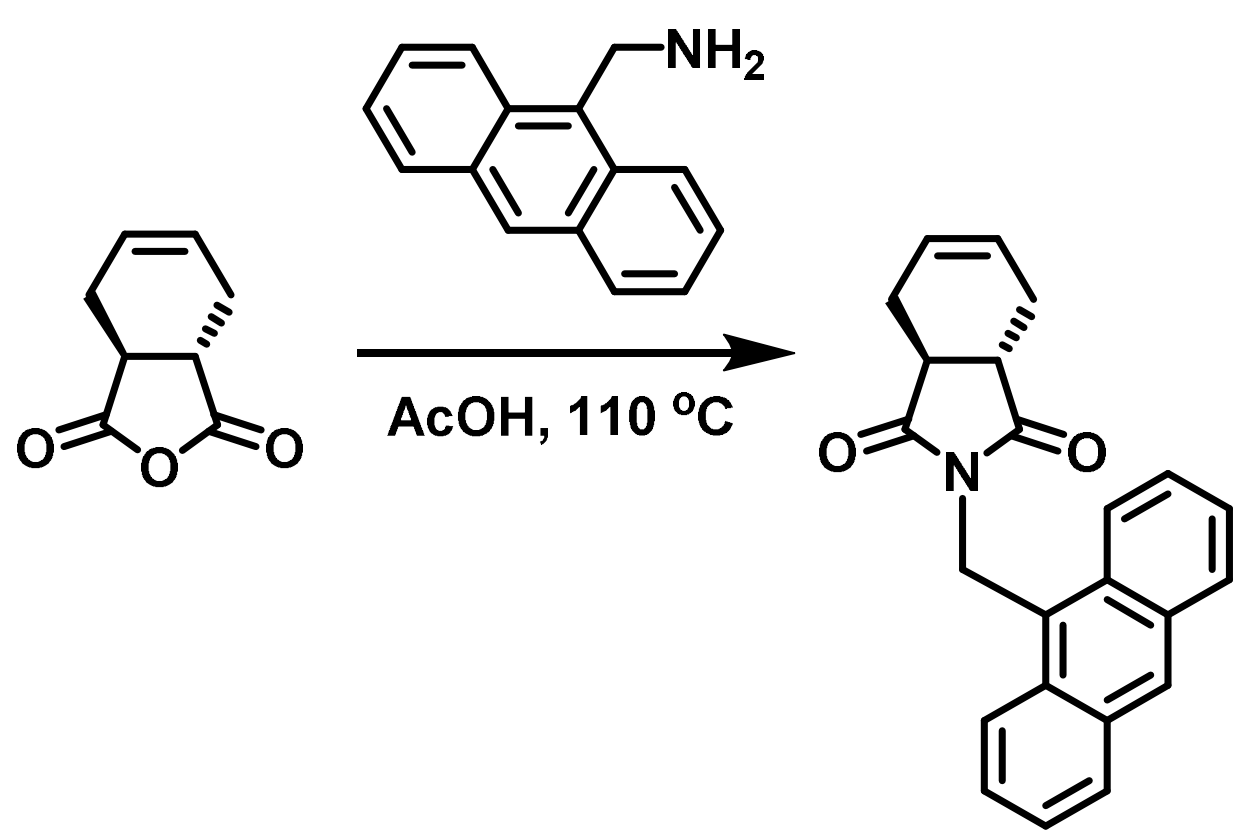

Compound 2 **M5**

Compound 2 (0.70 g, 4.6 mmol) was dissolved in DMSO (15 mL). Under continuous stirring, anthracen-9-ylmethanamine (1.0 g, 5.1 mmol) was added, followed by acetic acid (50 mL). The

reaction mixture was heated at 110 °C for 24 h and then cooled to room temperature. The acetic acid was removed under reduced pressure, and the resulting residue was extracted with ethyl acetate using liquid–liquid extraction. The combined organic layers were dried over anhydrous sodium sulfate, filtered, and concentrated under reduced pressure. The crude product was purified by flash column chromatography (ethyl acetate/hexanes, 10:90, v/v) to afford M5 as a pale yellow crystalline solid (0.63 g, 40% yield). ^{1}H NMR (400 MHz, DMSO-d6) δ 8.62 (s, 1H), 8.51 (d, J = 8.7 Hz, 2H), 8.10 (d, J = 8.0 Hz, 2H), 7.55 (ddd, J = 17.9, 10.6, 6.1 Hz, 4H), 5.73 – 5.66 (m, 2H), 5.55 (s, 2H), 2.74 – 2.65 (m, 2H), 2.35 – 2.12 (m, 4H). ^{13}C NMR (100 MHz, $CDCl_3$) δ 177.42, 131.40, 130.95, 129.35, 128.59, 127.54, 127.20, 126.48, 125.50, 125.23, 42.59, 35.68, 25.21.

4.8. General ROMP Procedure

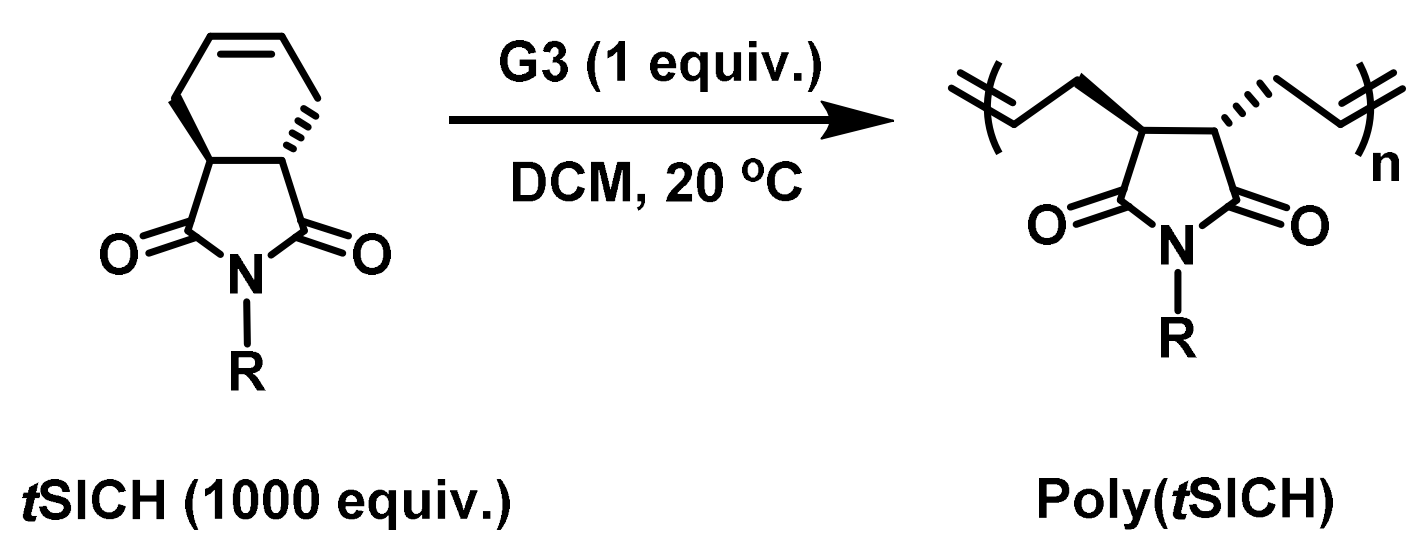

The same general procedure was used for the ROMP of *trans*-succinimide-fused cyclohexene (*t*SICH) monomers (**M1–M4**). In a typical procedure for the synthesis of P1, M1 (386 mg, 2.00 mmol, 1000 equiv.) was dissolved in DCM (0.4 mL) and degassed with argon. Separately, G3 catalyst (1.8 mg, 0.0020 mmol, 1.0 equiv.) was quickly added to the monomer solution. The reaction mixture was stirred for 12 h. Polymerization was quenched with excess ethyl vinyl ether (19 μL, 0.20 mmol, 100 equiv.). The resulting solution was precipitated into cold methanol to afford the polymer.

4.9. Synthesis of Copolymer P3-*r*-P5

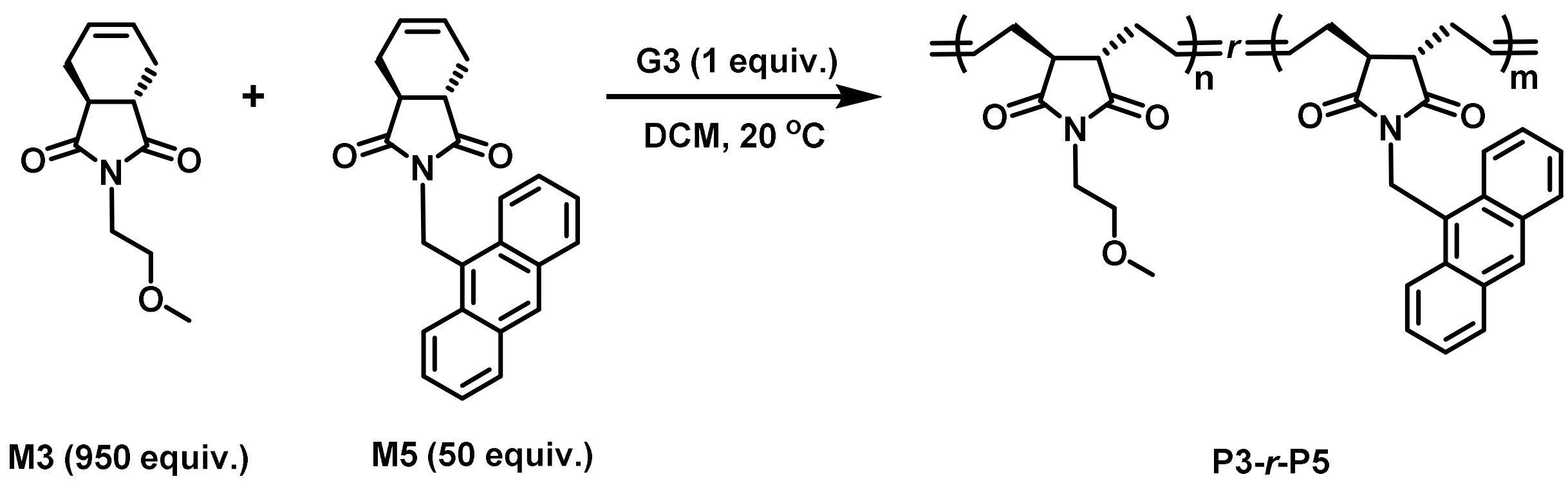

In a typical procedure for the synthesis of P3-*r*-P5 with an M3/M5 feed ratio of 95:5, M3 (400 mg, 1.90 mmol, 950 equiv.) and M5 (34 mg, 0.10 mmol, 50 equiv.) were dissolved in DCM (0.4 mL) and degassed with argon. Separately, G3 catalyst (1.8 mg, 0.0020 mmol, 1.0 equiv.) was quickly added to the monomer solution. The reaction mixture was stirred for 12 h. Polymerization was quenched with excess ethyl vinyl ether (19 µL, 0.20 mmol, 100 equiv.). The resulting solution was precipitated into cold methanol to afford the polymer product.

4.10. Investigation of Polymerization Thermodynamics

To investigate the thermodynamics of polymerization for representative monomers M1 and M4, the reactions were carried out in DCM at various temperatures ranging from 0 °C to 30 °C using 1 mol% of G3 catalyst. The initial monomer concentration ($[M]_0$) was 0.5 M. Each polymerization was allowed to proceed for 12 hours to reach equilibrium. The equilibrium monomer concentrations ($[M]_{eq}$) were determined by ^{1}H NMR and are summarized in Tables S1–S2. By plotting the logarithm of $[M]_{eq}$ against the inverse of temperature, the thermodynamic parameters, including the change in enthalpy of polymerization (ΔH_p) and the standard-state change in entropy (ΔS_p^o), were obtained from the slope and intercept of the van't Hoff plot based on Equation 1.[3]

$$\ln[\mathrm{M}]_{eq} = \frac{\Delta H_p}{\mathrm{R}}\left(\frac{1}{\mathrm{T}}\right) - \frac{\Delta S_p^o}{\mathrm{R}} \qquad \text{Equation 1}$$

By using these thermodynamic parameters, the ceiling temperature at 1M can be calculated using Equation 2.

$$T_c\,(1\text{ M}) = \Delta H_p / \Delta S_p^o \qquad \text{Equation 2}$$

Furthermore, the ceiling temperature at 0.2 M can be calculated using Equation 3.

$$T_c\,(0.2\text{ M}) = \Delta H_p / \Delta S_p(0.2\ M). \qquad \text{Equation 3}$$

ΔS_p(0.2 M) was calculated according to the Equation 4 according to a previous report,[4] where $[\mathbf{M}]_o$ = 1.0 M and $[\mathbf{M}]$ = 0.2 M.

$$\Delta S_p([\mathbf{M}]) = \Delta S_p^o - R\ln\left(\frac{[\mathbf{M}]_o}{[\mathbf{M}]}\right) \qquad \text{Equation 4}$$

4.11. Depolymerization Procedure

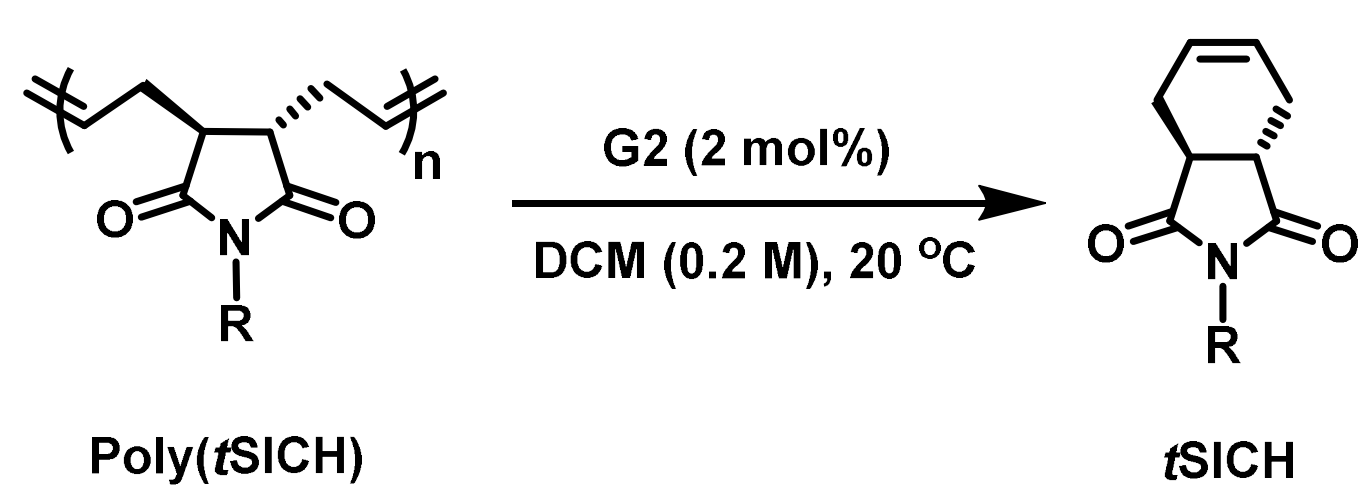

In a typical procedure for the ring-closing metathesis depolymerization of polymers at a concentration of 0.2 M in olefin groups, the polymer (0.10 mmol of backbone olefin groups, 50 equiv.) and G2 (1.7 mg, 0.002 mmol, 1.0 equiv.) were dissolved in DCM (0.5 mL) and degassed with argon. The reaction mixture was stirred at 20 °C for 12 h. Depolymerization was quenched with excess ethyl vinyl ether (20 μL, 0.20 mmol, 100 equiv.). After quenching for 20 min, the solvent was removed under reduced pressure, and the resulting residue was redissolved in $CDCl_3$ for NMR analysis.

4.12. Photoinduced Crosslinking of P3-*r*-P5

In a typical procedure for the solution-phase crosslinking of P3-*r*-P5, P3-*r*-P5 (50 mg) was dissolved in DCM (1 mL) and irradiated under a 365 nm UV lamp. The crosslinking process was monitored using UV–Vis spectroscopy.

4.13. Thermally Induced Decrosslinking

In a typical procedure for the decrosslinking of the polymer network, the crosslinked P3-*r*-P5 film was placed in an oven at 110 °C. The degree of decrosslinking was monitored using UV–Vis spectroscopy.

5. Supporting Figures and Tables

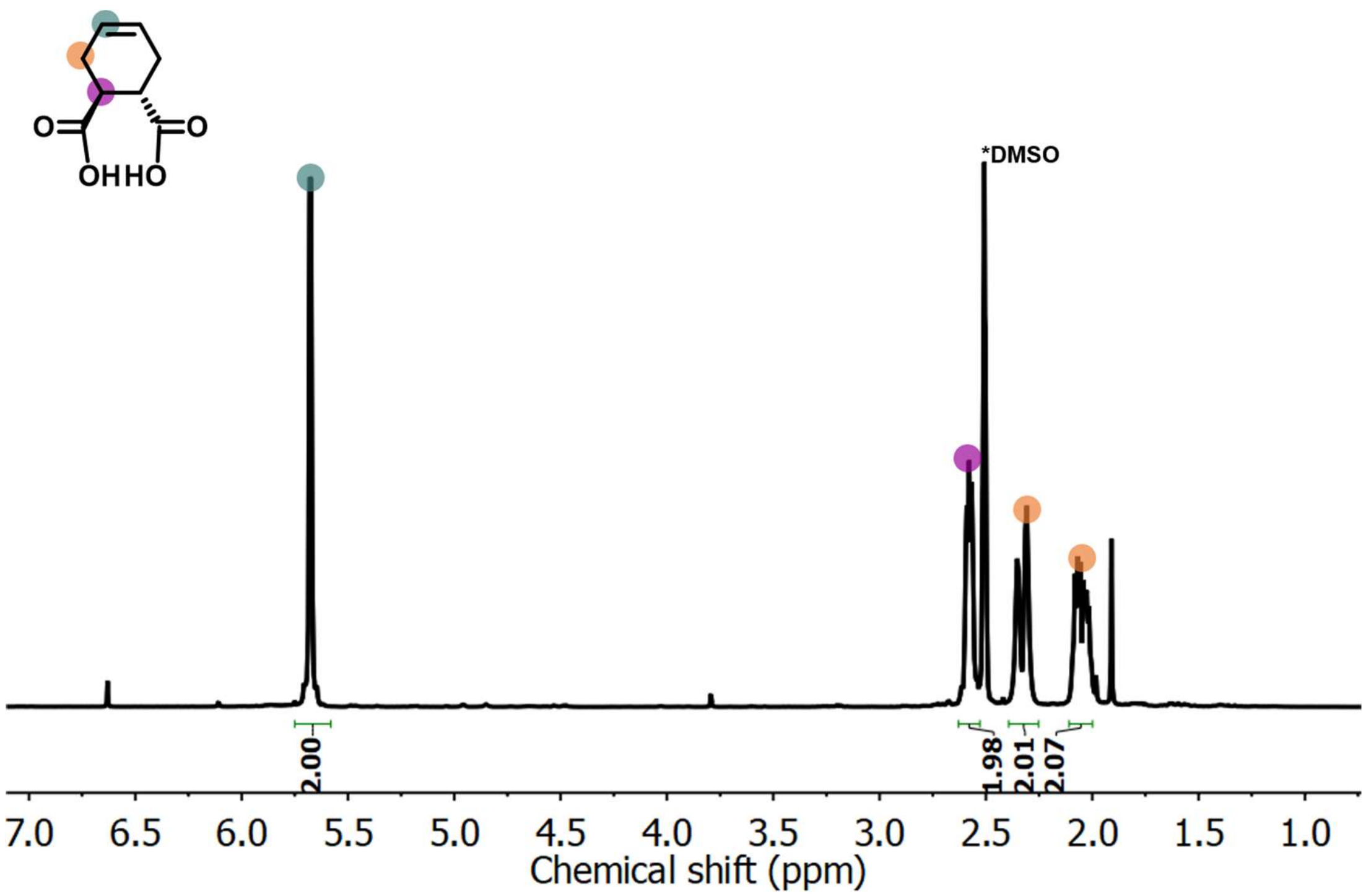

Figure S1. ^{1}H NMR spectrum of compound 1 in DMSO-d_6.

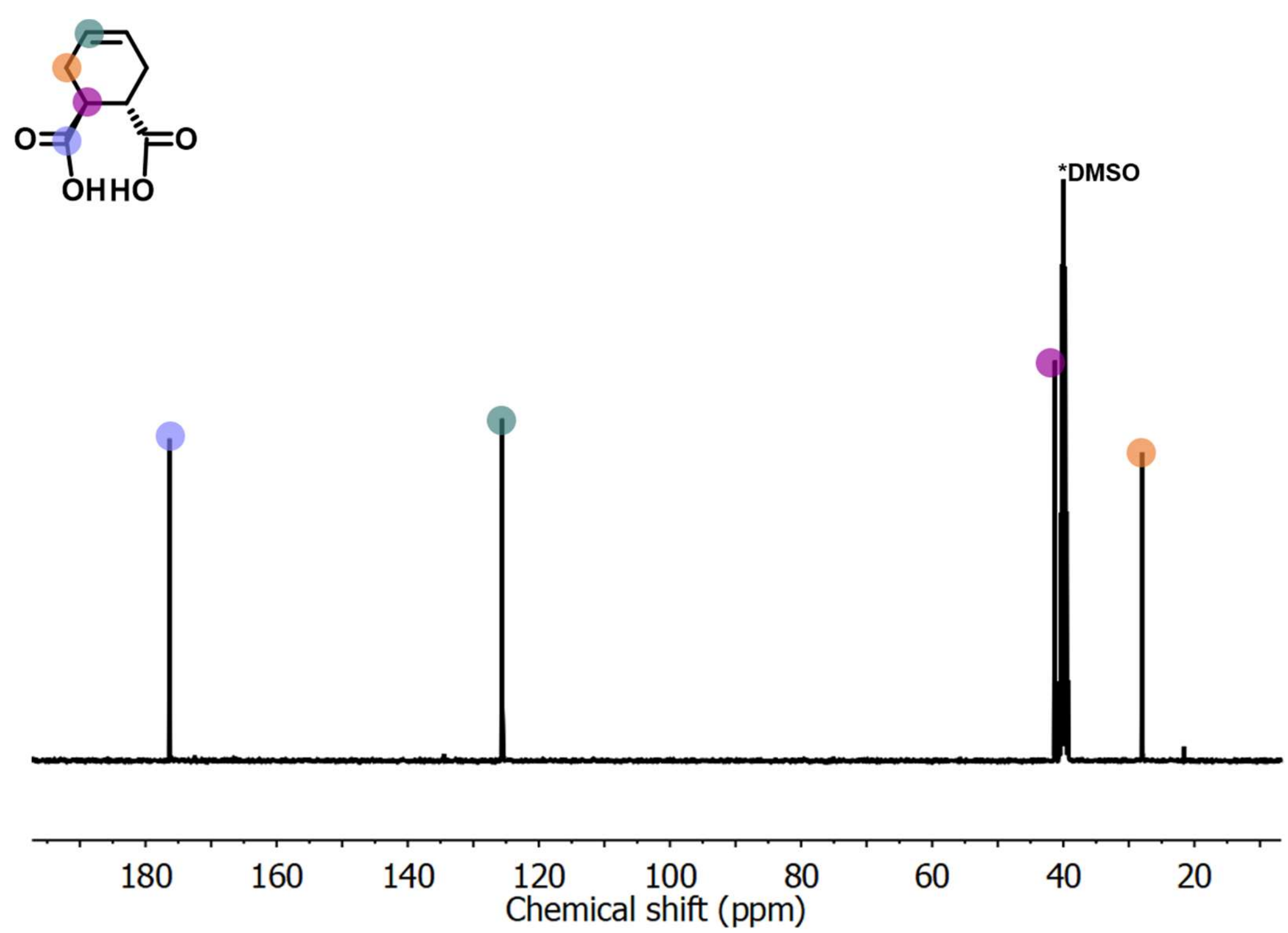

Figure S2. ^{13}C NMR spectrum of compound 1 in DMSO-d_6.

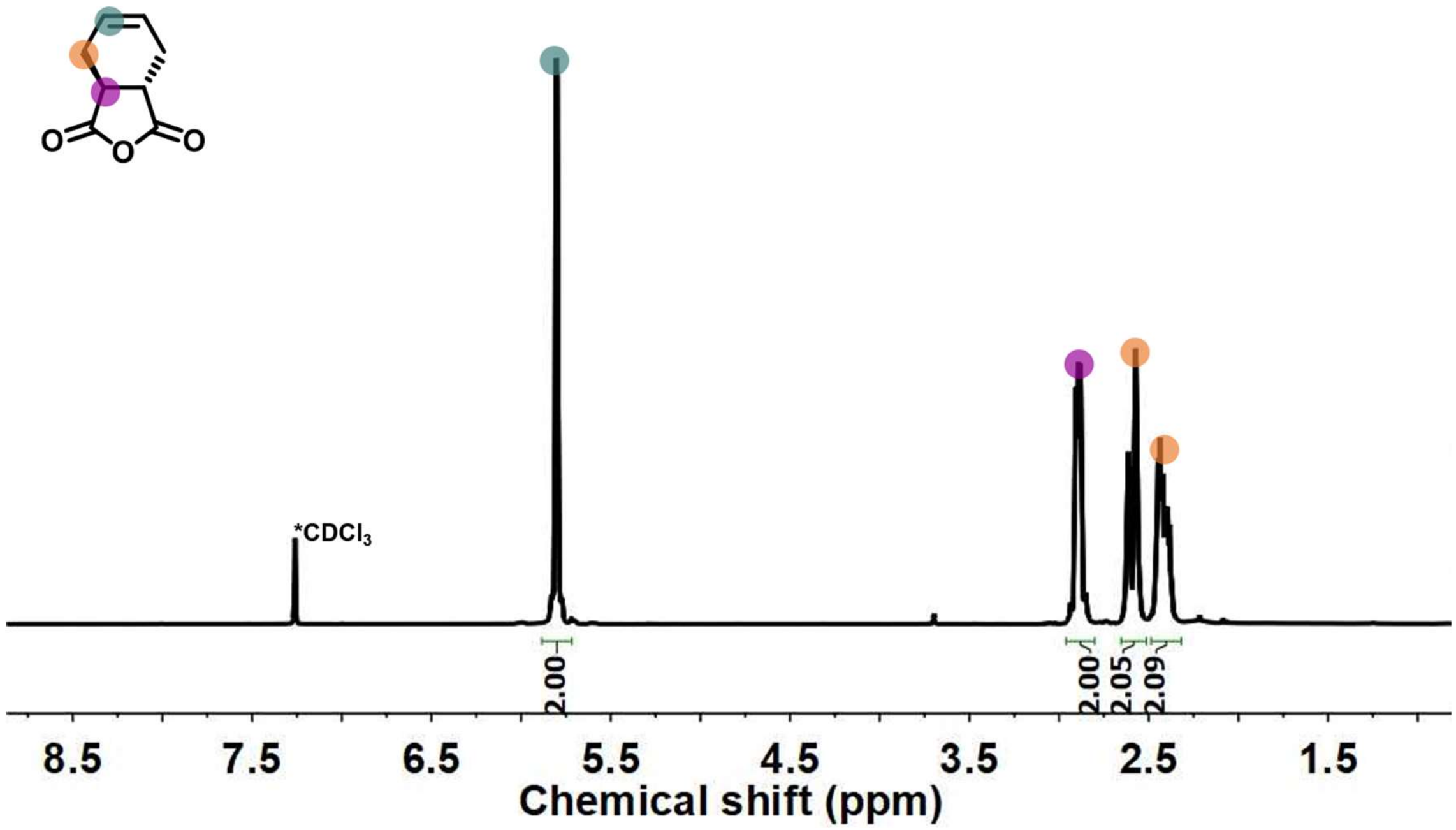

Figure S3. ^{1}H NMR spectrum of compound 2 in $CDCl_3$.

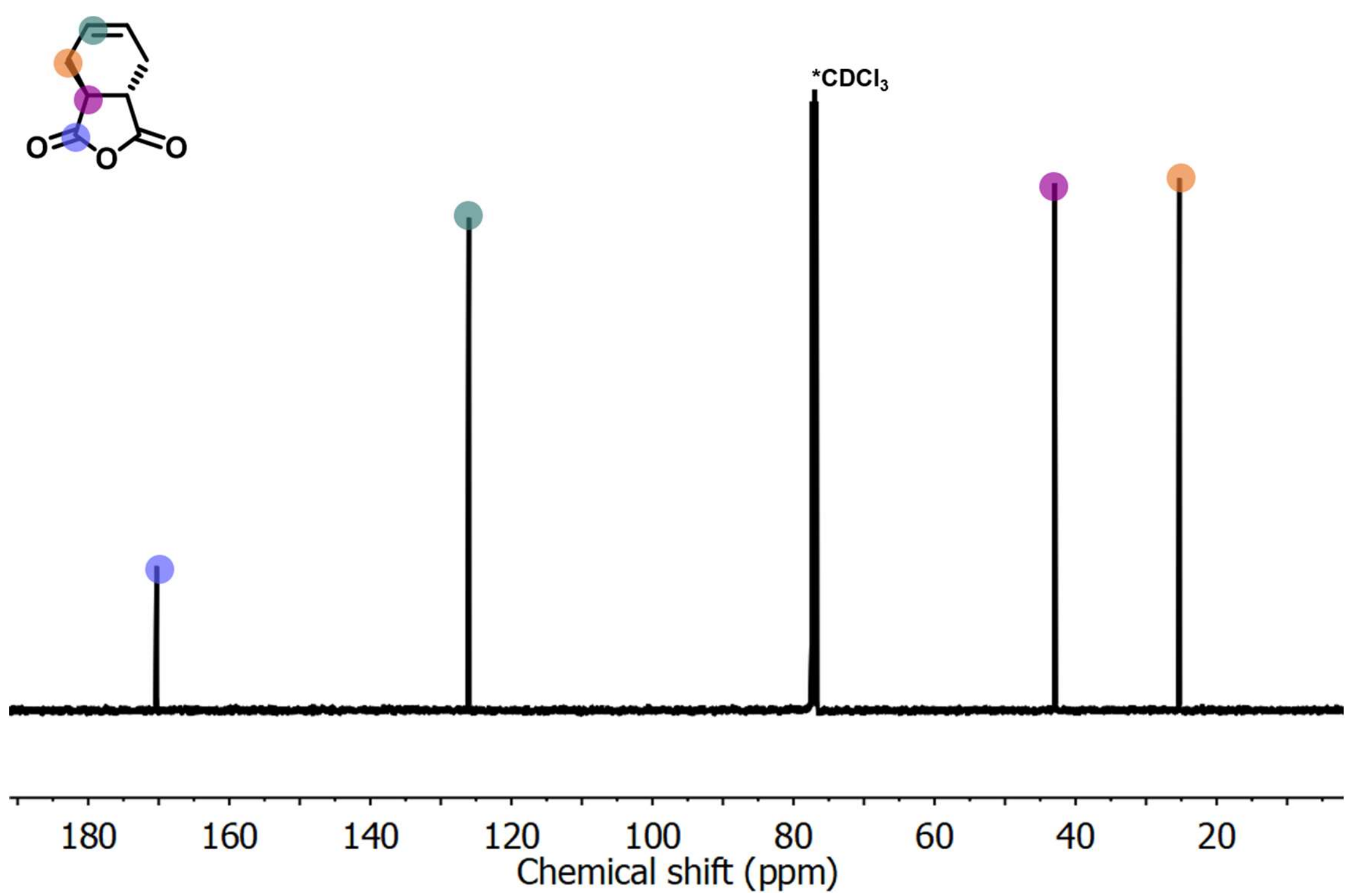

Figure S4. ^{13}C NMR spectrum of compound 2 in $CDCl_3$.

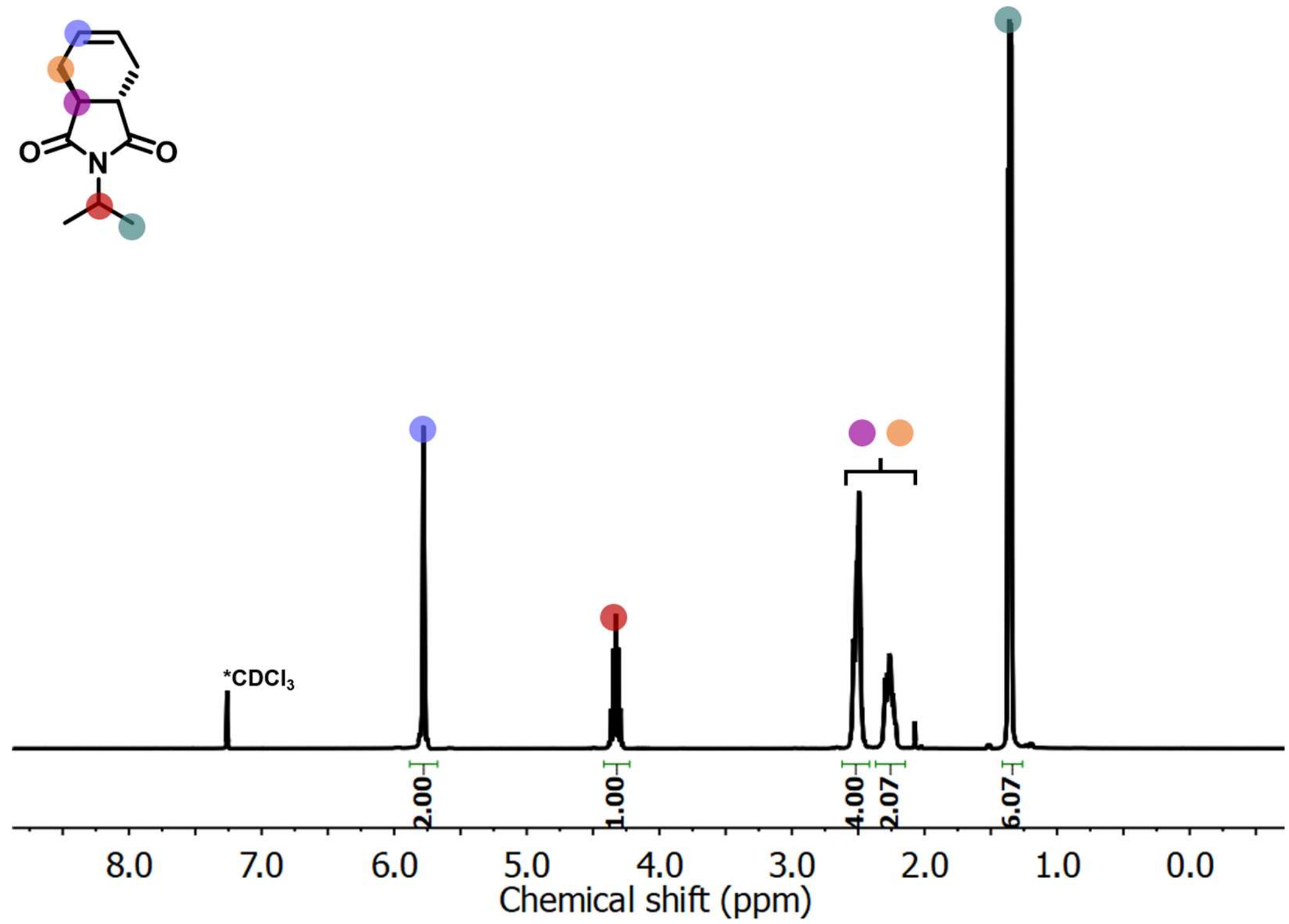

Figure S5. ^{1}H NMR spectrum of M1 in $CDCl_3$.

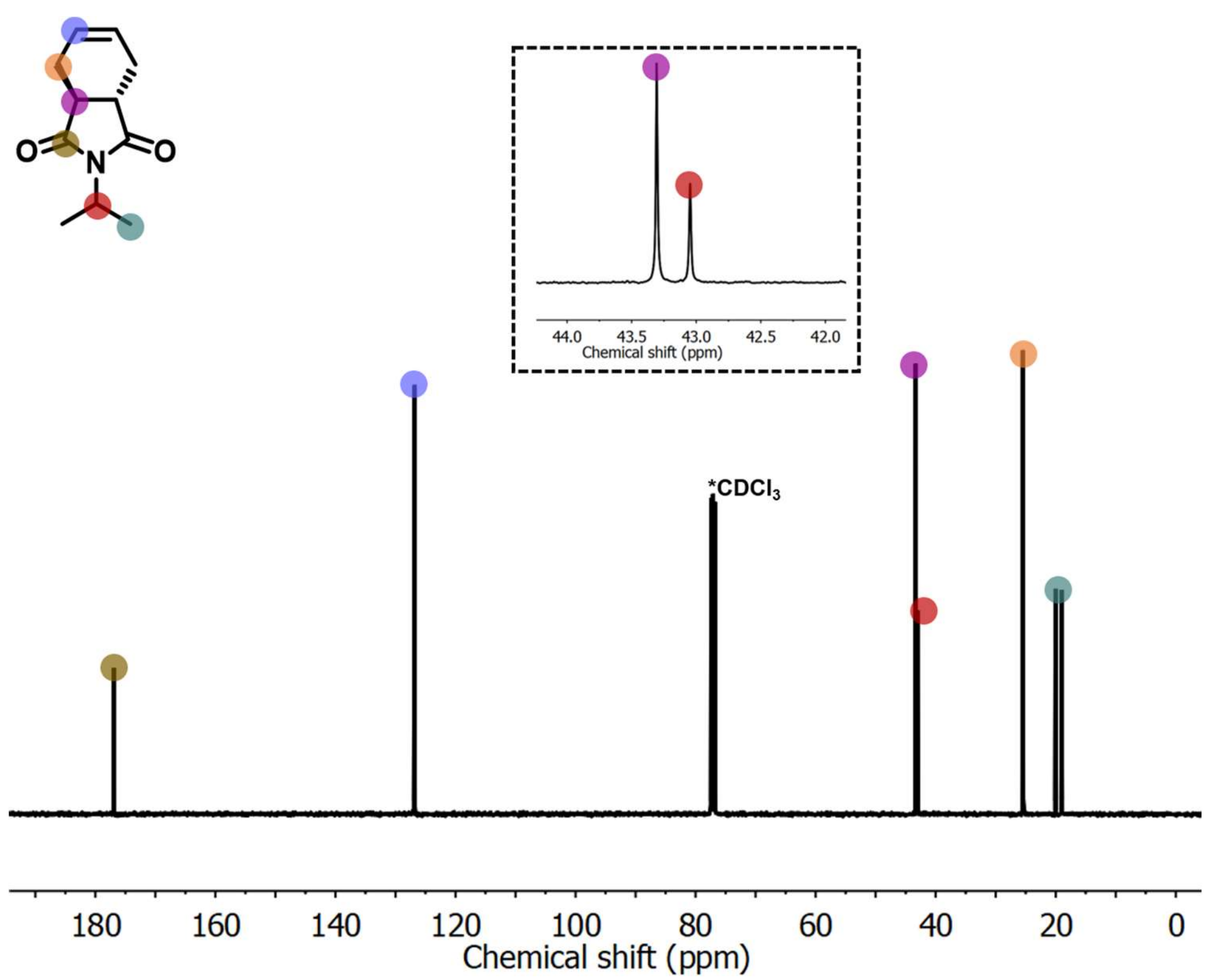

Figure S6. ^{13}C NMR spectrum of M1 in $CDCl_3$.

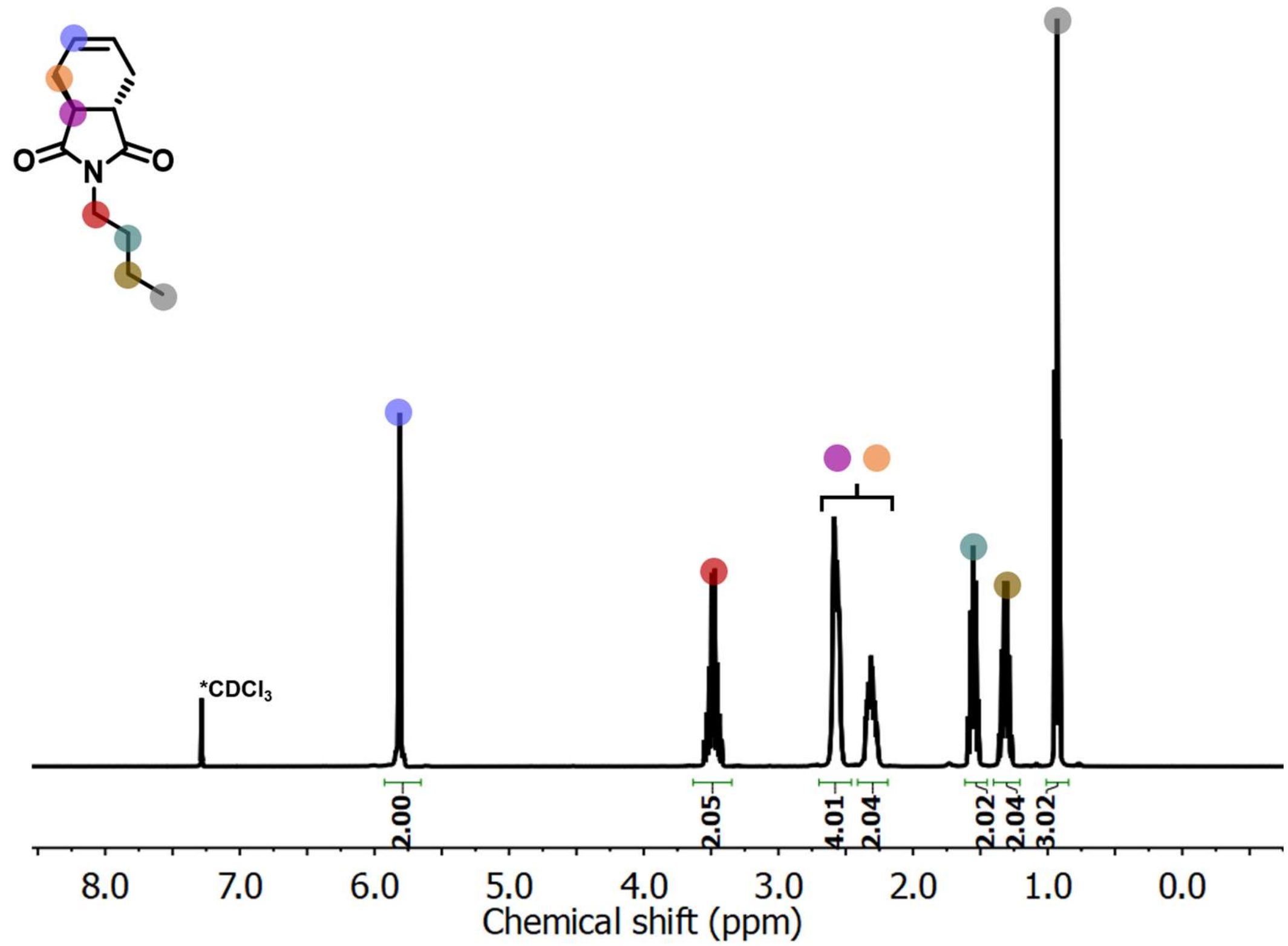

Figure S7. ^{1}H NMR spectrum of M2 in $CDCl_3$.

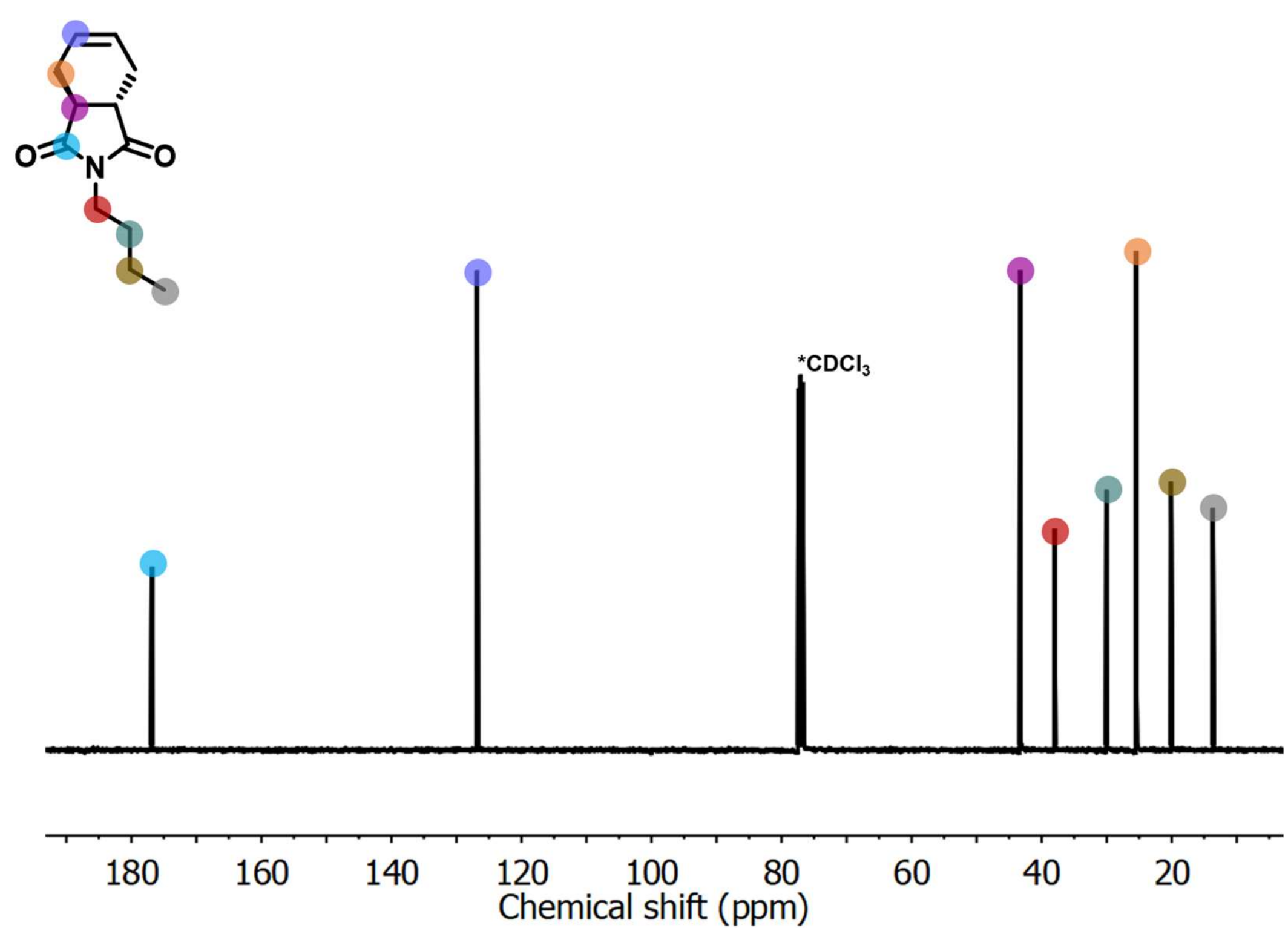

Figure S8. ^{13}C NMR spectrum of M2 in $CDCl_3$.

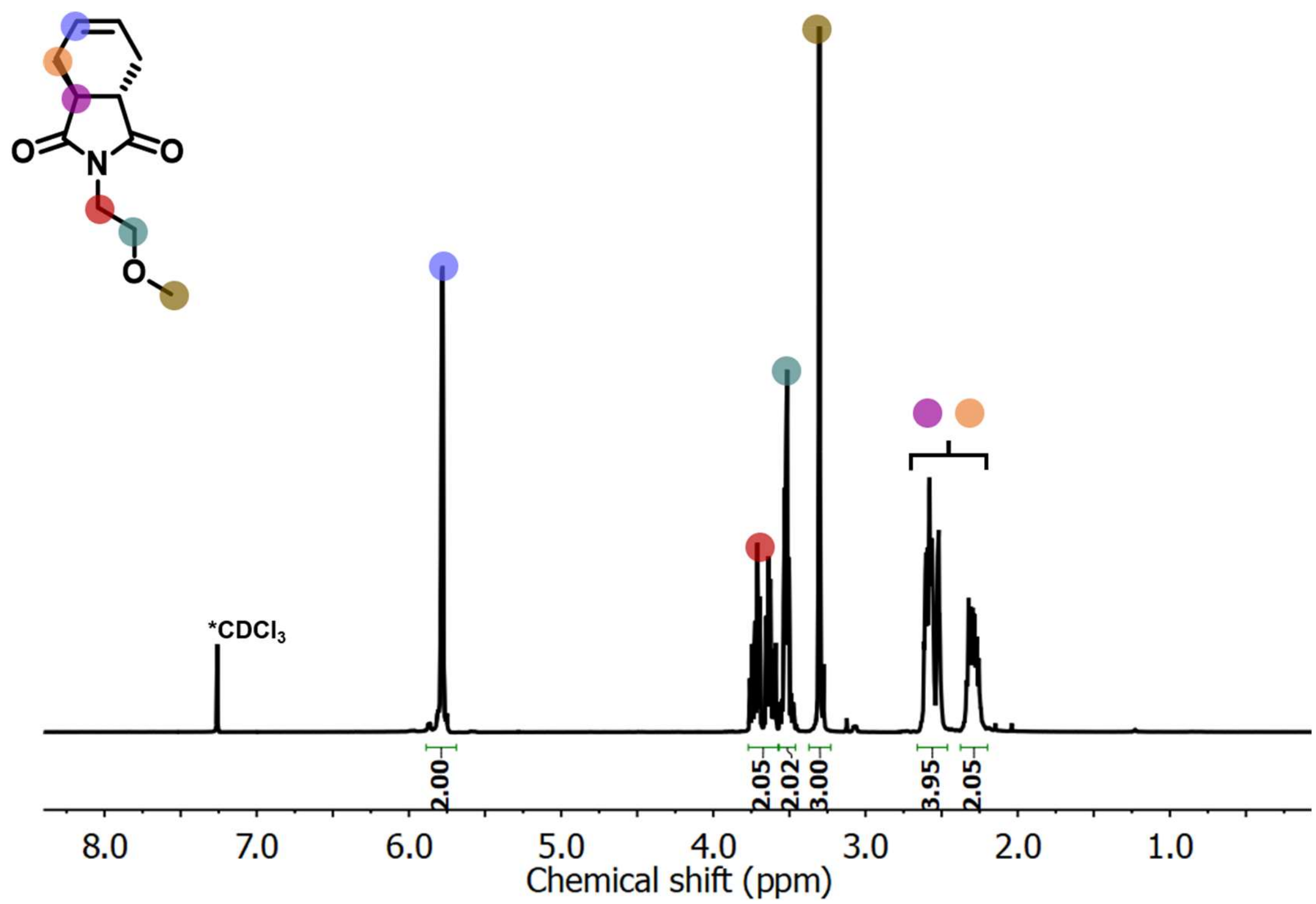

Figure S9. ^{1}H NMR spectrum of M3 in $CDCl_3$.

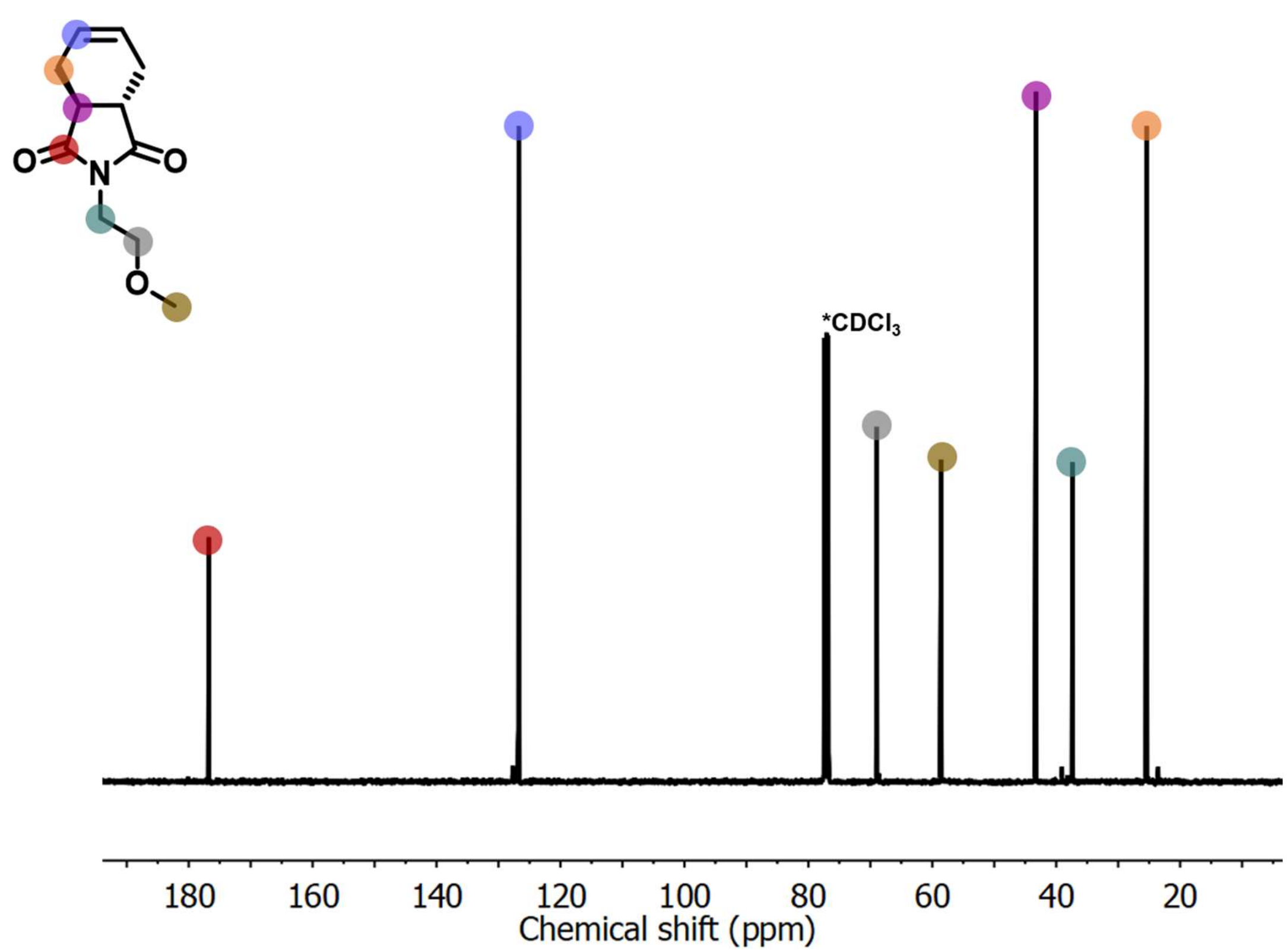

Figure S10. ^{13}C NMR spectrum of M3 in $CDCl_3$.

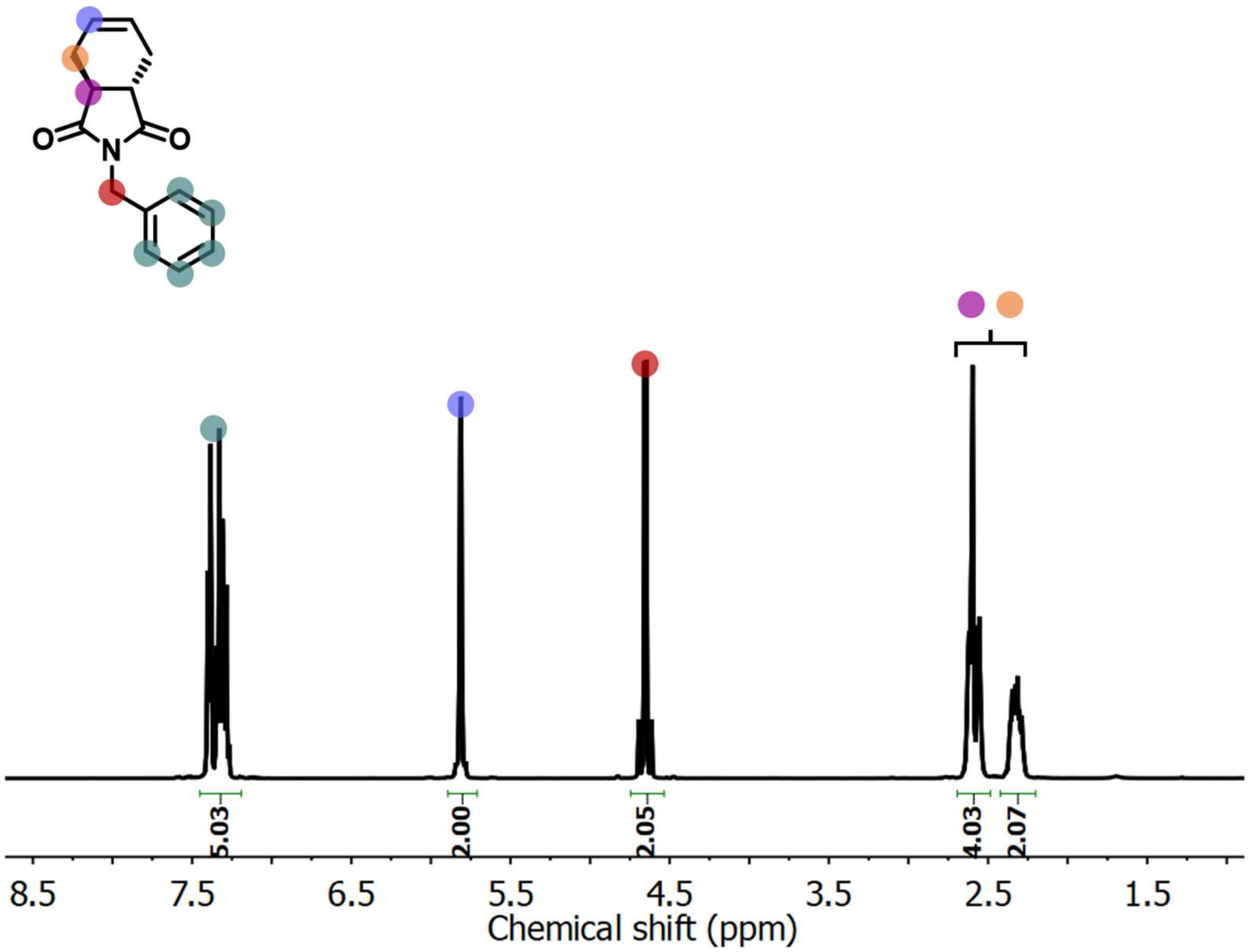

Figure S11. ^{1}H NMR spectrum of M4 in $CDCl_3$.

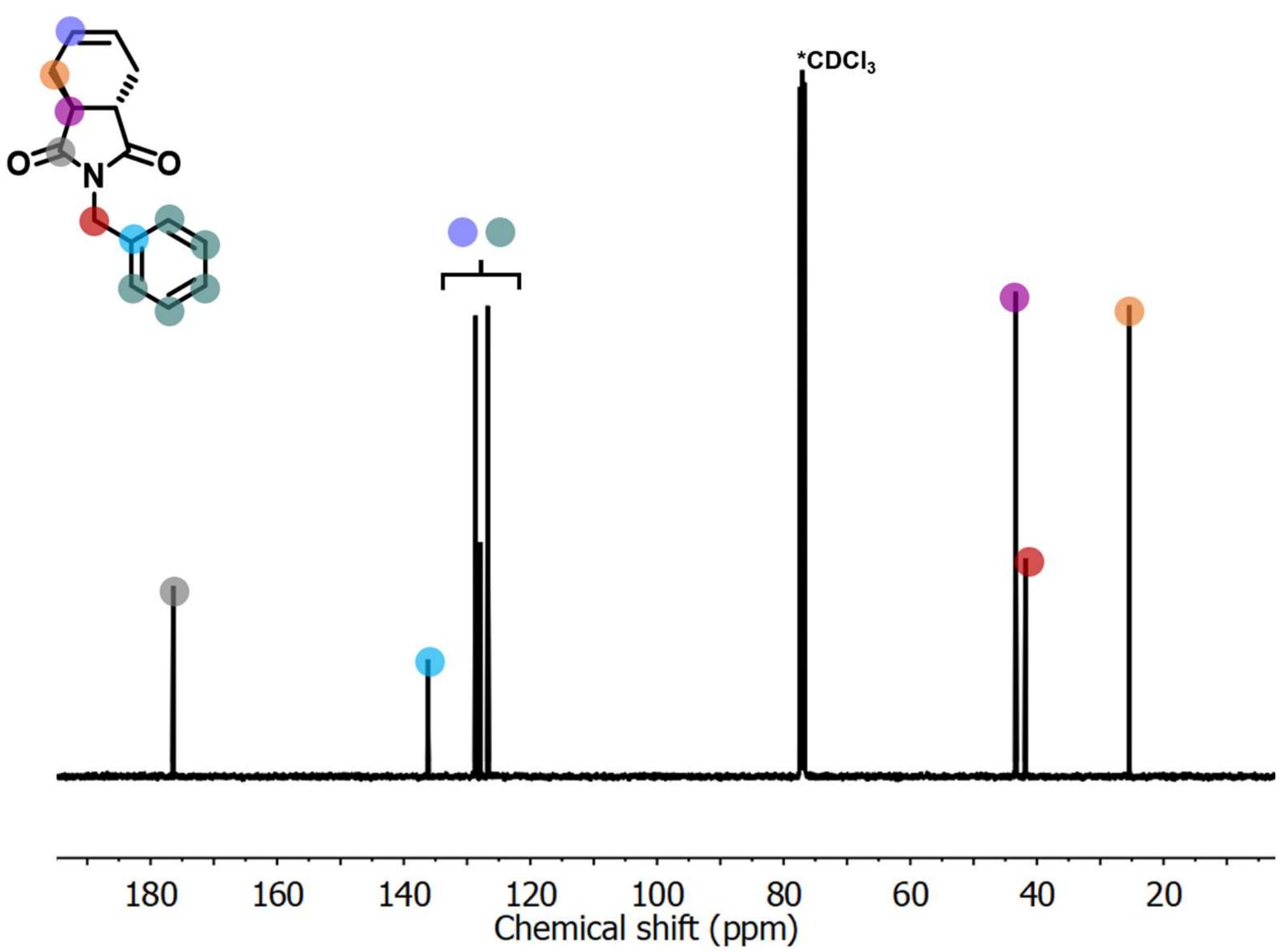

Figure S12. ^{13}H NMR spectrum of M4 in $CDCl_3$.

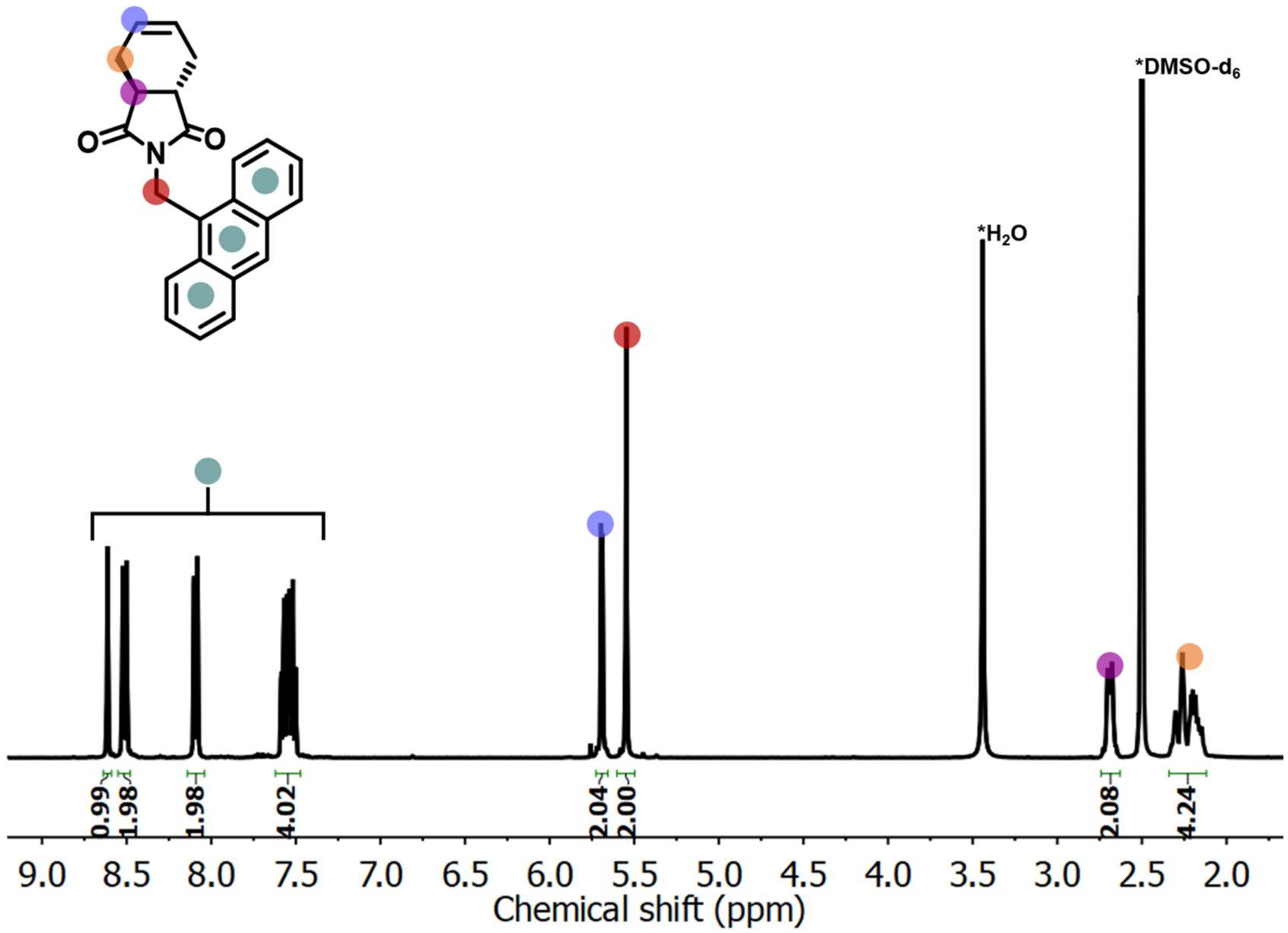

Figure S13. ^{1}H NMR spectrum of M5 in DMSO-d_6.

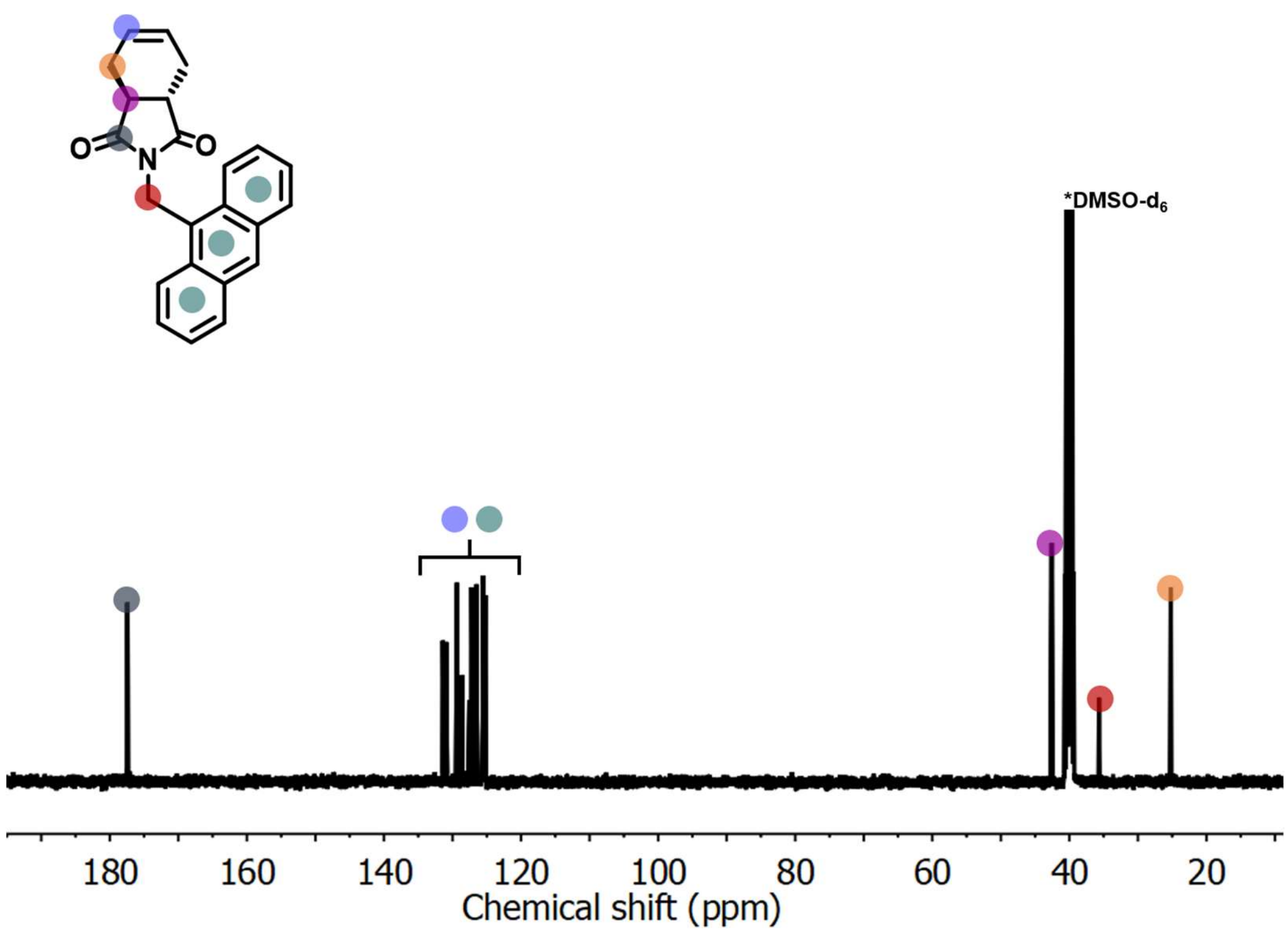

Figure S14. ^{13}C NMR spectrum of M5 in DMSO-d_6.

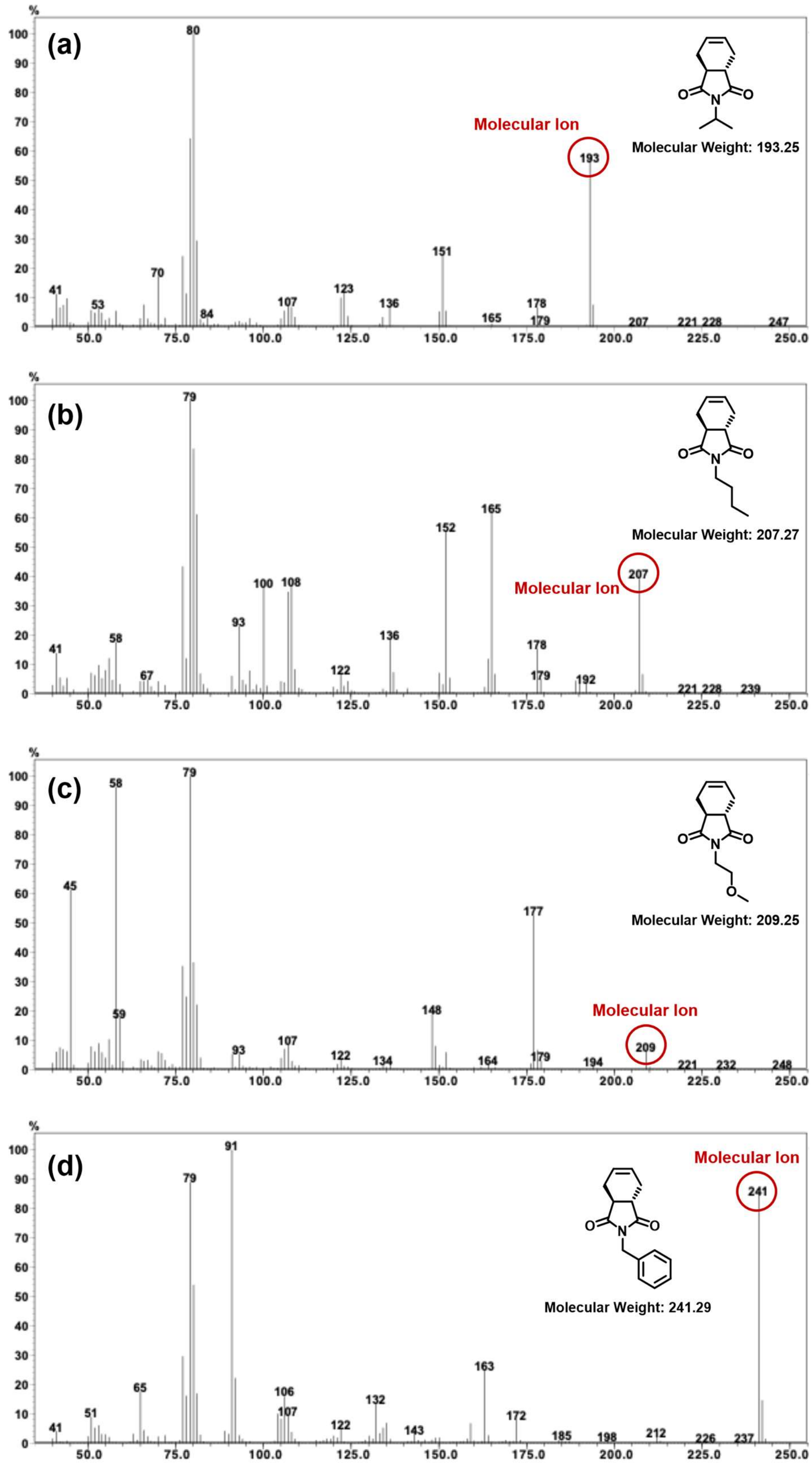

Figure S15. Electron impact mass spectra of M1 (a), M2 (b), M3 (c), and M4 (d).

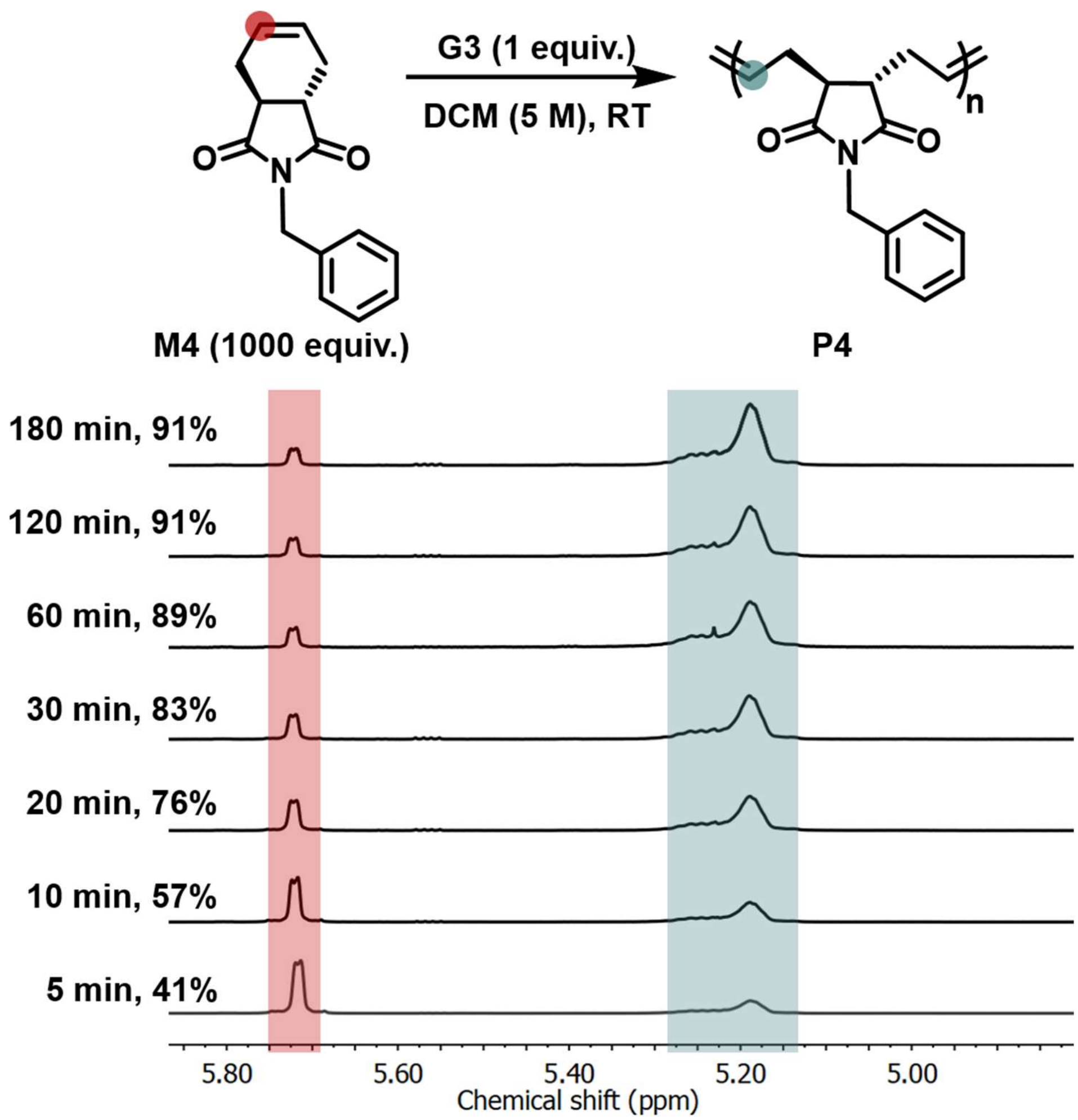

Figure S16. Partial ^{1}H NMR spectra for the polymerization kinetics of M4 (5M) in DCM at 20 °C. The polymerization reached equilibrium within 120 min.

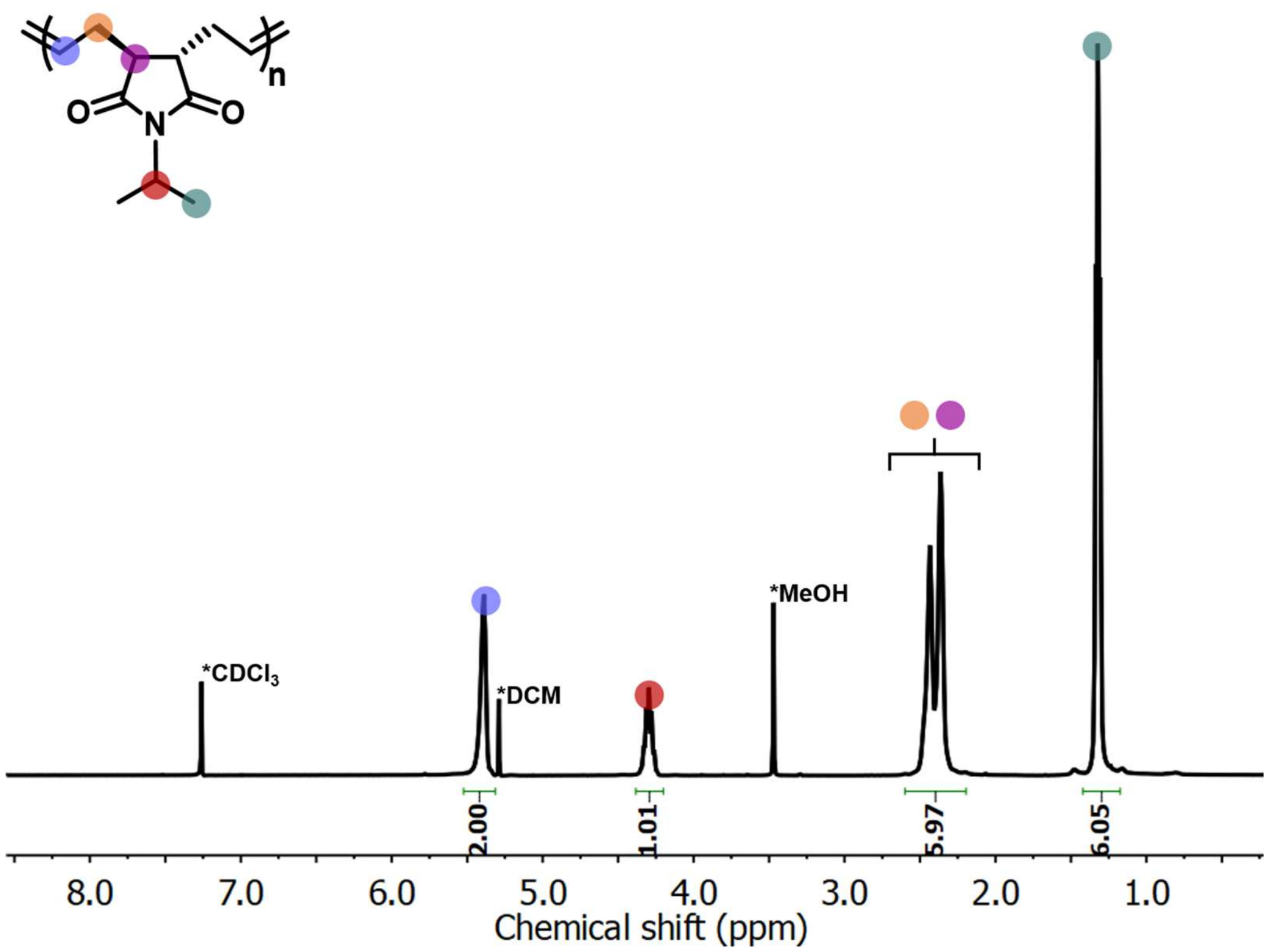

Figure S17. ^{1}H NMR spectrum of P1 in $CDCl_3$.

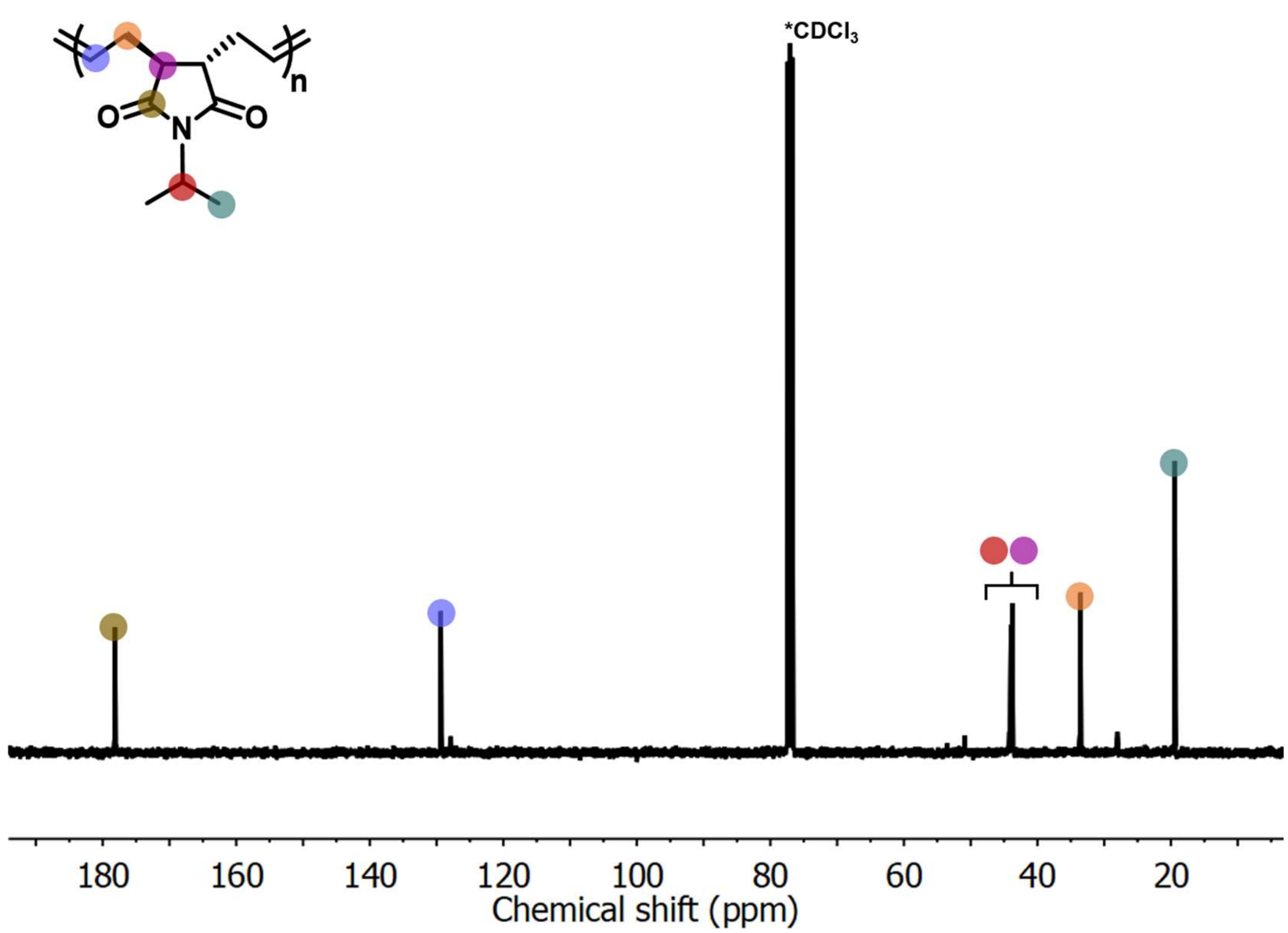

Figure S18. ^{13}C NMR spectrum of P1 in $CDCl_3$.

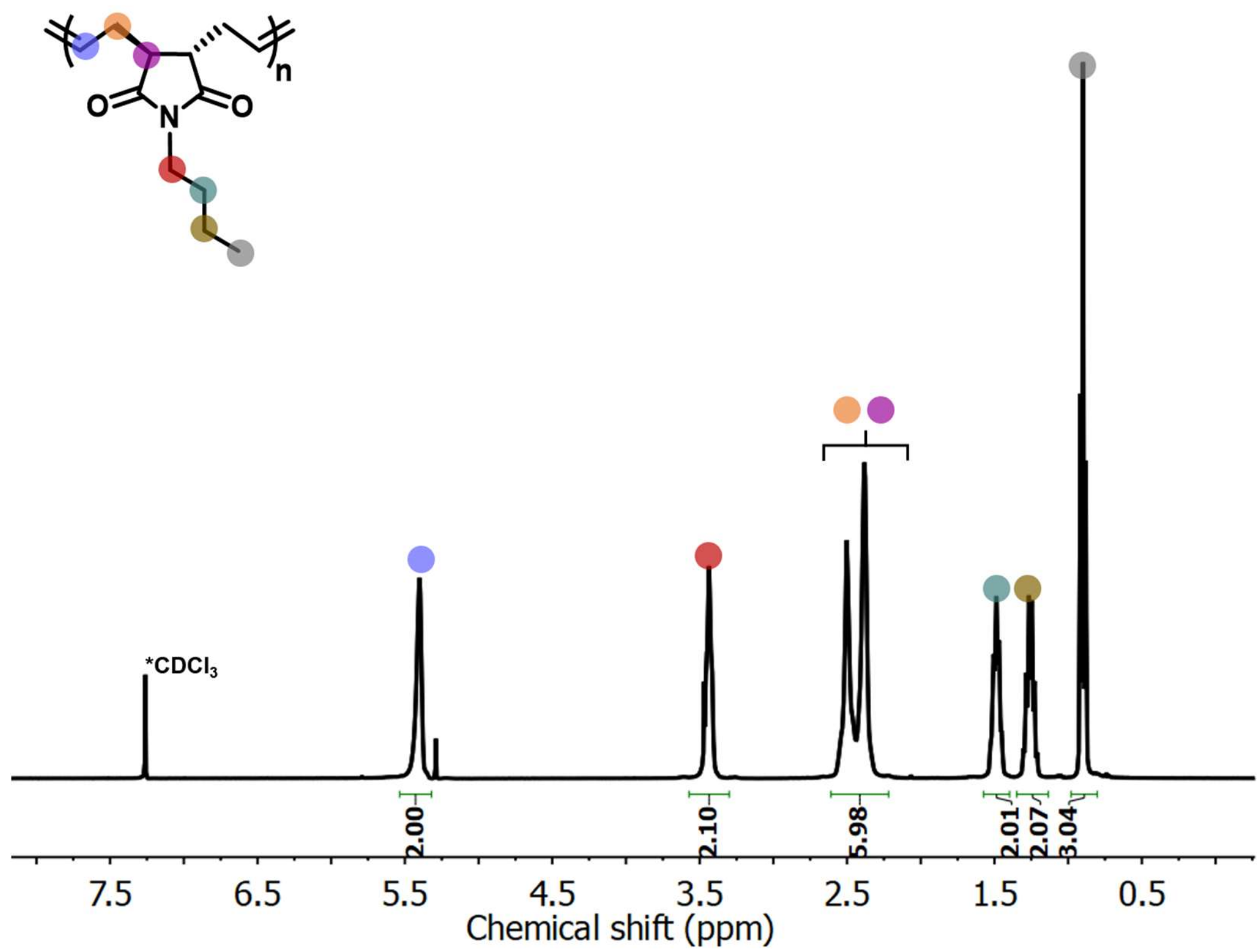

Figure S19. ^{1}H NMR spectrum of P2 in $CDCl_3$.

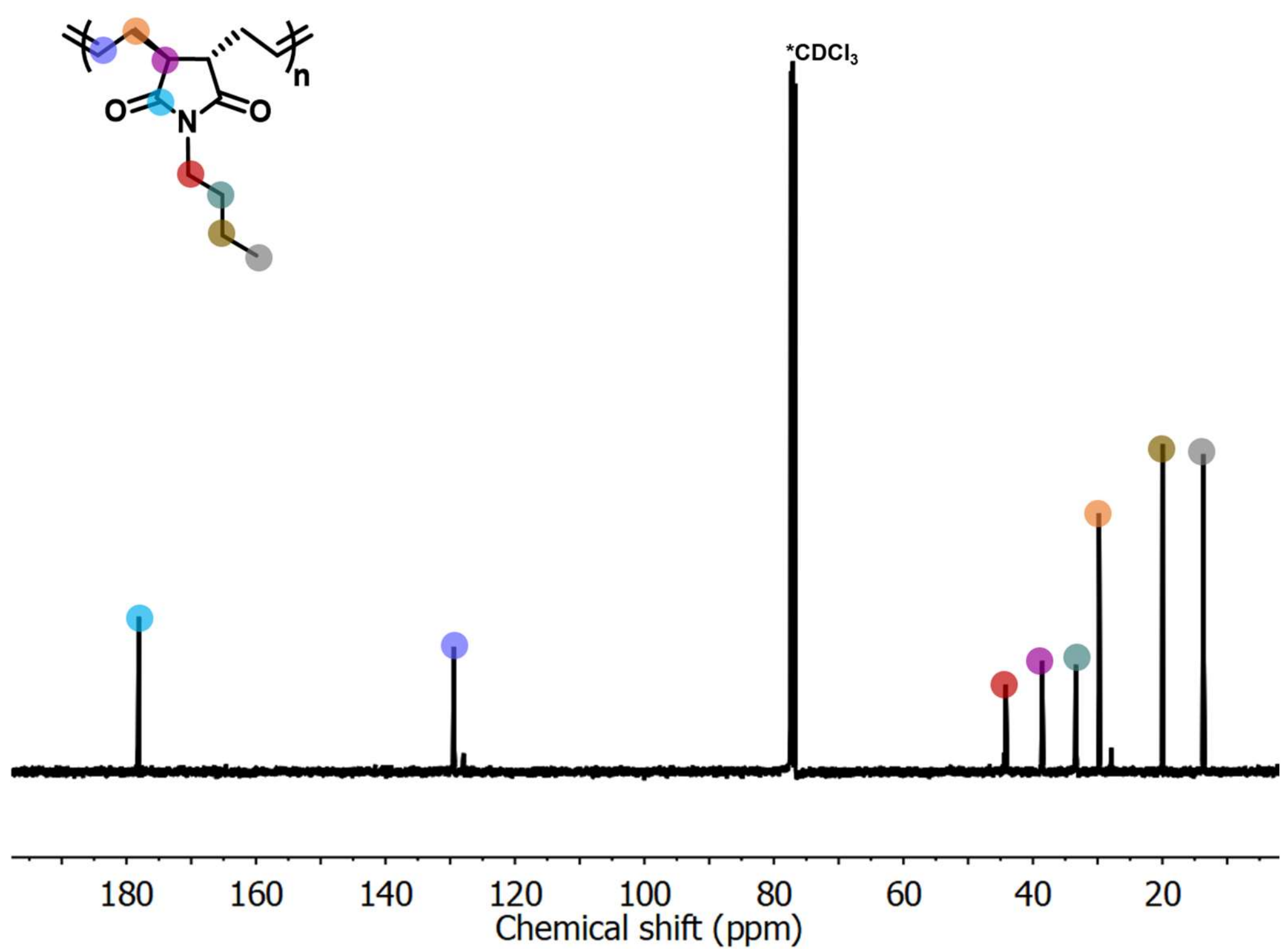

Figure S20. ^{13}C NMR spectrum of P2 in $CDCl_3$.

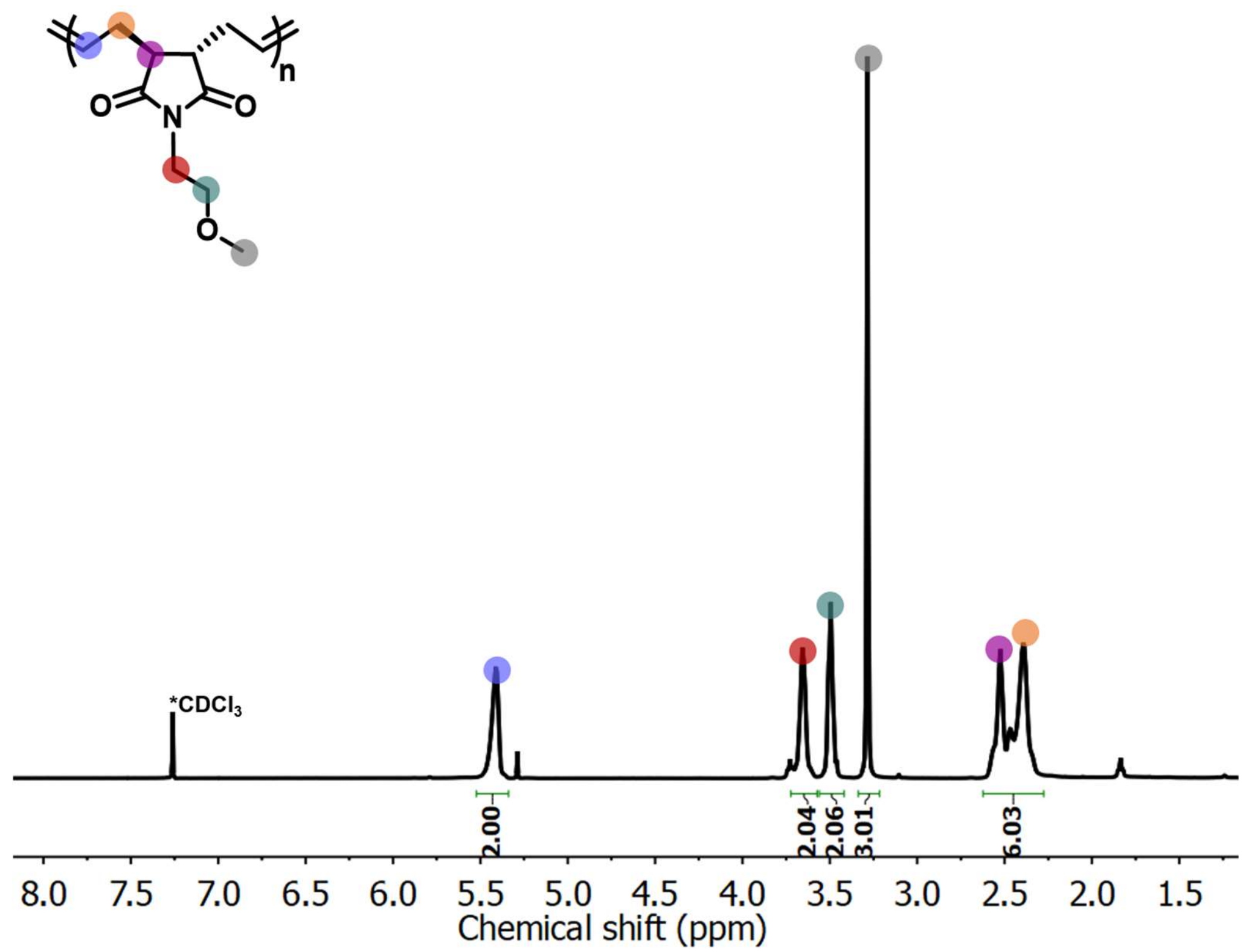

Figure S21. ^{1}H NMR spectrum of P3 in $CDCl_3$.

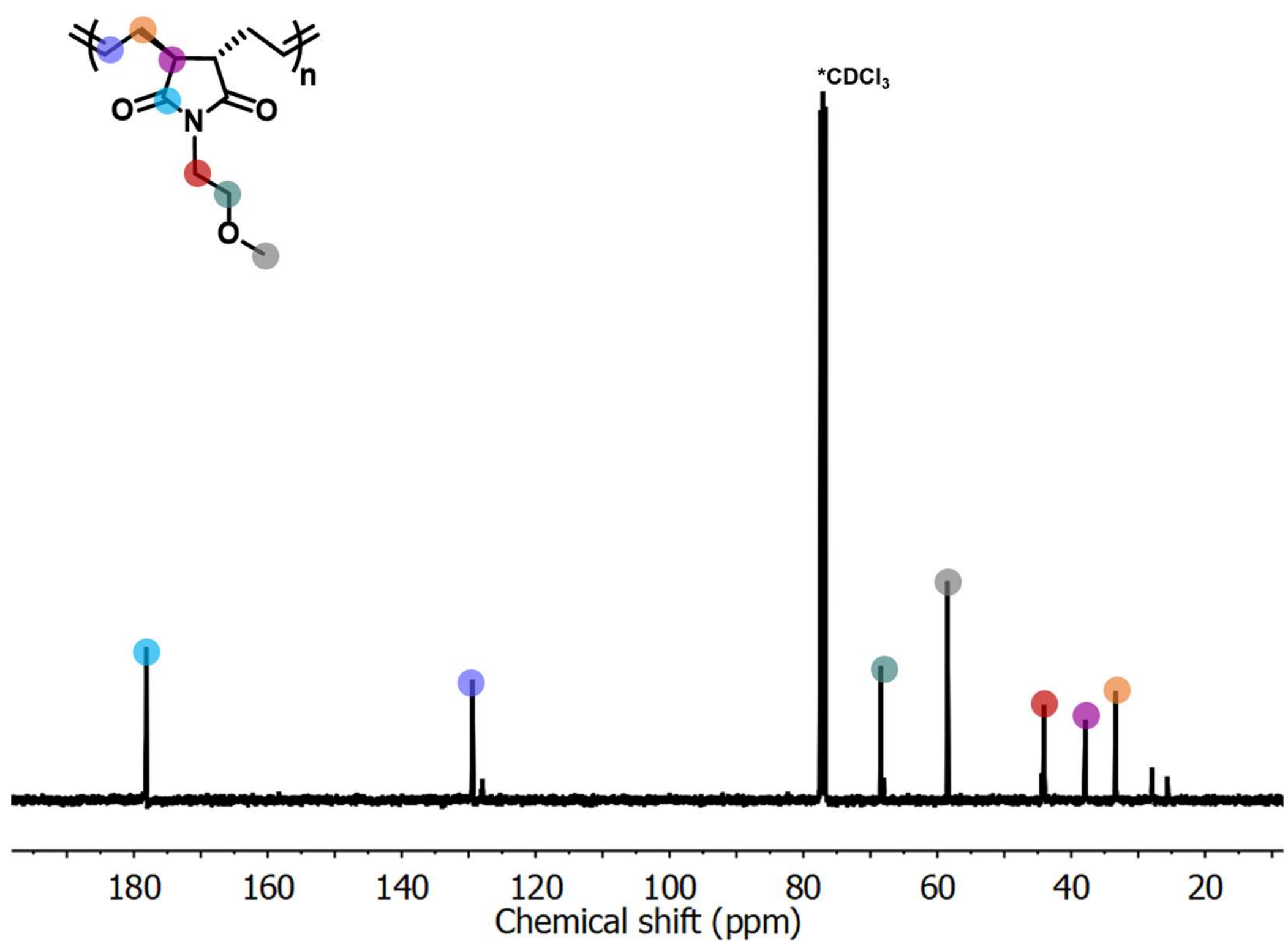

Figure S22. ^{13}C NMR spectrum of P3 in $CDCl_3$.

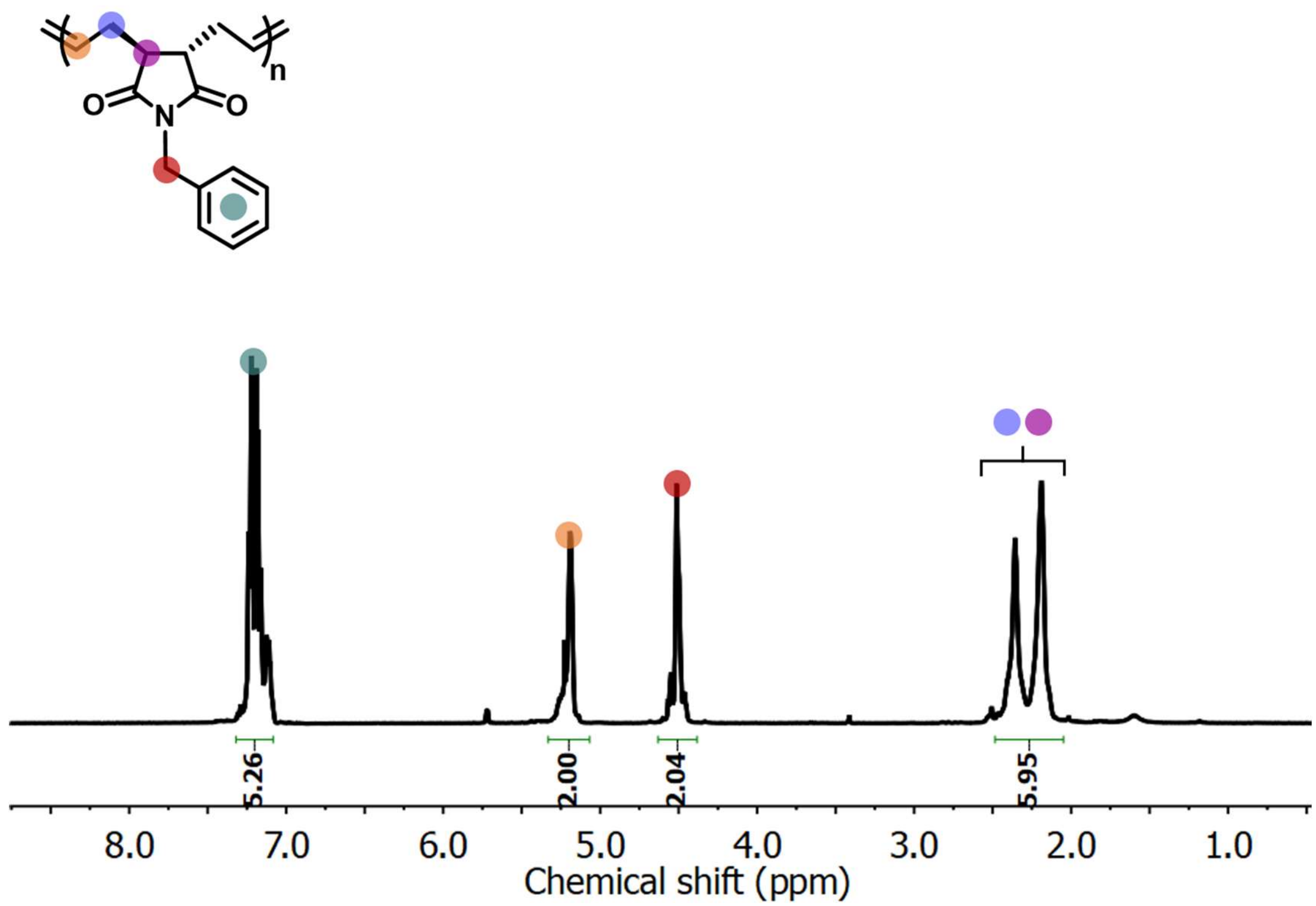

Figure S23. ^{1}H NMR spectrum of P4 in $CDCl_3$.

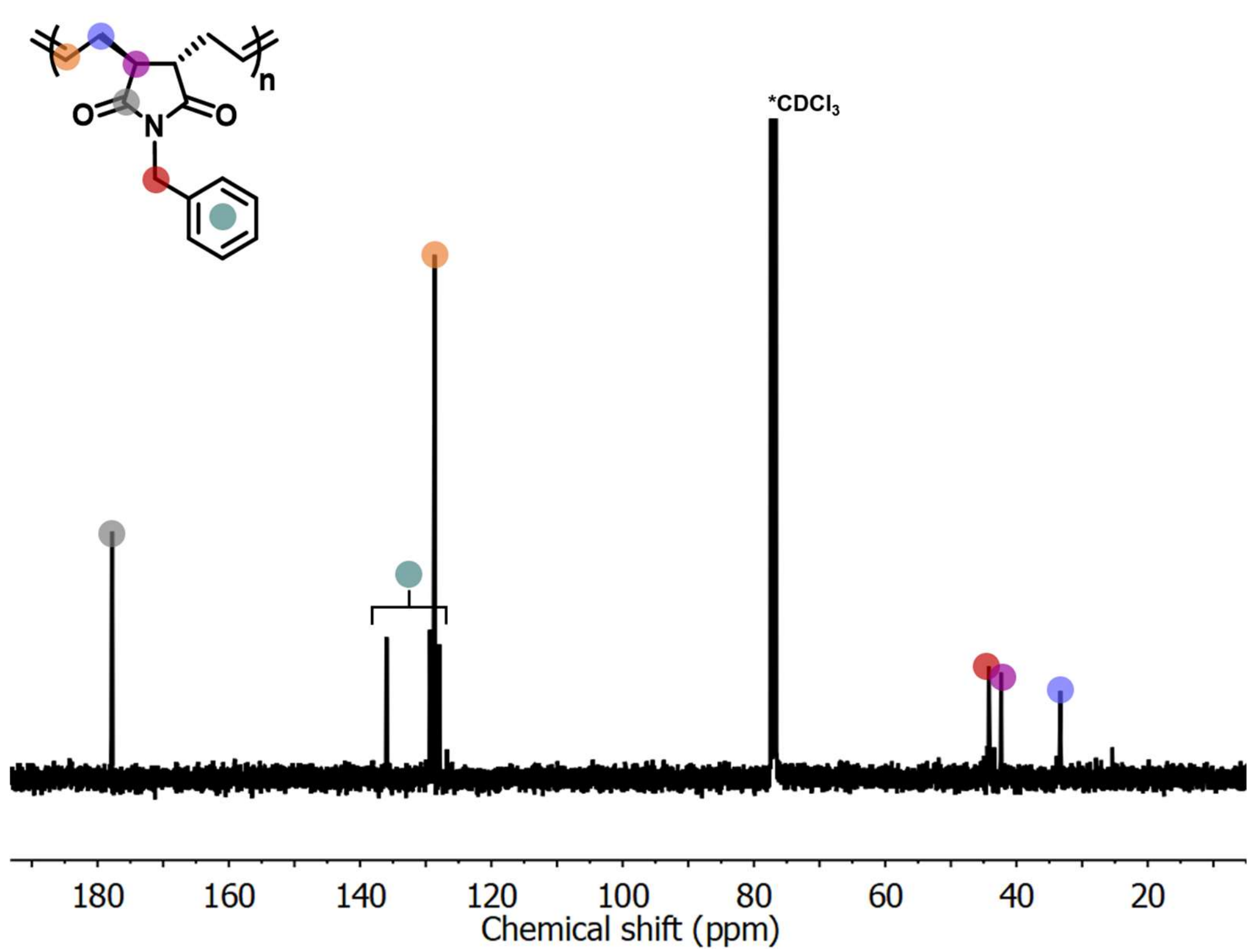

Figure S24. ^{13}C NMR spectrum of P4 in $CDCl_3$.

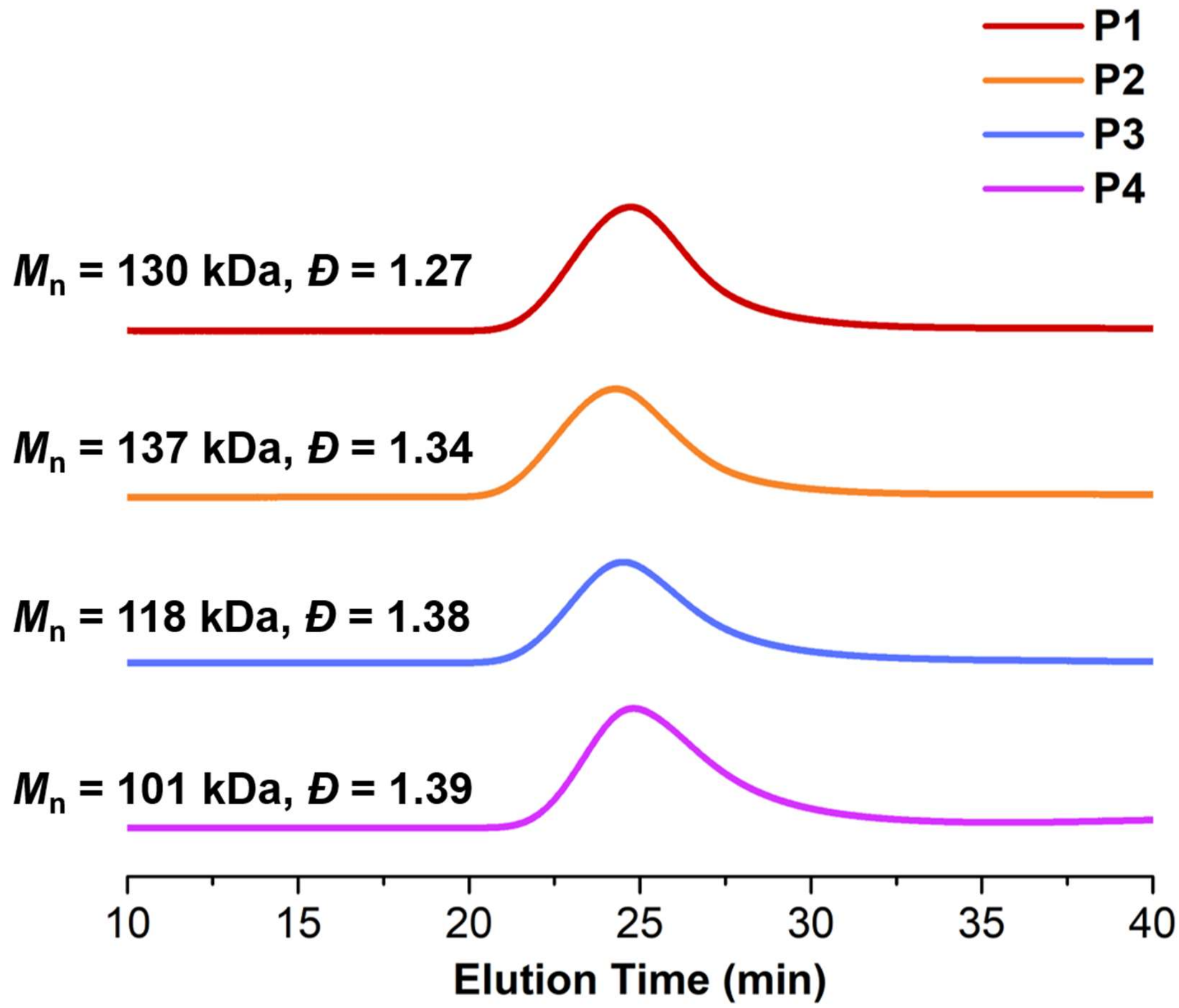

Figure S25. Size exclusion chromatography traces of P1–P4.

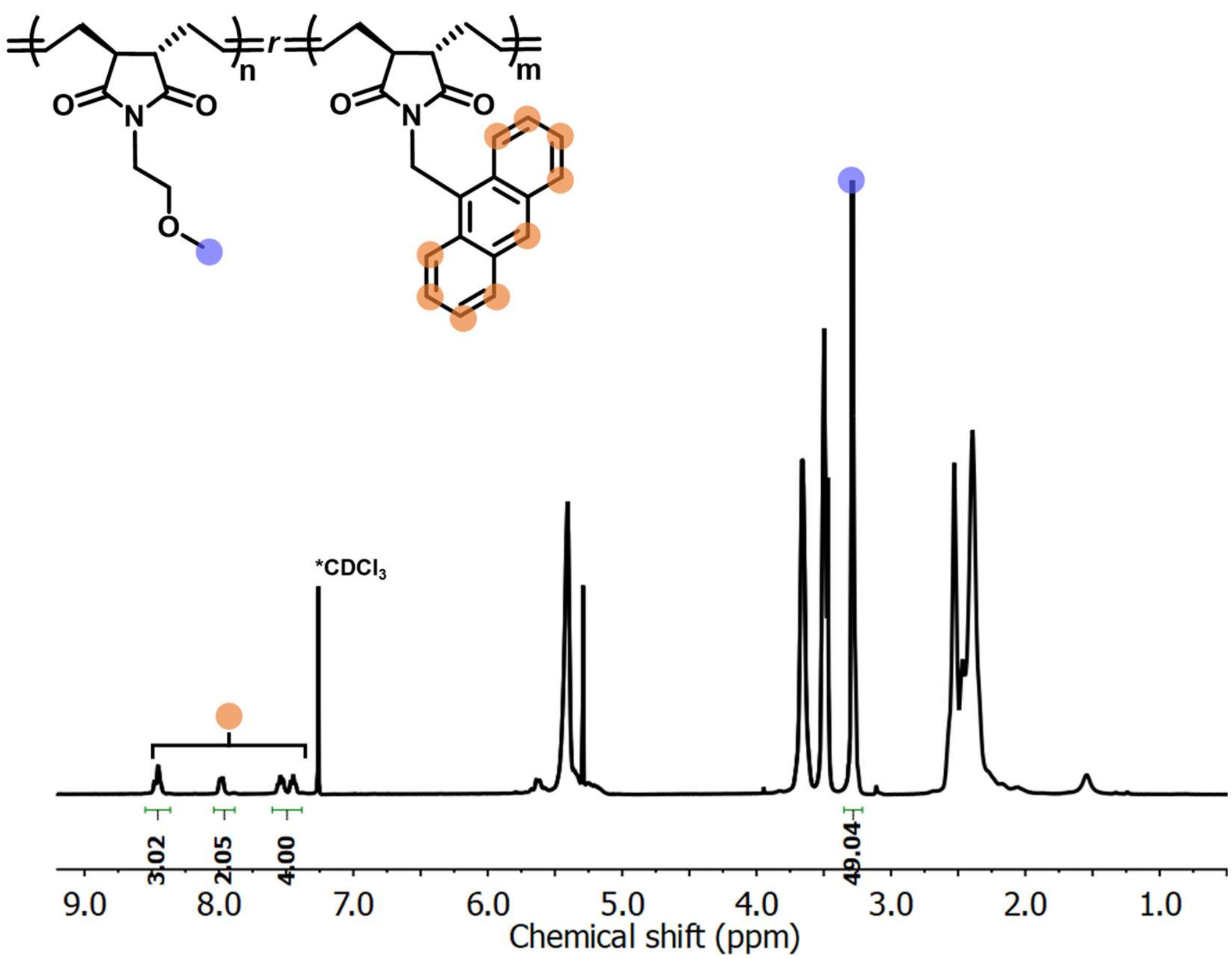

Figure S26. ^{1}H NMR spectrum of P3-*r*-P5 in $CDCl_3$. The feed ratio of M3 to M5 was 95:5.

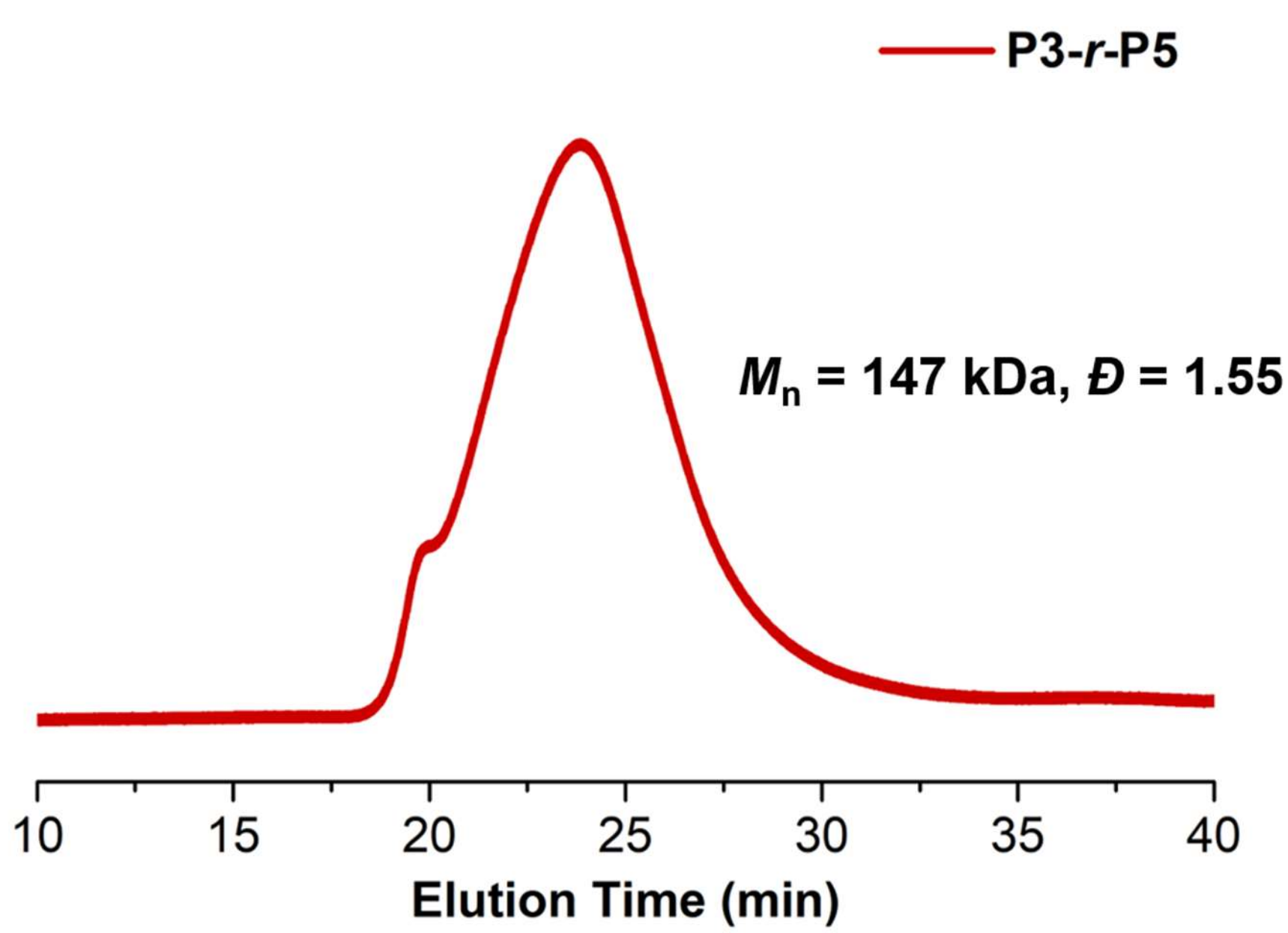

Figure S27. Size exclusion chromatography trace of P3-*r*-P5. The feed ratio of M3 to M5 was 95:5.

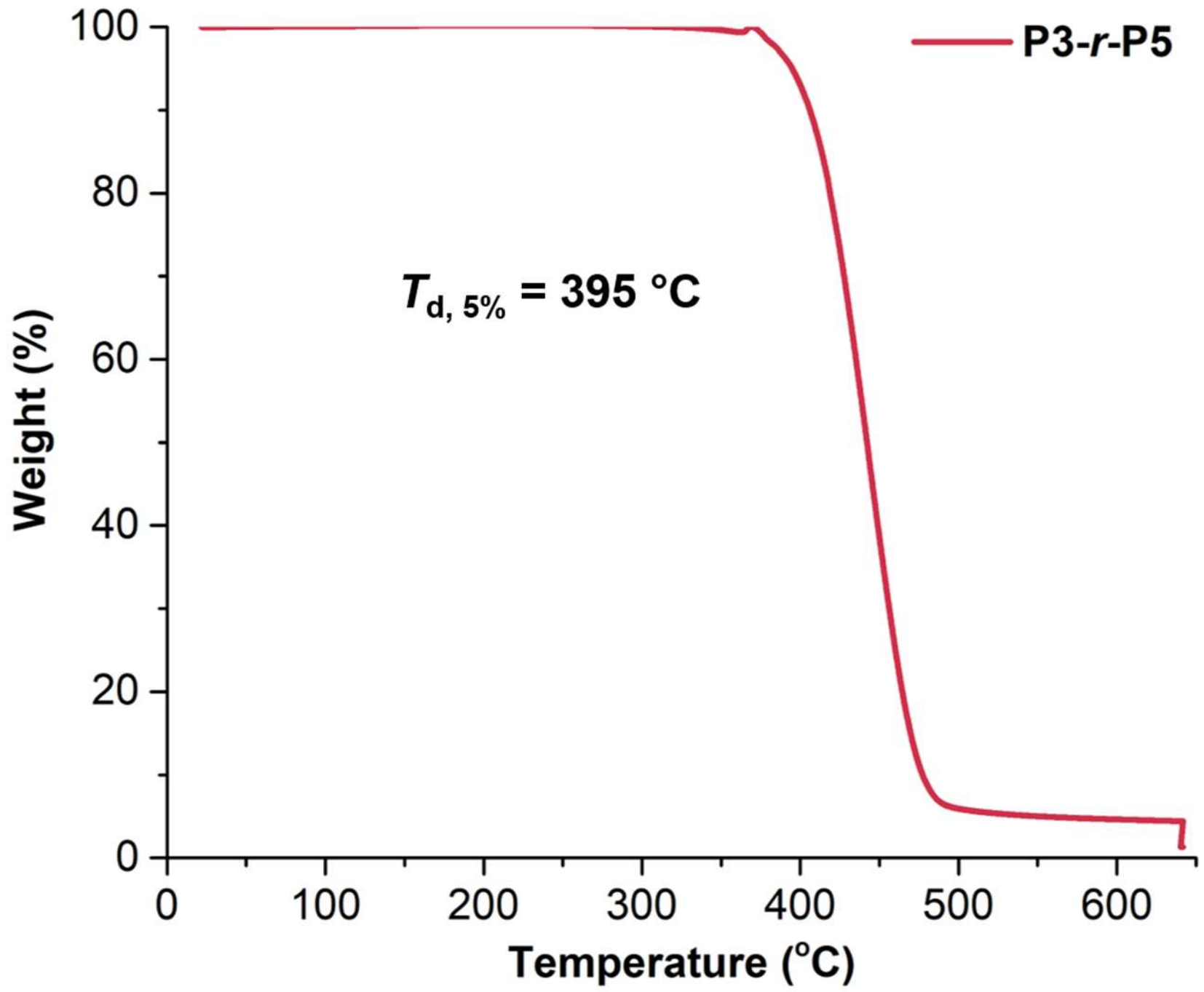

Figure S28. Thermogravimetric analysis of P3-*r*-P5. The feed ratio of M3 to M5 was 95:5.

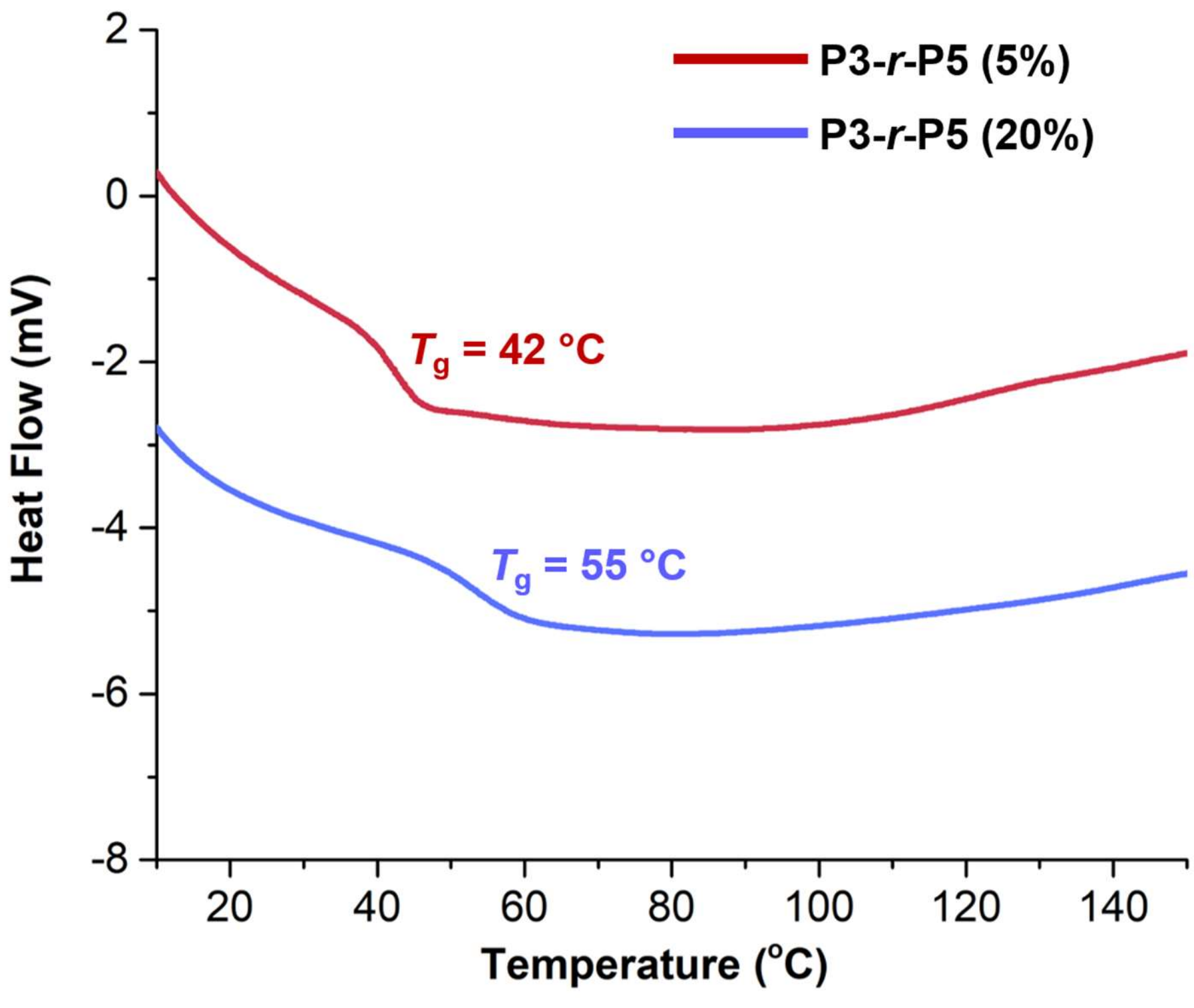

Figure S29. Differential scanning calorimetry thermograms of P3-*r*-P5 copolymers. The percentage of M5 in the feed is indicated in parentheses.

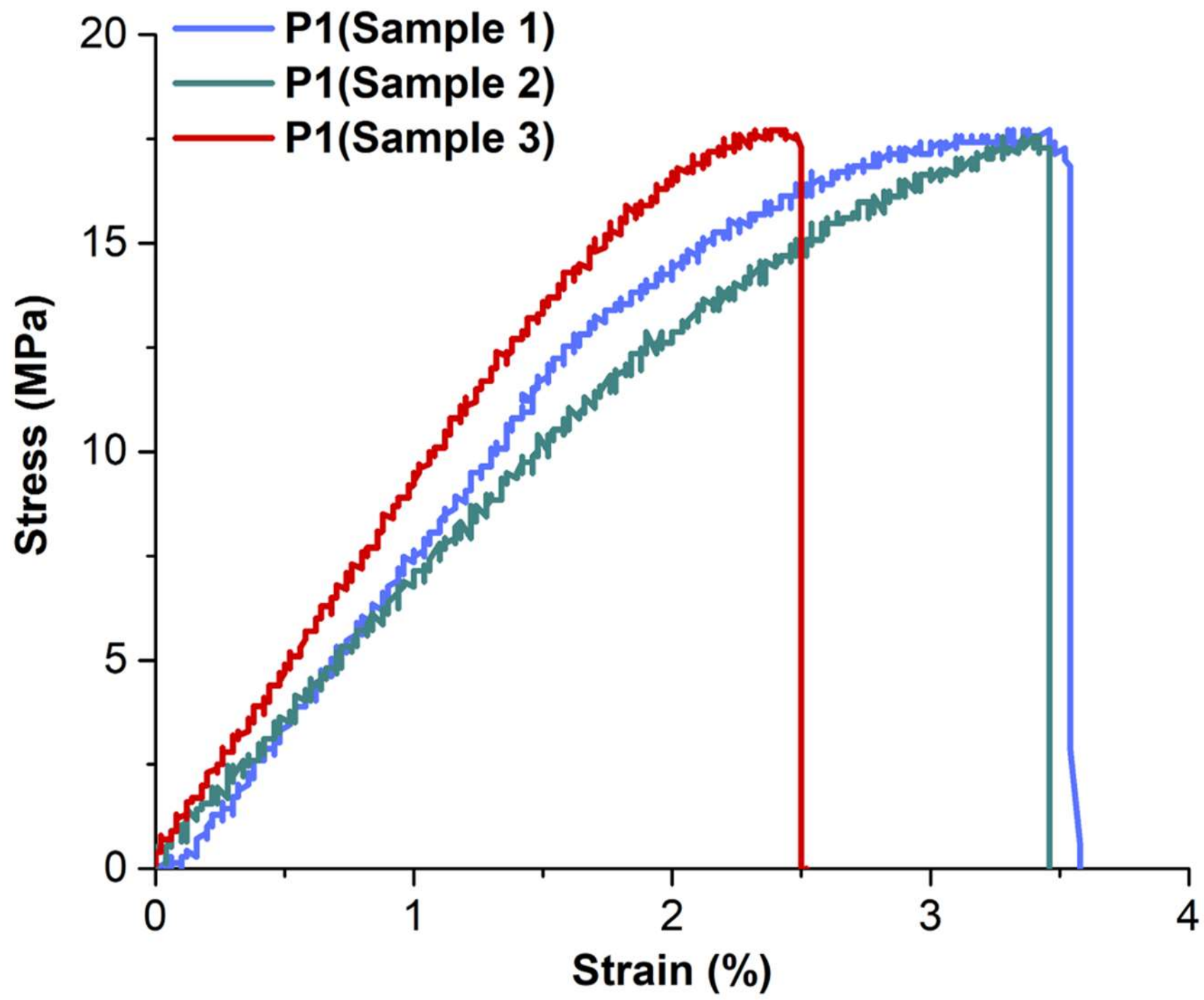

Figure S30. Stress‑strain curves obtained for the P1 samples.

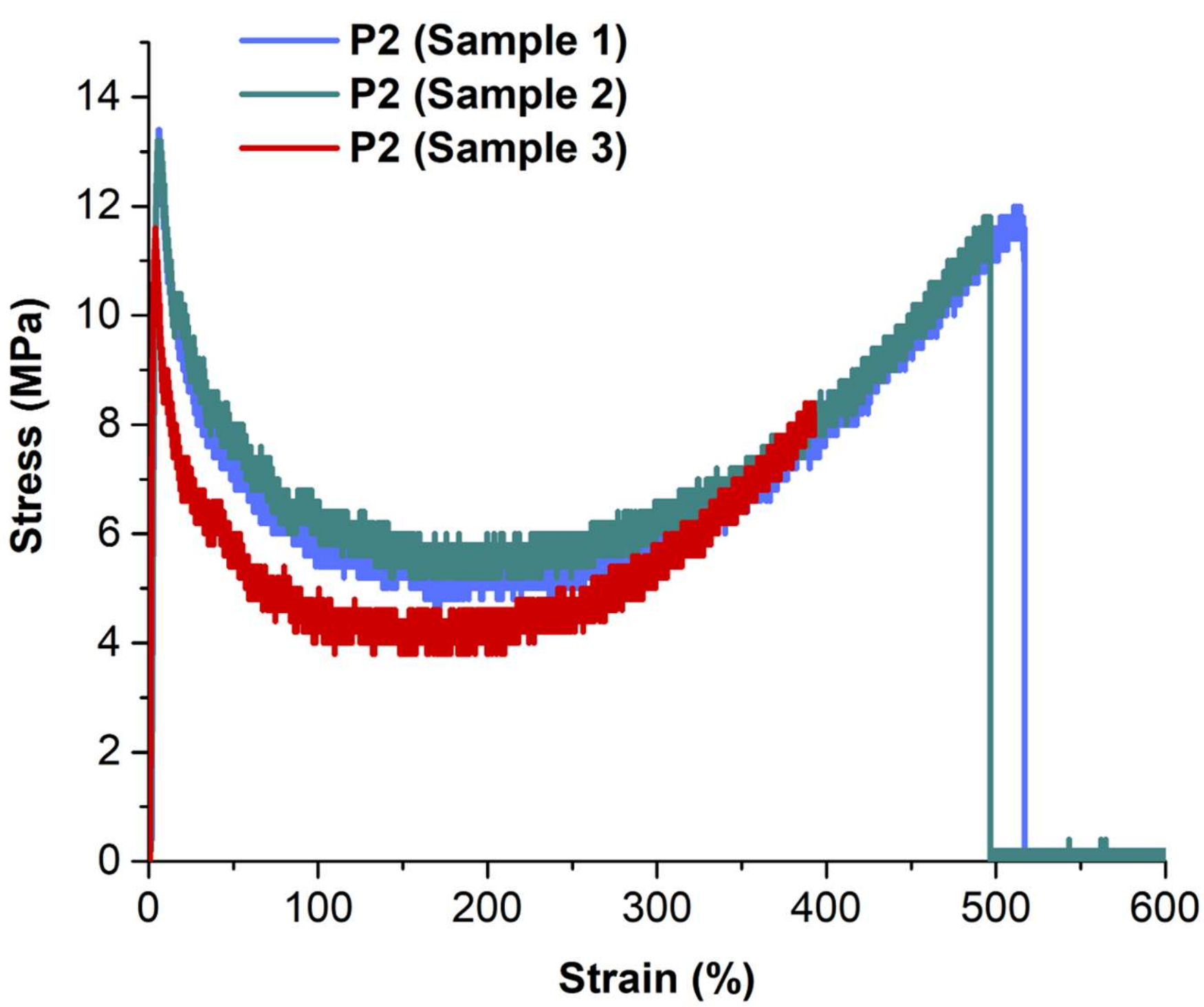

Figure S31. Stress‑strain curves obtained for the P2 samples.

Table S1. ROMP of M1 at various temperatures (initial concentration = 0.5 M)

Entry	M1/G3	Temperature (K)	1/T (K^{-1})	Time (h)	Conv. (%)	$[M]_{eq}$ (M)	$ln[M]_{eq}$
1	100	273.15	0.003661	12	47.0	0.265	-1.32803
2	100	283.15	0.003532	12	39.0	0.305	-1.18744
3	100	293.15	0.003411	12	33.0	0.335	-1.09362
4	100	303.15	0.003299	12	28.4	0.358	-1.02722
5	100	313.15	0.003193	12	19.0	0.405	-0.90387

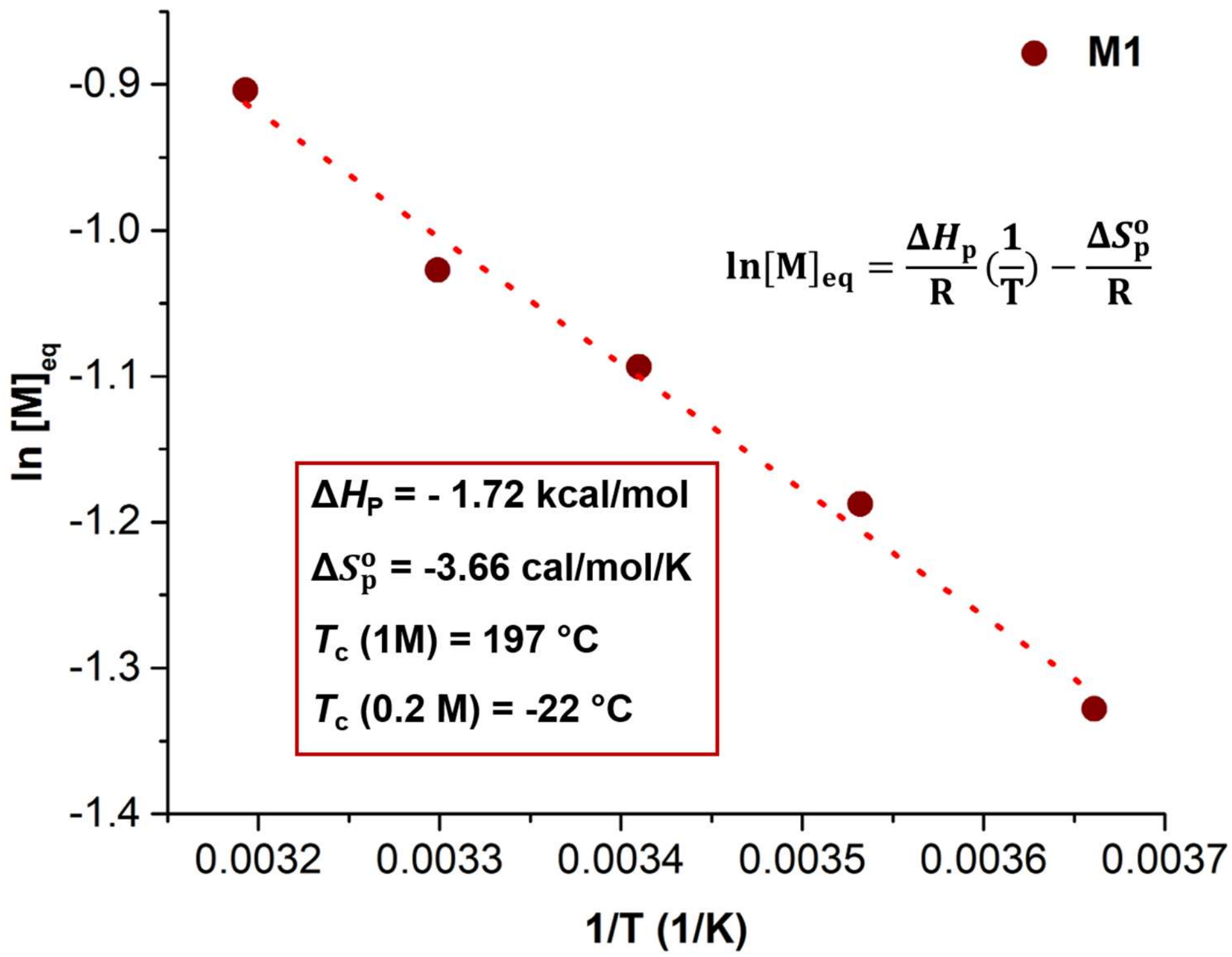

Figure S32. Van 't Hoff plot of M1. The enthalpy change and standard-state entropy change of polymerization, as well as the ceiling temperatures at different monomer concentrations, were determined according to the methods described in Section 4.10.

Table S2. ROMP of M4 at various temperatures (initial concentration = 0.5 M)

Entry	M1/G3	Temperature (K)	1/T (K^{-1})	Time (h)	Conv. (%)	$[M]_{eq}$ (M)	$ln[M]_{eq}$
1	100	273.15	0.003661	12	49.0	0.255	-1.3665
2	100	283.15	0.003532	12	39.4	0.303	-1.1940
3	100	293.15	0.003411	12	31.0	0.345	-1.0642
4	100	303.15	0.003299	12	27.4	0.363	-1.0133

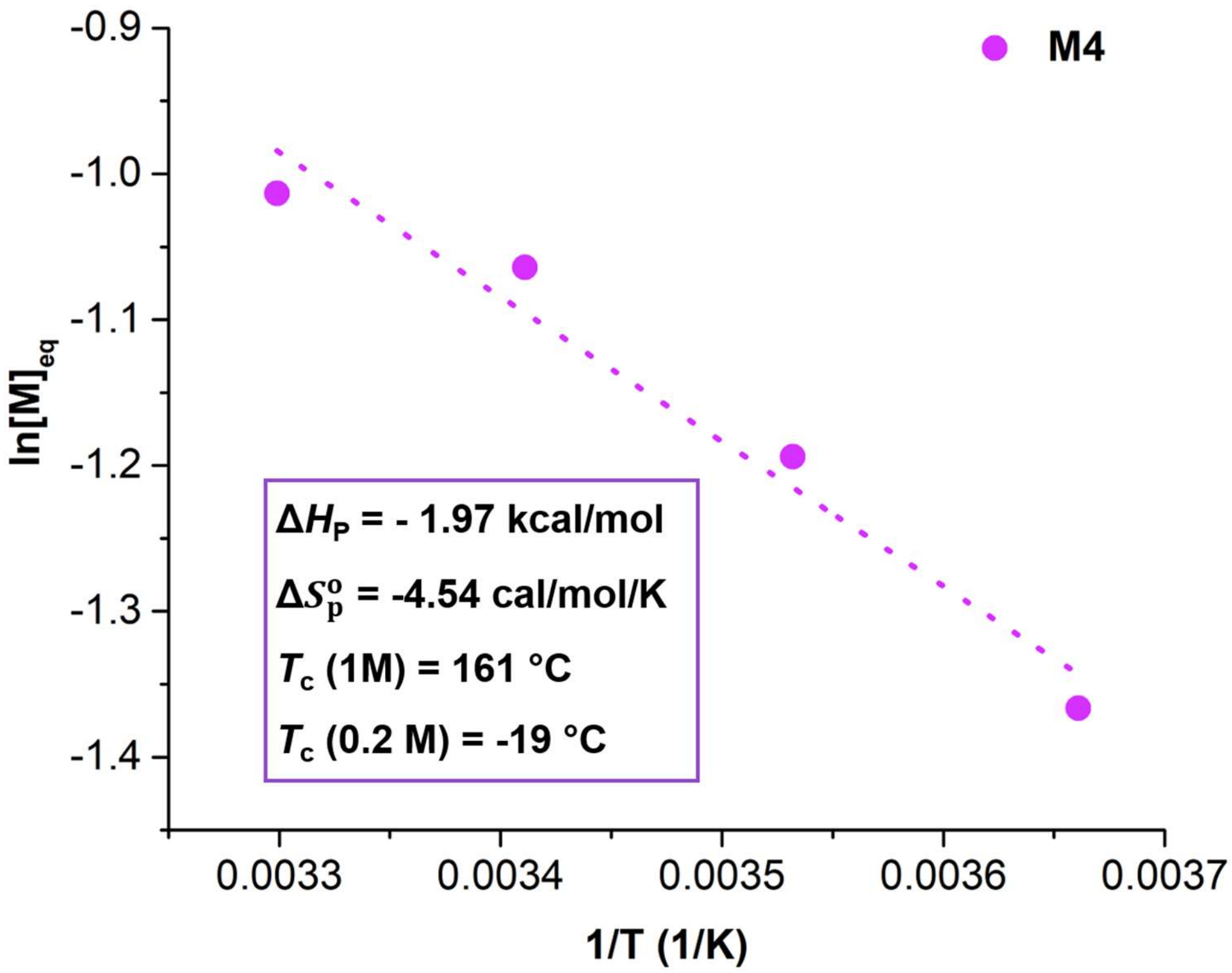

Figure S33. Van 't Hoff plot of M4. The enthalpy change and standard-state entropy change of polymerization, as well as the ceiling temperatures at different monomer concentrations, were determined according to the methods described in Section 4.10.

Table S3. Optimization of depolymerization conditions of P4

Entry	[olefin]$_0$	G2 Loading	Temperature (°C)	Time (h)	Conv. (%)
1	20 mM	2 mol%	35	12	>95
2	20 mM	2 mol%	20	12	>95
3	50 mM	2 mol%	20	12	>95
4	100 mM	2 mol%	20	12	>95
5	200 mM	2 mol%	20	12	>95
6	200 mM	1 mol%	20	12	93

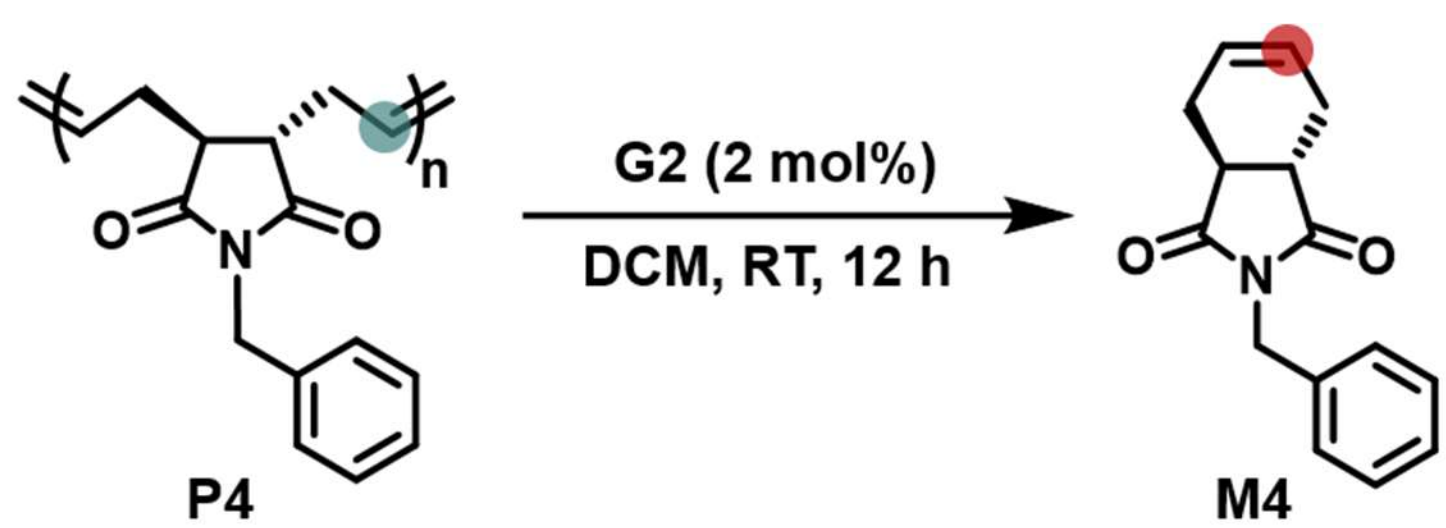

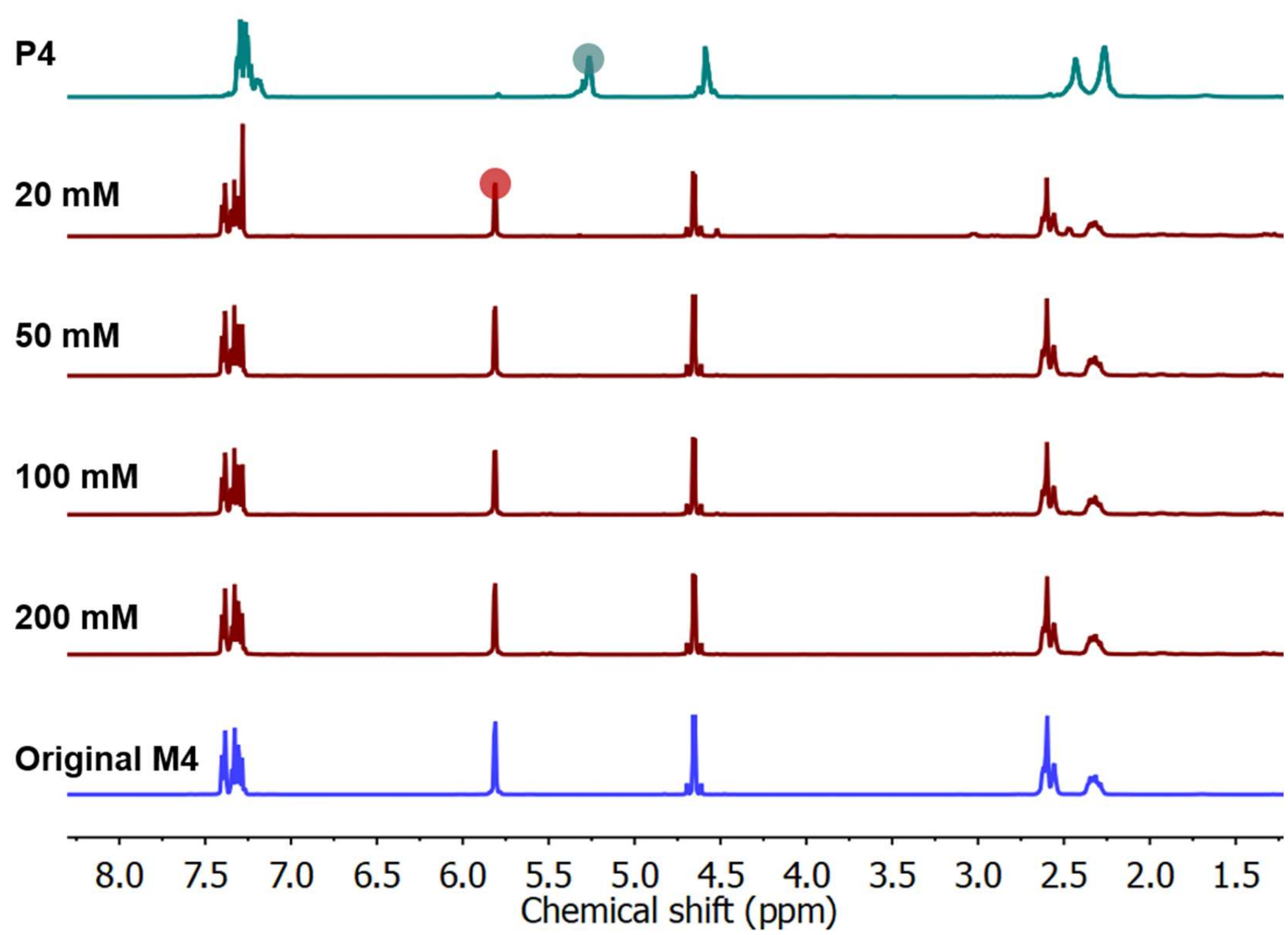

Figure S34. ^{1}H NMR spectra of of P4 (green), M4 (blue), and depolymerized products obtained at different concentrations (red).

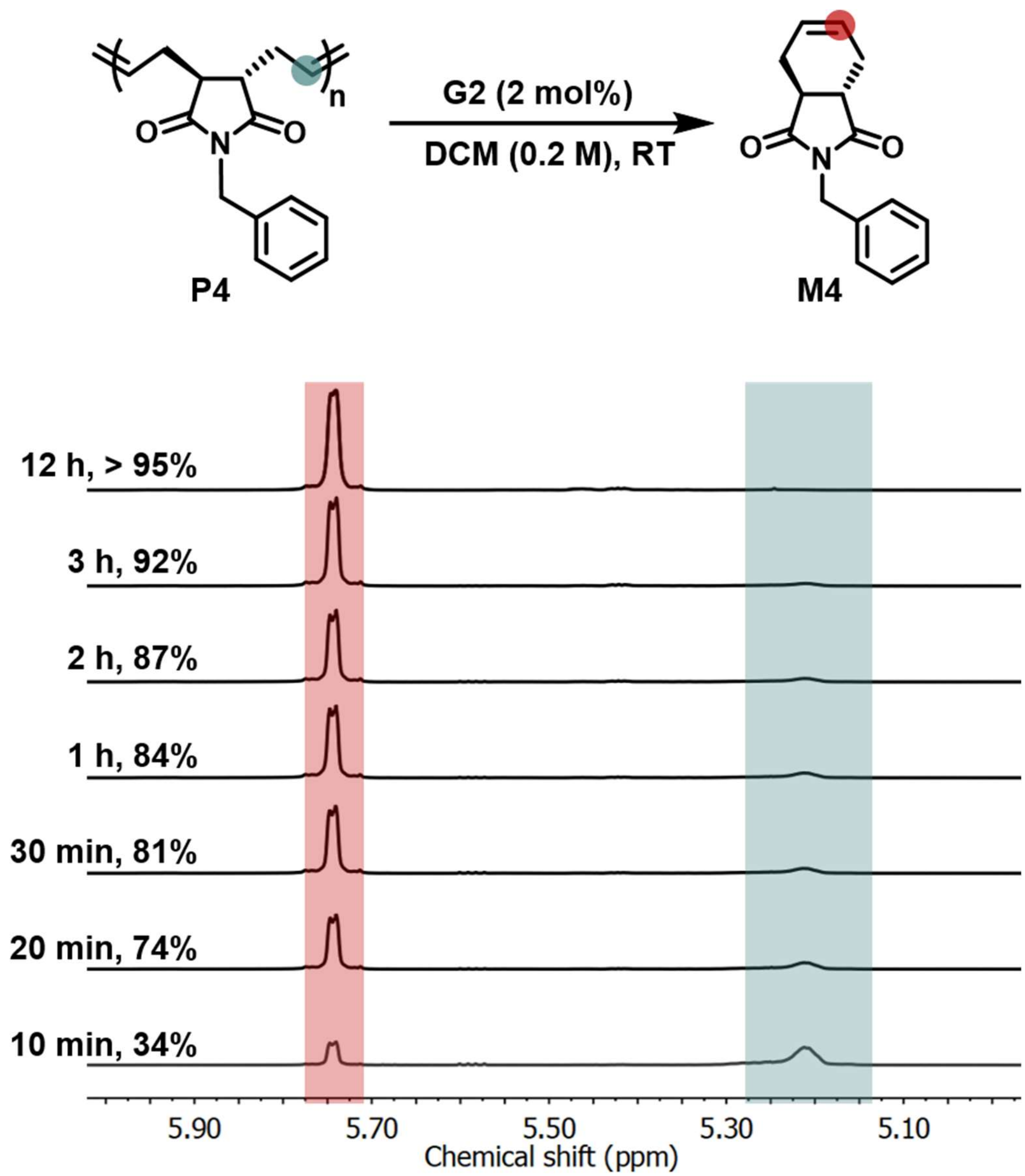

Figure S35. Partial ^{1}H NMR spectra for the depolymerization kinetics of P4 (0.2 M) in DCM at 20 °C.

Table S4. Depolymerization study of P1-P4 at optimized conditions

Entry	$[olefin]_0$	G2 Loading	Temperature (°C)	Time (h)	Conv. (%)
**P1**	200 mM	2 mol%	20	12	>95
**P2**	200 mM	2 mol%	20	12	>95
**P3**	200 mM	2 mol%	20	12	>95
**P4**	200 mM	2 mol%	20	12	>95

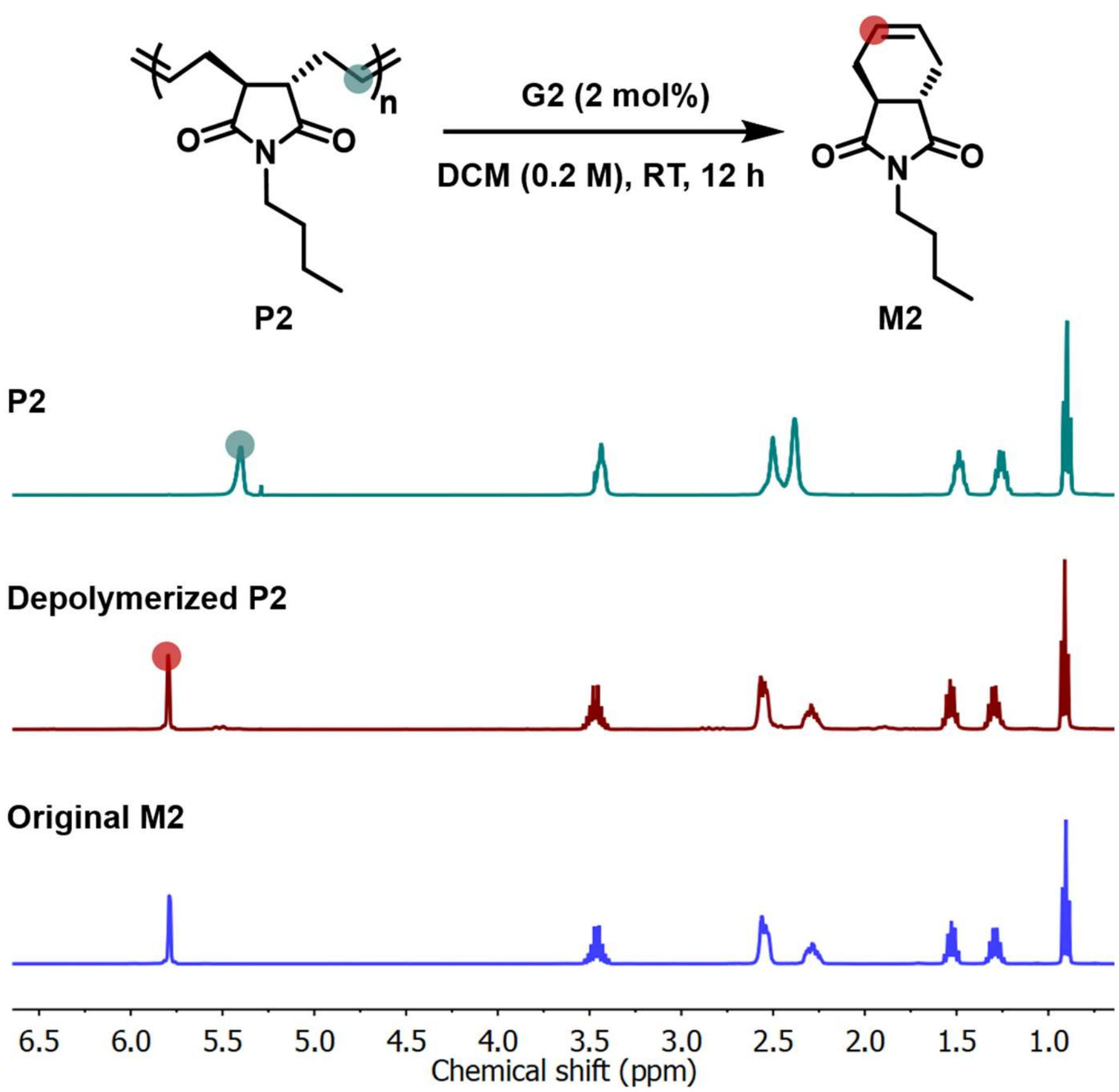

Figure S36. ^{1}H NMR spectra of of P2 (green), depolymerized product (red), and M2 (blue).

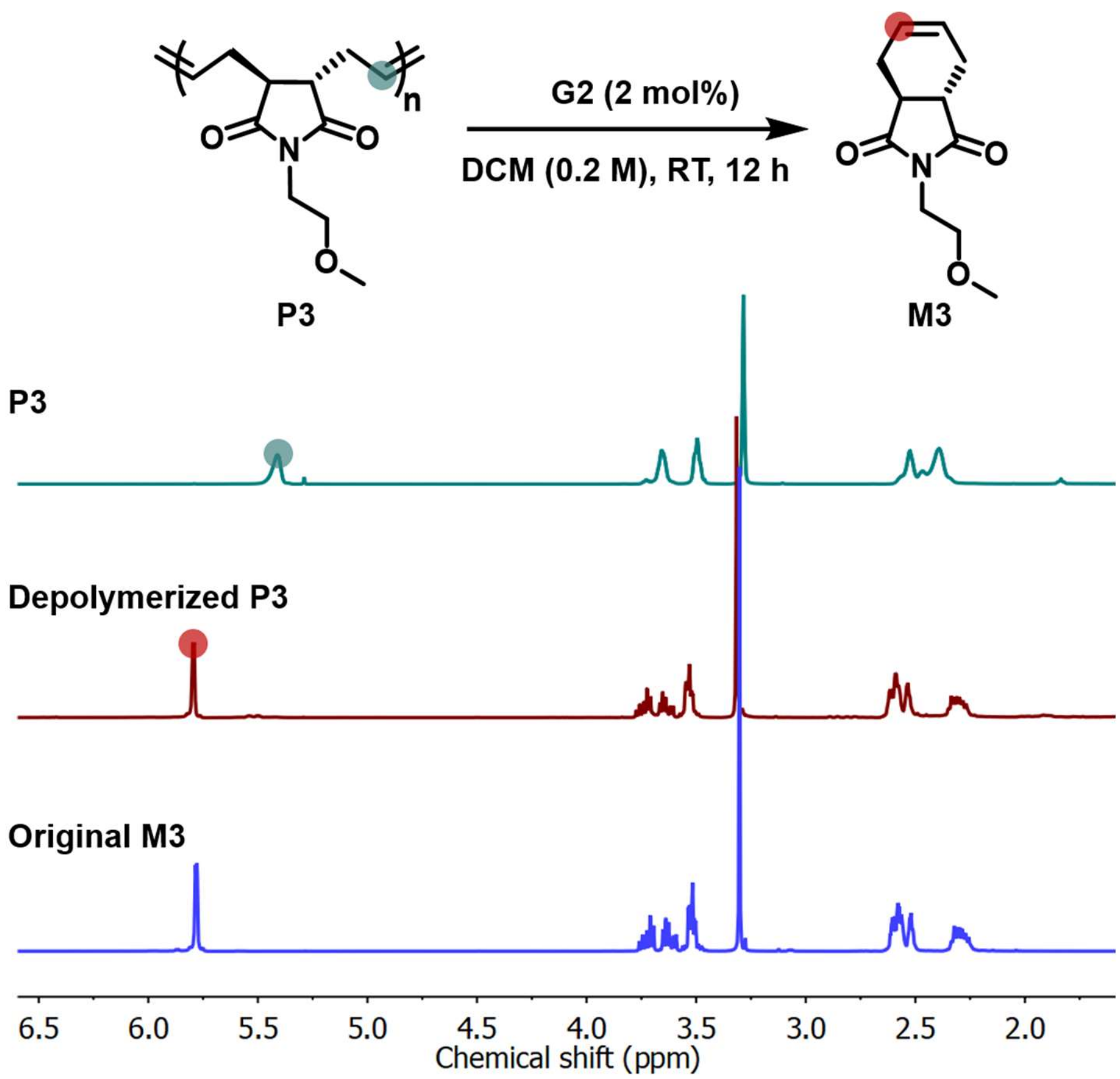

Figure S37. ^{1}H NMR spectra of of P3 (green), depolymerized product (red), and M3 (blue).

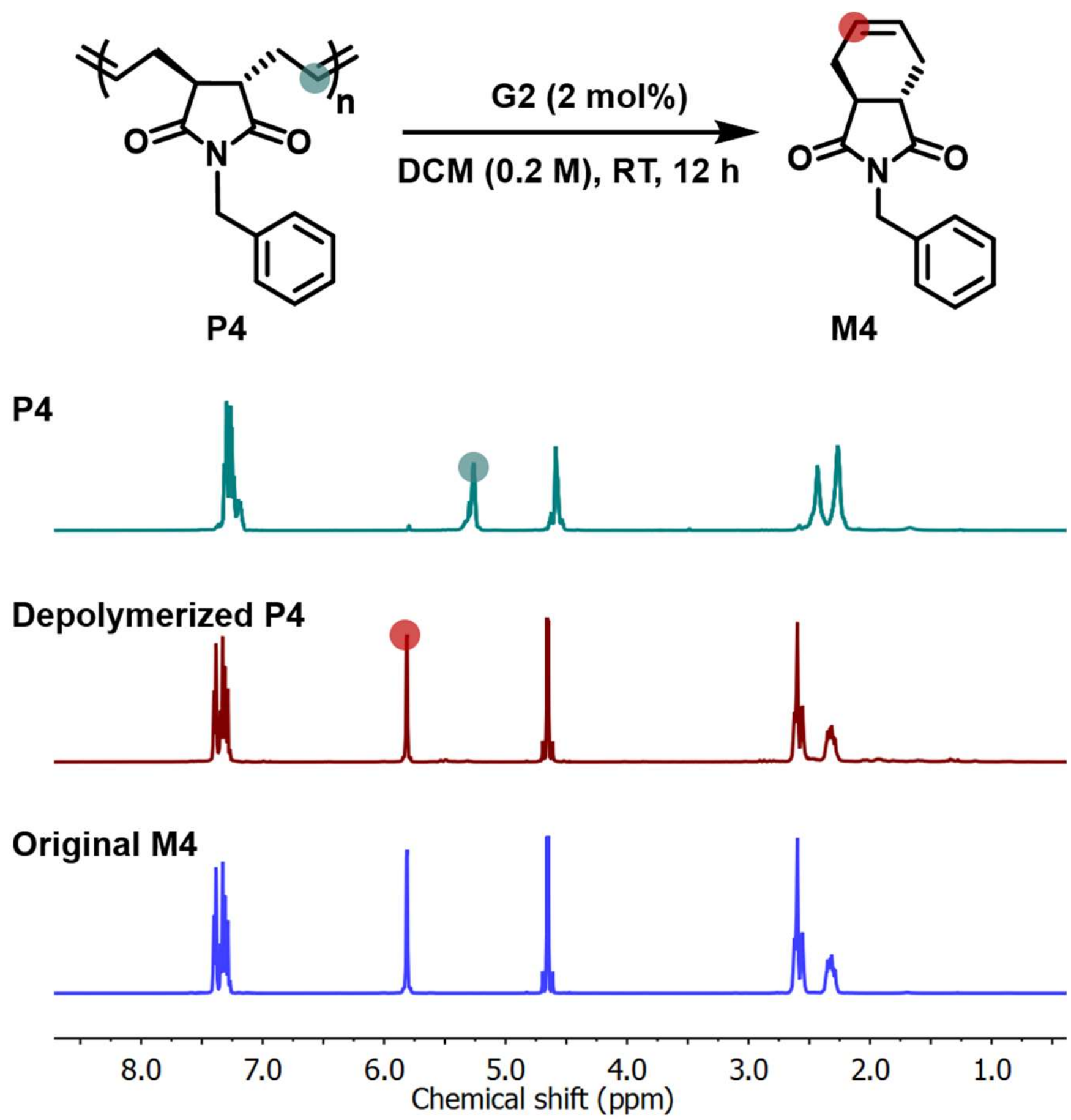

Figure S38. ^{1}H NMR spectra of of P4 (green), depolymerized product (red), and M4 (blue).

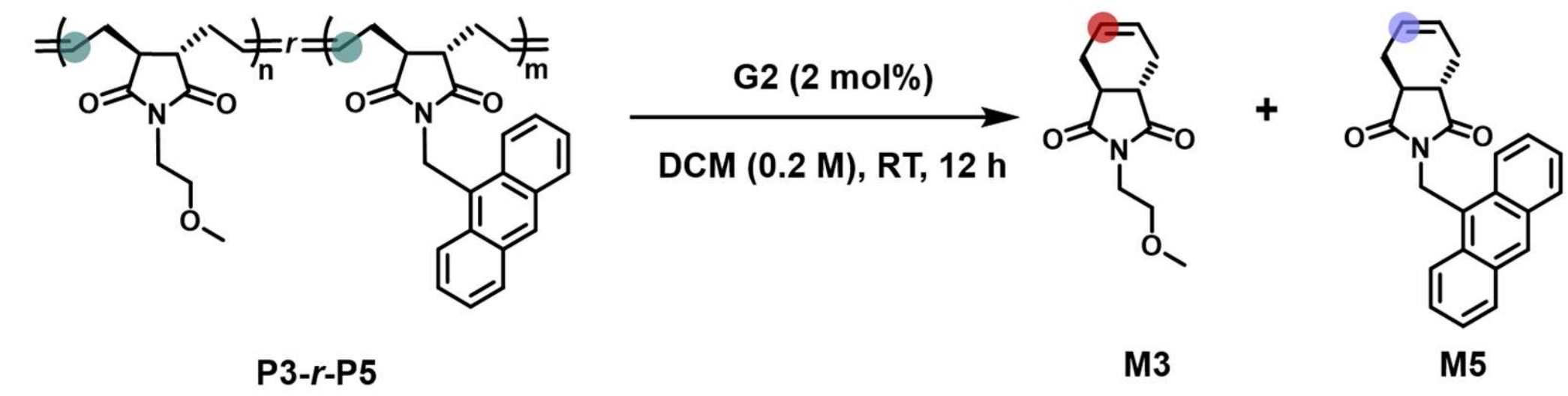

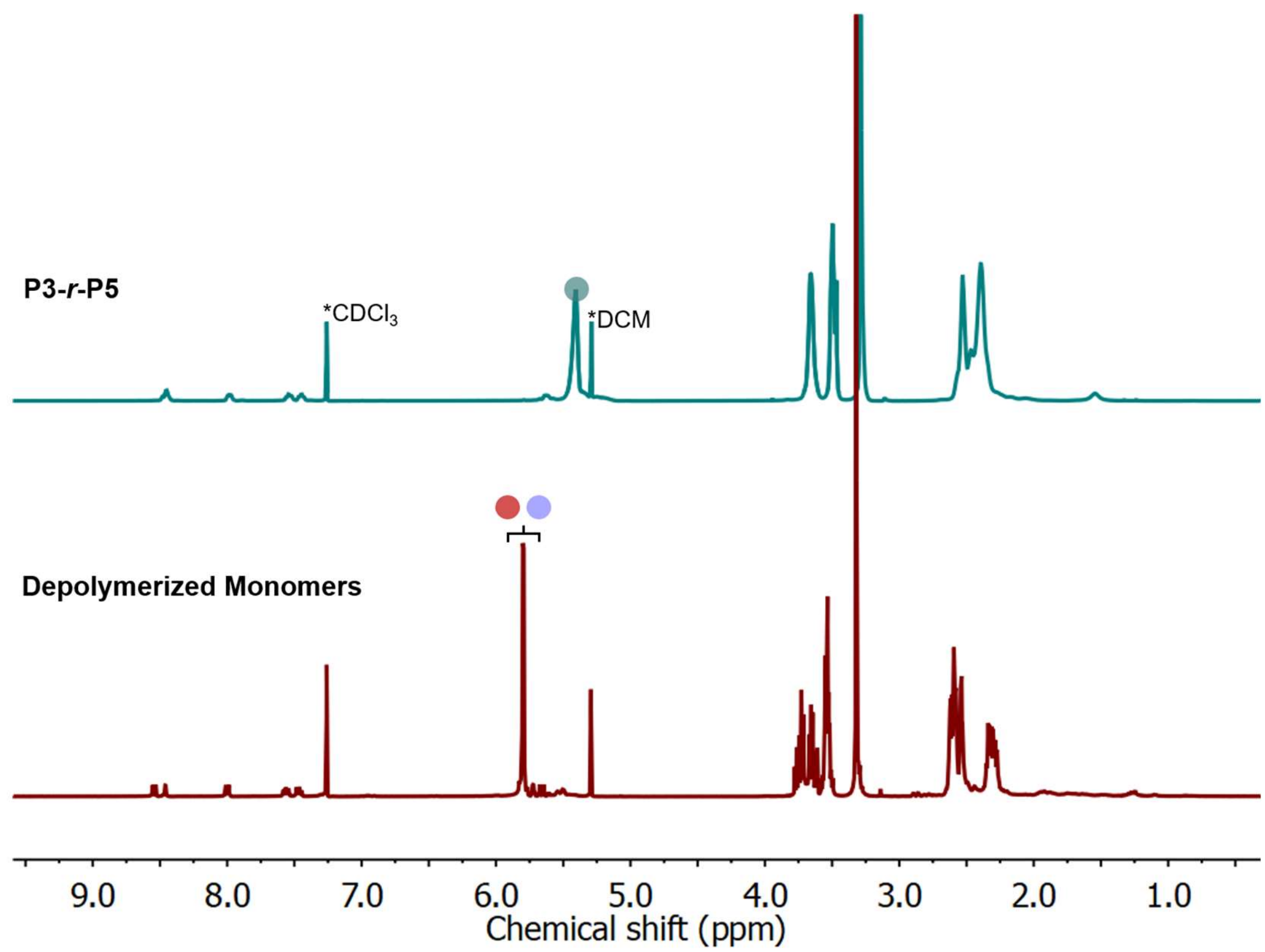

Figure S39. ^{1}H NMR spectra of of copolymer P3-*r*-P5 (green) and depolymerized product (red).

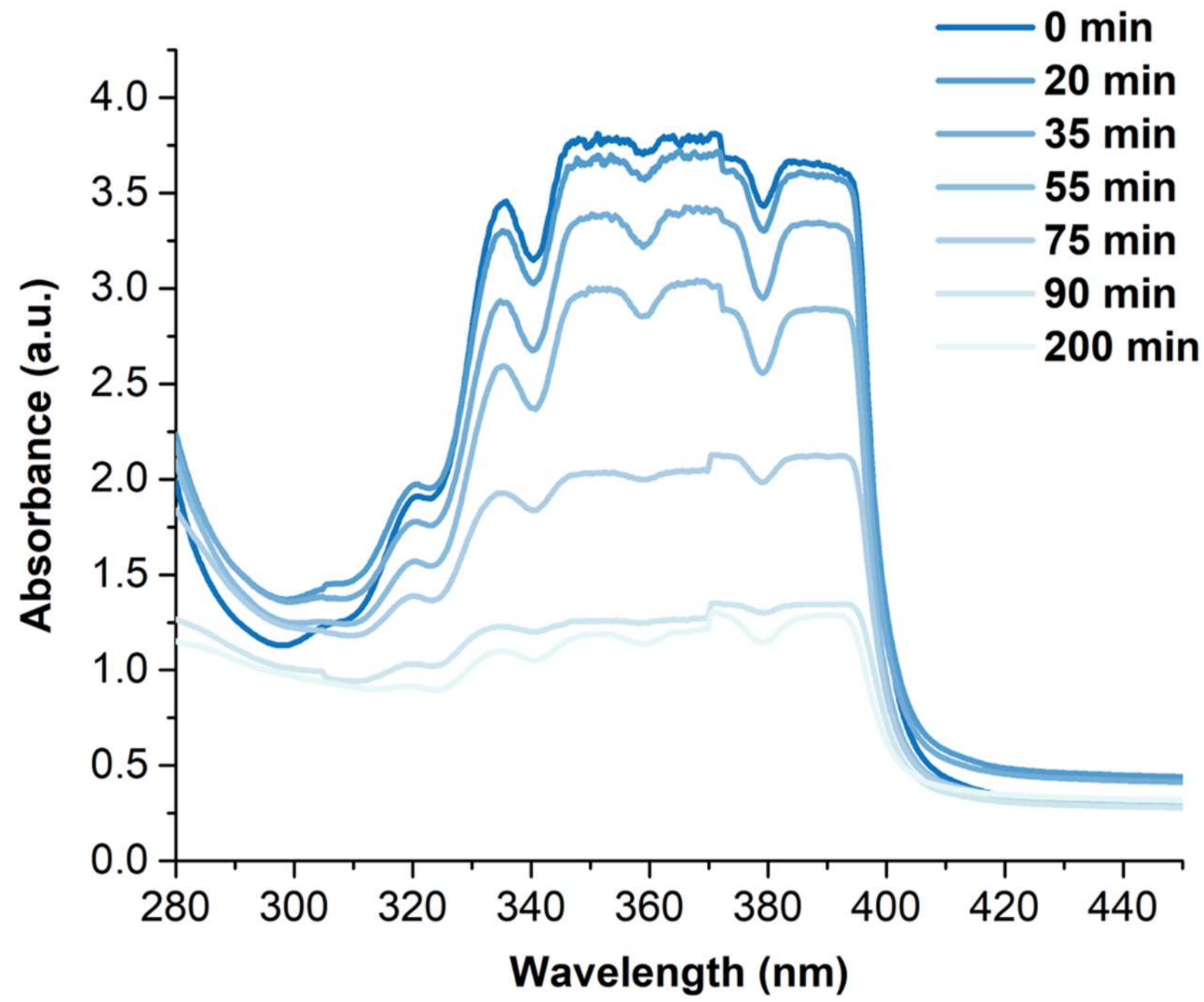

Figure S40. UV–Vis spectra of a P3-*r*-P5 thin film as a function of irradiation time under 365 nm UV light.

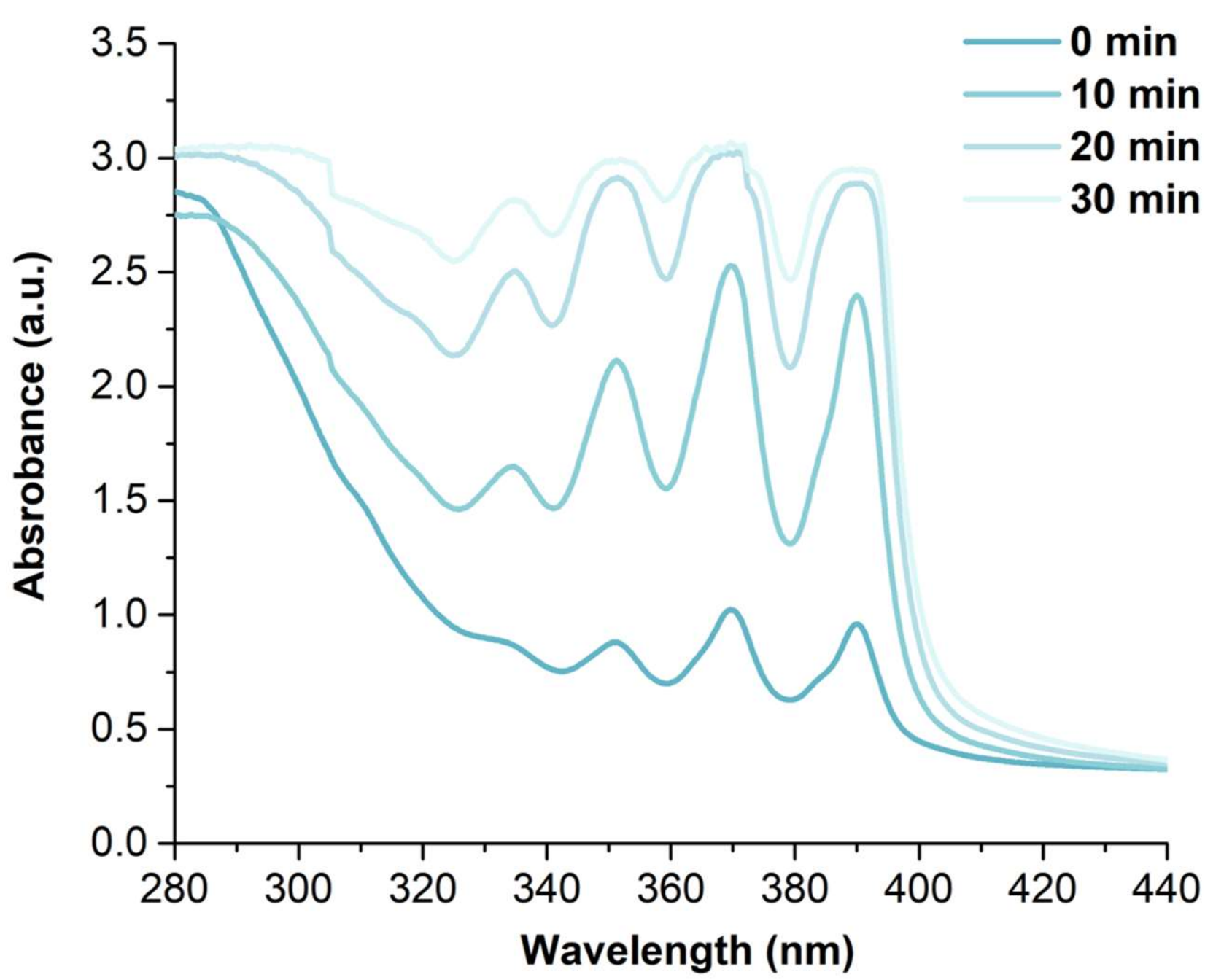

Figure S41. UV–Vis spectra of a crosslinked P3-*r*-P5 thin film as a function of heating time at 110 °C.

Table S5. Calculated bond angles of *t*SICH, *c*SICH, their ring-opened structures, and succinimide. The total difference was calculated as the sum of the individual bond angle differences.

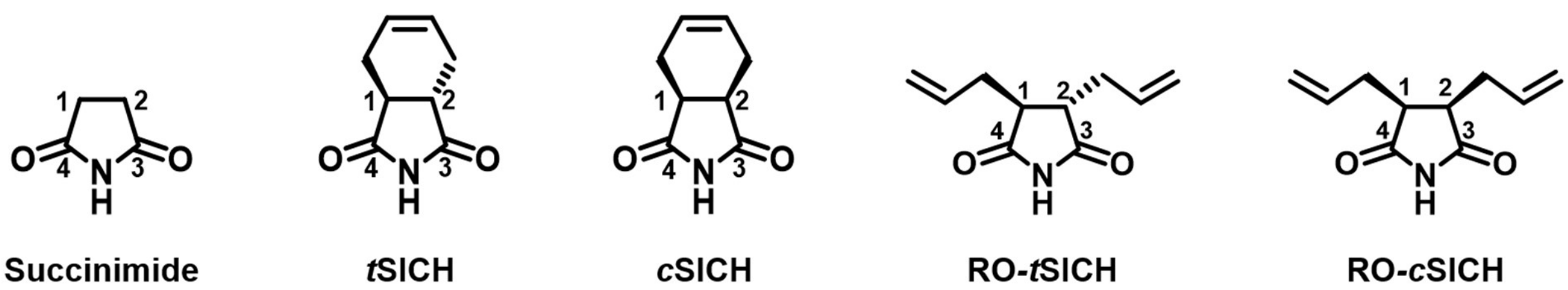

Bond Angles (°)	C4-C1-C2	C1-C2-C3	C2-C3-C4	C3-N-C4	N-C4-C1	Total Difference (°)
**Succinimide**	104.31	104.31	109.01	113.35	109.01	N/A
***t*SICH**	103.43	103.43	104.92	114.75	104.92	11.34
***c*SICH**	105.22	105.22	107.20	115.15	107.20	7.24
**RO-*t*SICH**	104.93	104.86	107.24	114.81	107.23	6.18
**RO-*c*SICH**	104.53	103.70	107.19	114.86	106.76	6.41

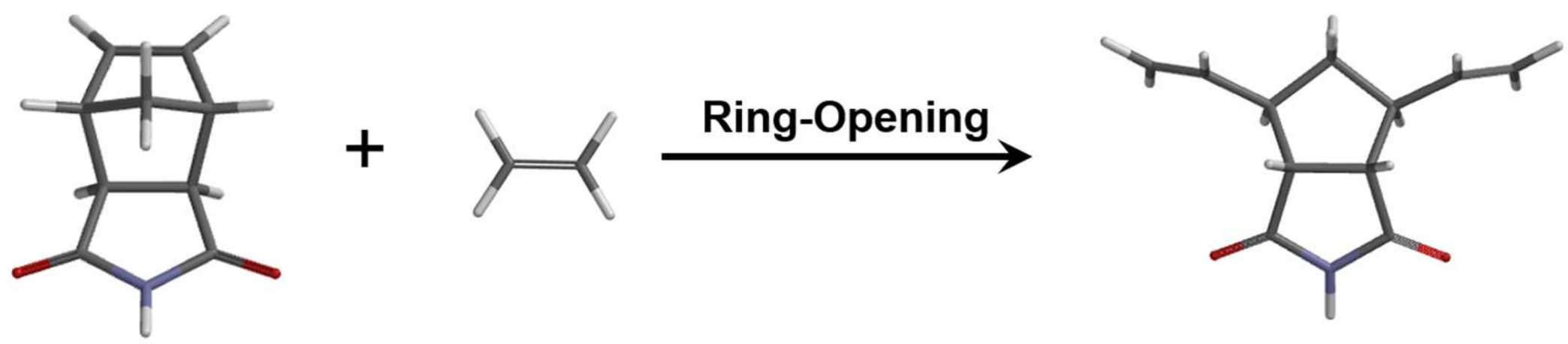

Chemical Structure	Enthalpy (Hartree)
Exo-NBE Imide	-553.391105
Ethylene	-78.5322597
Ring-Opened Product	-631.947445

RSE = -ΔH = 631.947445 - 631.923365 = 0.02408 Hartree = 15.1 kcal/mol

Figure S42. DFT calculation of ethenolysis ring strain energy (RSE) of exo-norbornene imide. A B3LYP/6-31G* level of theory was applied for the geometry optimization and energy calculation of conformers in vacuum. The RSE was estimated as the enthalpy difference between the ring-opened product and the total enthalpy of the isolated reactants (Exo-NBE Imide + ethylene), using their lowest-energy conformers.

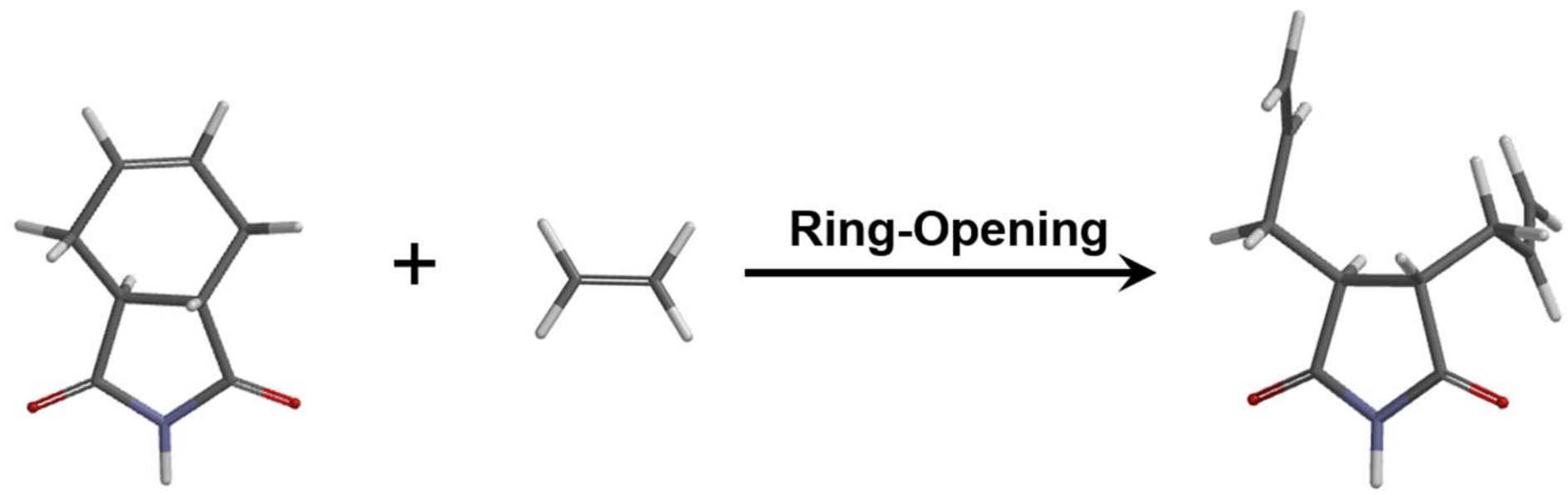

Chemical Structure	Enthalpy (Hartree)
*t*SICH	-515.310044
Ethylene	-78.5322597
Ring-Opened Product	-593.851684

RSE = -ΔH = 593.851684 - 593.842304 = 0.009380 Hartree = 5.9 kcal/mol

Figure S43. DFT calculation of ethenolysis ring strain energy (RSE) of *t*SICH. A B3LYP/6-31G* level of theory was applied for the geometry optimization and energy calculation of conformers in vacuum. The RSE was estimated as the enthalpy difference between the ring-opened product and the total enthalpy of the isolated reactants (*t*SICH + ethylene), using their lowest-energy conformers.

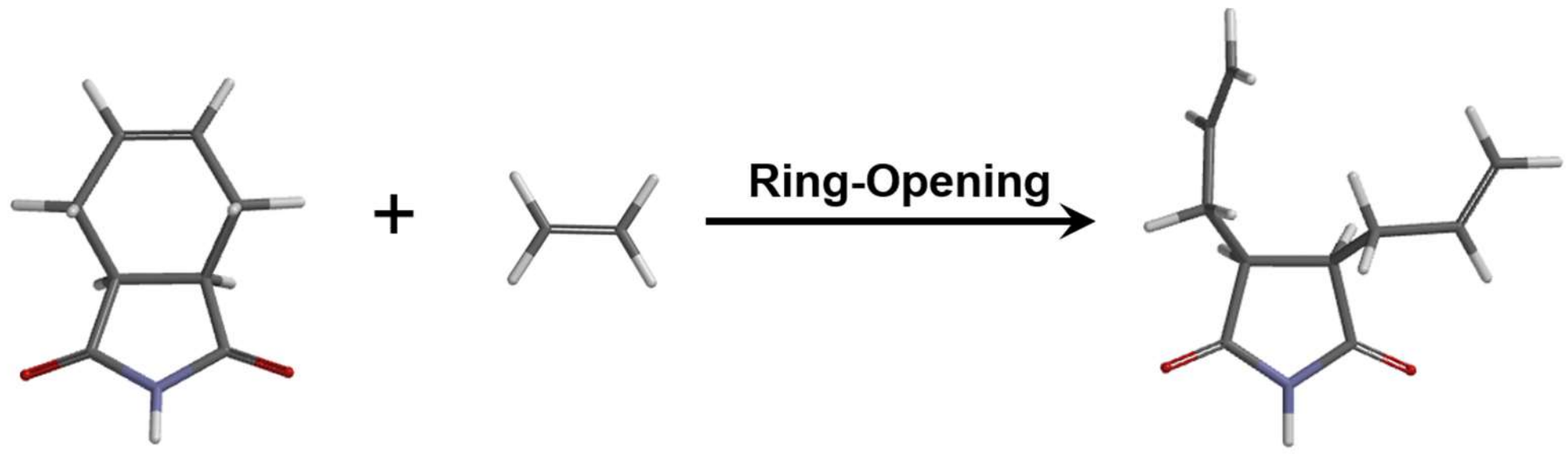

Chemical Structure	Enthalpy (Hartree)
*c*SICH	-515.311463
Ethylene	-78.5322597
Ring-Opened Product	-593.846484

RSE = -ΔH = 593.846484 - 593.843723 = 0.002761 Hartree = 1.7 kcal/mol

Figure S44. DFT calculation of ethenolysis ring strain energy (RSE) of *c*SICH. A B3LYP/6-31G* level of theory was applied for the geometry optimization and energy calculation of conformers in vacuum. The RSE was estimated as the enthalpy difference between the ring-opened product and the total enthalpy of the isolated reactants (*c*SICH + ethylene), using their lowest-energy conformers.

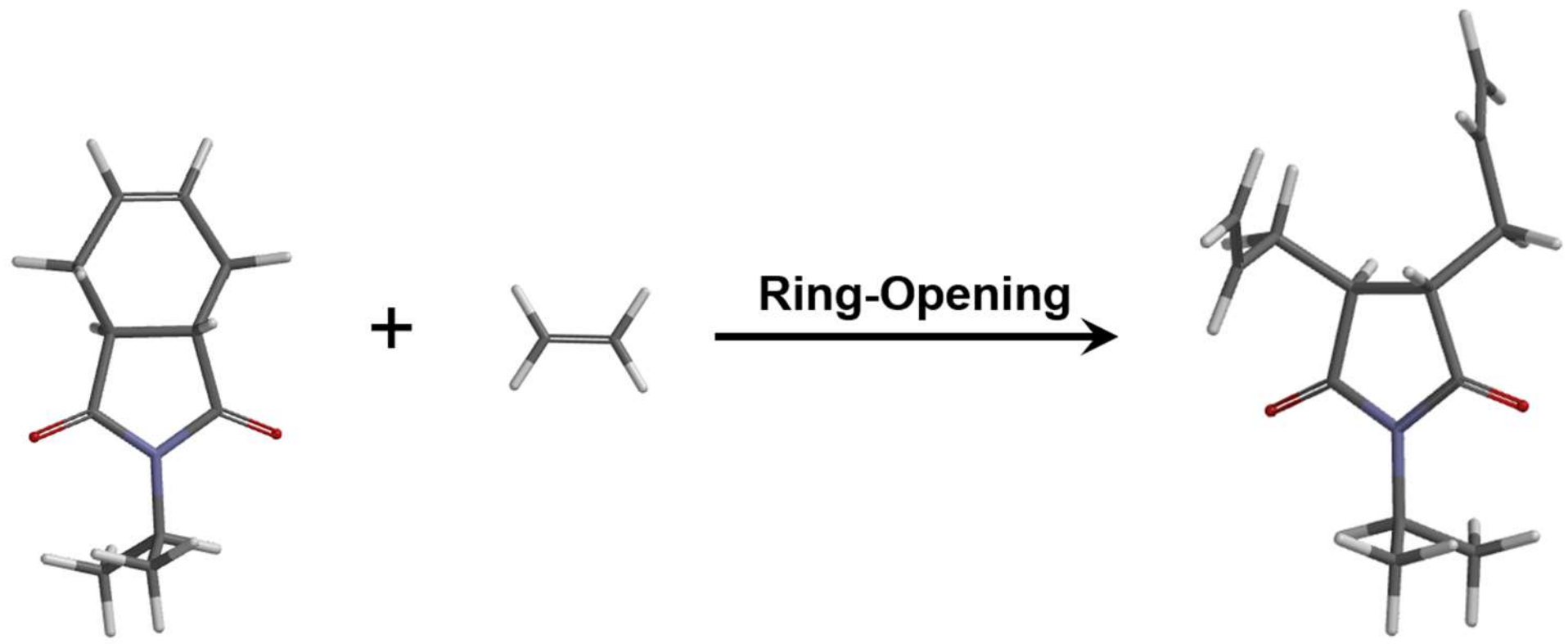

Chemical Structure	Enthalpy (Hartree)
M1	-633.168293
Ethylene	-78.5322597
Ring-Opened Product	-711.710516

RSE = -ΔH = 711.710516 - 711.700553 = 0.009963 Hartree = 6.2 kcal/mol

Figure S45. DFT calculation of ethenolysis ring strain energy (RSE) of M1. A B3LYP/6-31G* level of theory was applied for the geometry optimization and energy calculation of conformers in vacuum. The RSE was estimated as the enthalpy difference between the ring-opened product and the total enthalpy of the isolated reactants (M1 + ethylene), using their lowest-energy conformers.

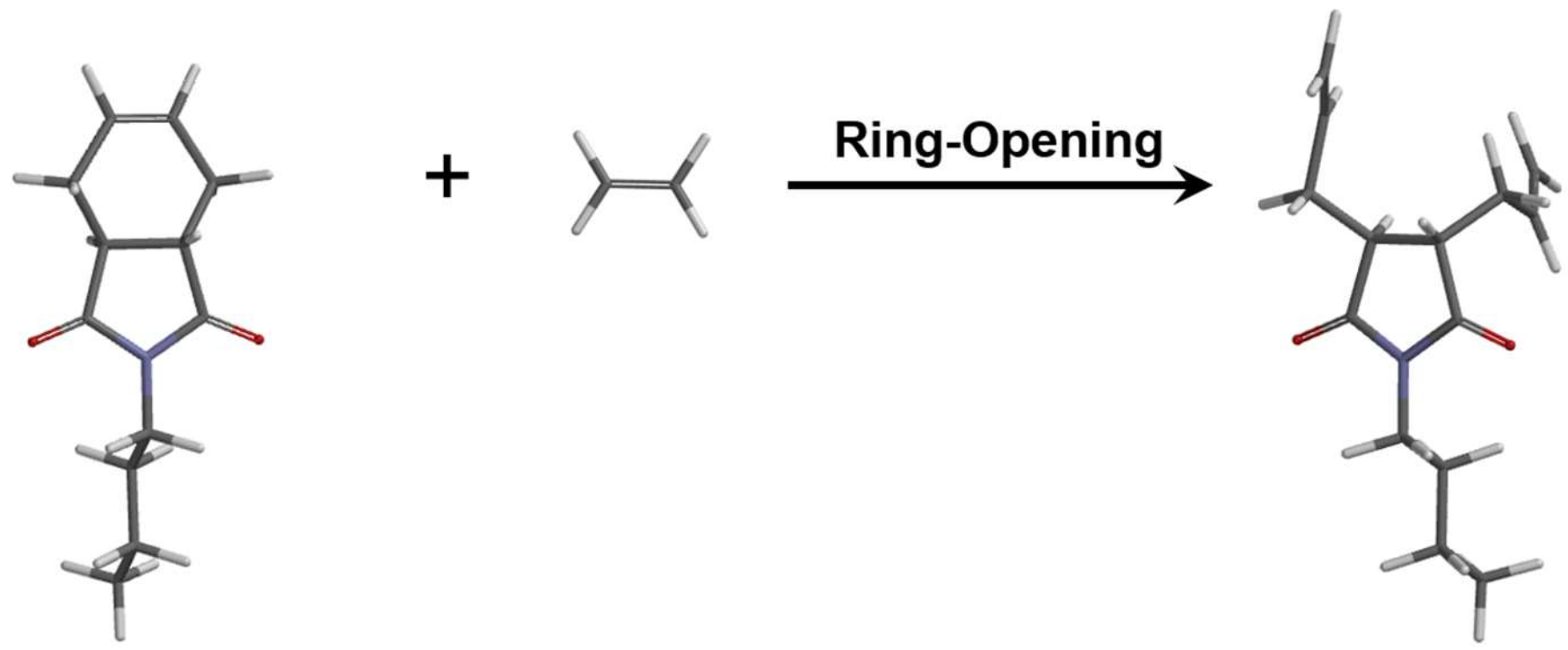

Chemical Structure	Enthalpy (Hartree)
M2	-672.451815
Ethylene	-78.5322597
Ring-Opened Product	-750.993784

RSE = -ΔH = 750.993784 - 750.9840747 = 0.009709 Hartree = 6.1 kcal/mol

Figure S46. DFT calculation of ethenolysis ring strain energy (RSE) of M2. A B3LYP/6-31G* level of theory was applied for the geometry optimization and energy calculation of conformers in vacuum. The RSE was estimated as the enthalpy difference between the ring-opened product and the total enthalpy of the isolated reactants (M2 + ethylene), using their lowest-energy conformers.

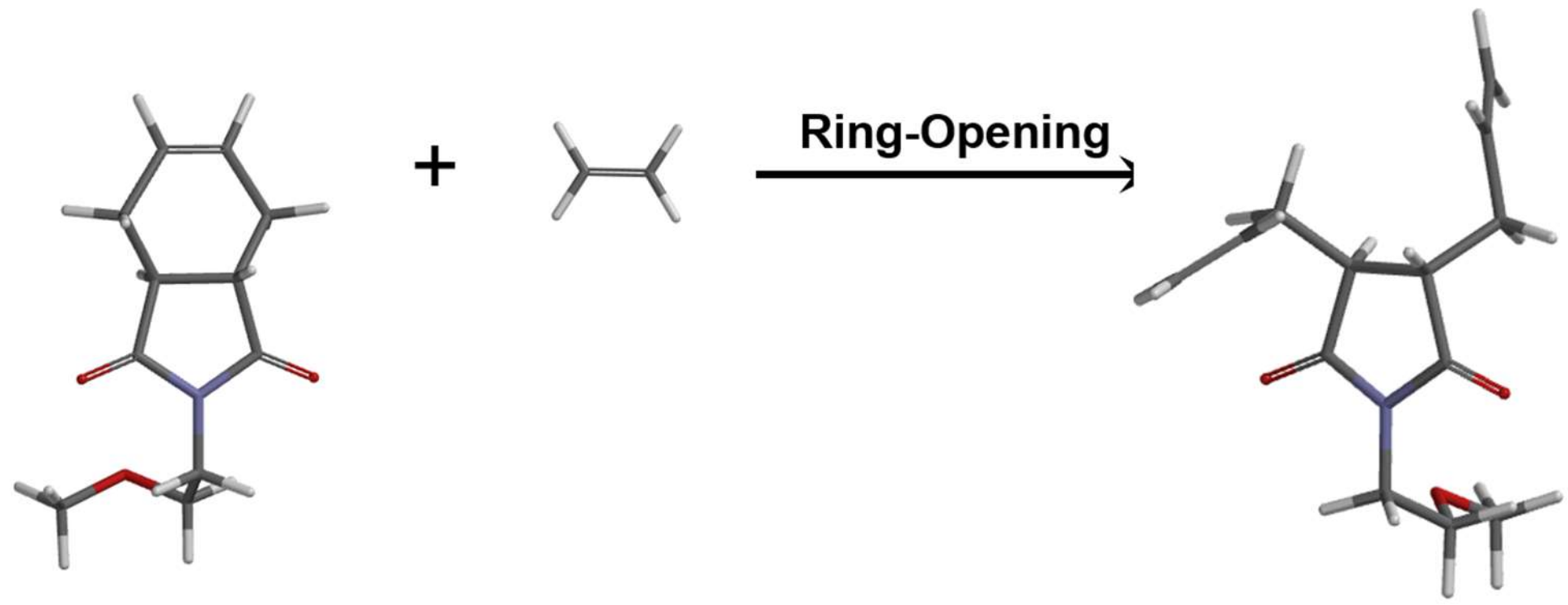

Chemical Structure	Enthalpy (Hartree)
M3	-708.360726
Ethylene	-78.5322597
Ring-Opened Product	-786.902854

RSE = -ΔH = 786.902854 - 786.8929857 = 0.009868 Hartree = 6.2 kcal/mol

Figure S47. DFT calculation of ethenolysis ring strain energy (RSE) of M3. A B3LYP/6-31G* level of theory was applied for the geometry optimization and energy calculation of conformers in vacuum. The RSE was estimated as the enthalpy difference between the ring-opened product and the total enthalpy of the isolated reactants (M3 + ethylene), using their lowest-energy conformers.

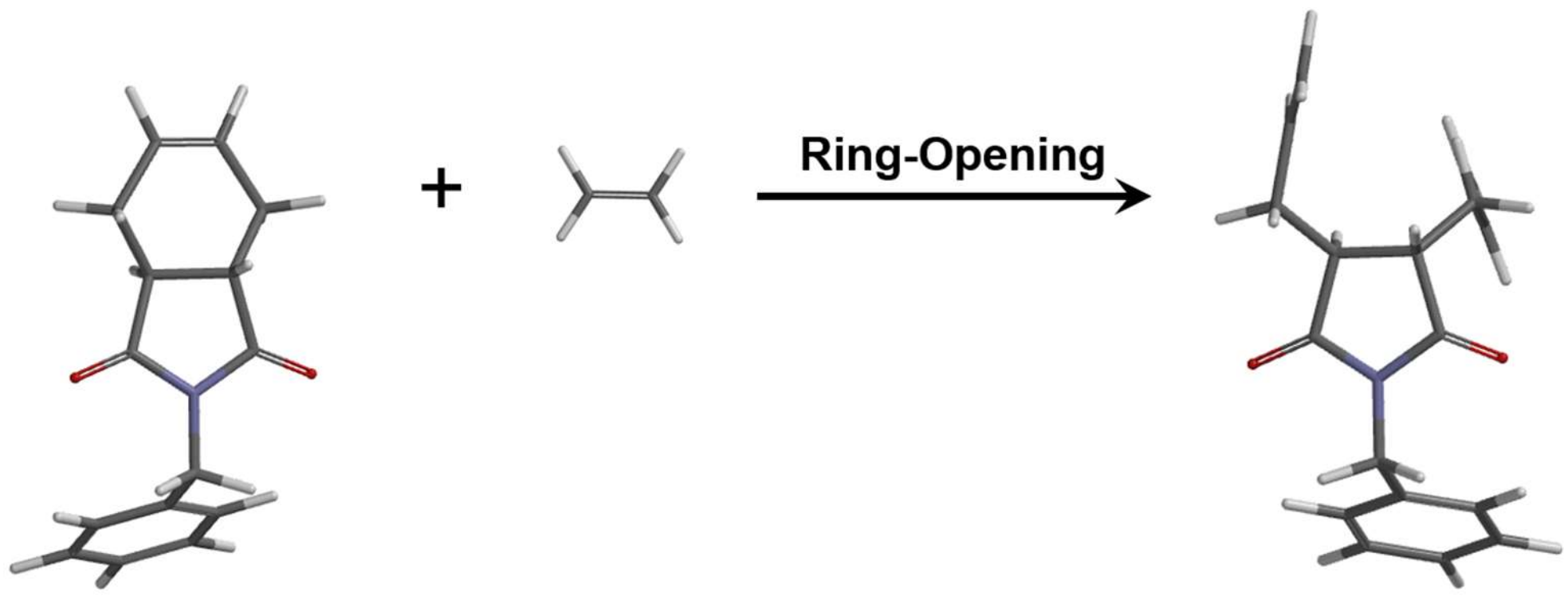

Chemical Structure	Enthalpy (Hartree)
M4	-785.563361
Ethylene	-78.5322597
Ring-Opened Product	-864.105441

RSE = -ΔH = 864.105441 - 864.095621 = 0.009820 Hartree = 6.2 kcal/mol

Figure S48. DFT calculation of ethenolysis ring strain energy (RSE) of M4. A B3LYP/6-31G* level of theory was applied for the geometry optimization and energy calculation of conformers in vacuum. The RSE was estimated as the enthalpy difference between the ring-opened product and the total enthalpy of the isolated reactants (M4 + ethylene), using their lowest-energy conformers.

6. Optimized Geometries of Monomers and Their Ring-Opened Structures from DFT Calculations

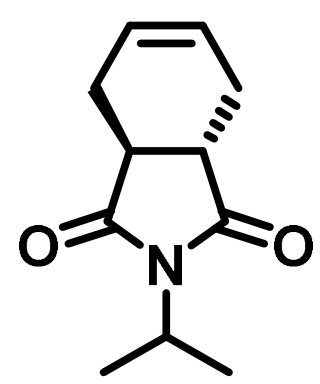

M1

C	2.111914	-1.616097	-0.199227
C	1.136508	0.704061	-0.301454
C	3.551194	0.447910	0.101711
C	2.385140	1.415364	0.203519
C	3.432210	-0.872277	-0.103720
C	1.011416	-0.694394	0.309044
C	-0.458984	-1.029603	0.099056
N	-1.129927	0.199587	0.004064
C	-0.249324	1.292821	-0.095357
O	-0.984803	-2.124874	0.070140
O	-0.583520	2.459405	-0.075121
C	-2.596776	0.369344	-0.007366
C	-3.208686	-0.236622	-1.277037
C	-3.228435	-0.184834	1.274971
H	2.151646	-2.540191	0.389860
H	1.916631	-1.927981	-1.236183
H	1.235124	0.582239	-1.392604
H	4.547219	0.879442	0.187586
H	2.252883	1.754369	1.241913
H	2.587209	2.319172	-0.384200
H	4.335258	-1.473954	-0.193654
H	1.132818	-0.592201	1.399745
H	-2.731123	1.455128	-0.028502
H	-3.063269	-1.319845	-1.301573
H	-4.283972	-0.028235	-1.303305
H	-2.757919	0.199233	-2.175272
H	-3.088289	-1.266865	1.346370
H	-4.303518	0.025335	1.274588
H	-2.791692	0.286961	2.161787

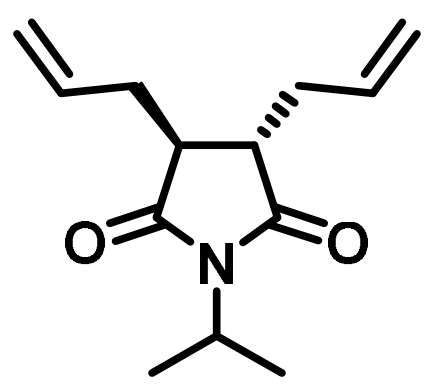

Ring-Opened M1

C	0.783818	0.596385	-0.396974
C	-0.701559	0.896221	-0.612010
N	-1.446728	0.035983	0.193505
C	-0.646215	-0.767238	1.008645
C	0.814788	-0.364035	0.806933
O	-1.167823	1.730148	-1.365055
O	-1.065718	-1.629048	1.756383
C	-2.921473	-0.054667	0.207014
C	-3.547900	1.255183	0.700664
C	-3.456242	-0.490469	-1.162318
C	1.745930	-1.594423	0.702625
C	1.606667	1.892927	-0.263533
C	3.089572	1.646034	-0.247095
C	3.914218	1.979462	0.747193
C	1.425158	-2.507598	-0.449209
C	2.240314	-2.760799	-1.474794
H	1.116792	0.059921	-1.297583
H	1.104698	0.191944	1.710100
H	-3.122727	-0.844864	0.936206
H	-3.337741	2.075555	0.009522
H	-4.634093	1.135394	0.778746
H	-3.165473	1.522324	1.691783
H	-3.242935	0.262596	-1.925546
H	-4.541454	-0.628435	-1.104869
H	-3.009890	-1.442065	-1.470437
H	2.781100	-1.242928	0.632211
H	1.648750	-2.151703	1.643546
H	1.307148	2.430115	0.646299
H	1.339799	2.535591	-1.113276
H	3.497531	1.154503	-1.132504
H	3.554295	2.474977	1.646966
H	4.981753	1.783292	0.695796
H	0.447836	-2.990893	-0.413382
H	1.959130	-3.438485	-2.276590
H	3.228447	-2.309420	-1.544988

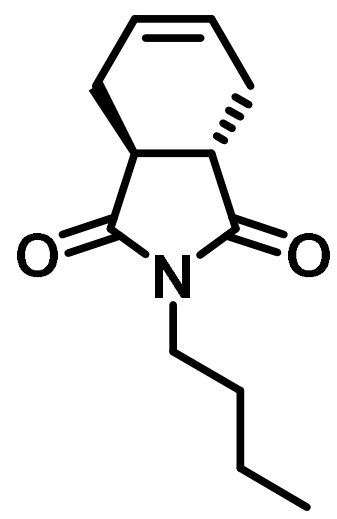

M2

C	2.686193	-1.567266	-0.412262
C	1.581308	0.695700	-0.281458
C	4.036064	0.576877	-0.442453
C	2.871141	1.479095	-0.076045
C	3.949082	-0.750035	-0.618177
C	1.682130	-0.703344	0.339197
C	0.226591	-1.120685	0.487611
N	-0.510471	0.071011	0.552364
C	0.249331	1.210397	0.244746
O	-0.245541	-2.233183	0.601911
O	-0.144477	2.354062	0.345198
C	-1.927369	0.119964	0.903174
C	-2.849852	-0.017785	-0.313140
C	-4.331504	0.020604	0.080282
C	-5.270376	-0.109941	-1.122873
H	2.911415	-2.482417	0.148857
H	2.276430	-1.898649	-1.378104
H	1.433099	0.568374	-1.366280
H	4.999441	1.063887	-0.585310
H	2.960577	1.823593	0.965049
H	2.882433	2.384974	-0.694254
H	4.840964	-1.301109	-0.912215
H	2.050055	-0.580840	1.371173
H	-2.093423	1.076649	1.407615
H	-2.111570	-0.691482	1.613678
H	-2.632018	0.793078	-1.021095
H	-2.624603	-0.962918	-0.824012
H	-4.542314	0.960462	0.610057
H	-4.537159	-0.787885	0.795888
H	-5.107906	0.703092	-1.840791
H	-6.321042	-0.077327	-0.813696
H	-5.107378	-1.057073	-1.651607

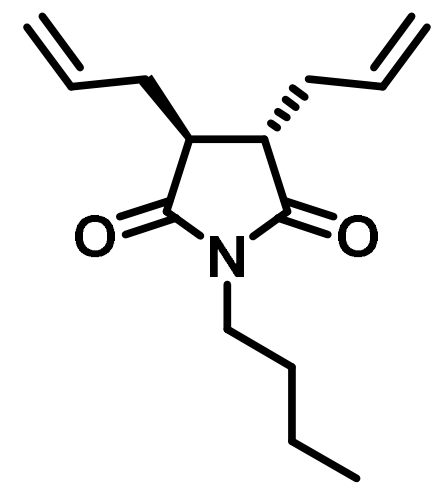

Ring-Opened M2

C	-1.344807	-0.370840	-0.817496
C	0.123071	-0.354054	-1.250944
N	0.785445	0.625784	-0.516455
C	-0.025382	1.244606	0.433536
C	-1.401106	0.577334	0.397754
C	2.198966	0.947814	-0.700152
C	3.116688	0.081890	0.170868
C	4.596780	0.439051	-0.010561
C	5.524268	-0.431646	0.843055
O	0.331518	2.154822	1.154920
O	0.648388	-1.054795	-2.094368
C	-2.529985	1.628471	0.393379
C	-1.862288	-1.813011	-0.603939
C	-3.894715	1.019064	0.554905
C	-1.135959	-2.571347	0.473931
C	-1.691104	-3.013672	1.603854
C	-4.884363	1.096493	-0.336337
H	-1.918786	0.061821	-1.648865
H	-1.475603	-0.014551	1.321305
H	2.424714	0.800983	-1.760117
H	2.316743	2.006391	-0.451862
H	2.954023	-0.973375	-0.084243
H	2.830839	0.209495	1.223219
H	4.870186	0.336019	-1.070142
H	4.748523	1.497169	0.244940
H	5.429089	-1.491045	0.575516
H	6.573690	-0.146539	0.708889
H	5.285857	-0.336033	1.909333
H	-2.325963	2.326088	1.216448
H	-2.487821	2.212374	-0.535454
H	-1.745071	-2.338999	-1.560228
H	-2.933871	-1.769217	-0.379739
H	-4.060296	0.471018	1.484559
H	-0.077988	-2.764279	0.290189
H	-2.745439	-2.854708	1.823018
H	-1.119236	-3.564211	2.346162
H	-5.852549	0.636826	-0.157024
H	-4.768804	1.633160	-1.276258

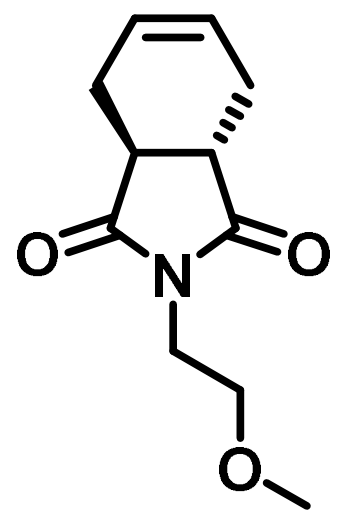

M3

C	2.292703	-1.680881	-0.280830
C	1.528234	0.721199	-0.278161
C	3.709232	0.167629	0.720480
C	2.621778	1.225107	0.654481
C	3.574168	-1.093173	0.283261
C	1.149708	-0.728919	0.046278
C	-0.220521	-0.880169	-0.595820
N	-0.751717	0.416706	-0.675617
C	0.177484	1.416431	-0.356176
O	-0.794649	-1.890673	-0.947247
O	-0.090542	2.594971	-0.238846
C	-2.152630	0.692591	-0.958782
C	-2.969002	0.875041	0.330442
O	-2.883056	-0.217552	1.220923
C	-3.629239	-1.359147	0.826622
H	2.110646	-2.673887	0.147657
H	2.380869	-1.832630	-1.367090
H	1.924902	0.740196	-1.306417
H	4.664623	0.480000	1.139001
H	2.219046	1.431278	1.657353
H	3.035929	2.174986	0.295113
H	4.427892	-1.765961	0.347121
H	0.959292	-0.785281	1.130283
H	-2.525739	-0.140733	-1.558961
H	-2.209901	1.610557	-1.551902
H	-2.579399	1.740121	0.874768
H	-4.017496	1.082696	0.056692
H	-4.681375	-1.098542	0.629806
H	-3.590465	-2.060035	1.664686
H	-3.202315	-1.847928	-0.058203

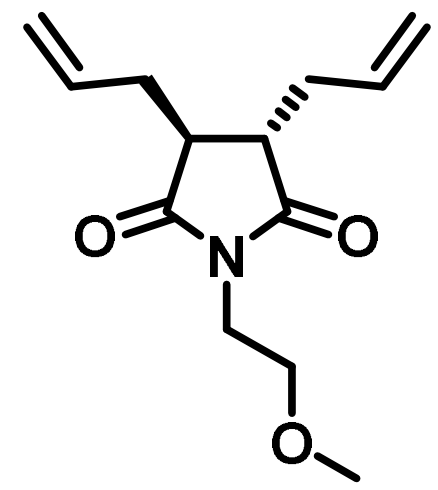

Ring-Opened M3

C	-0.996242	-0.766897	-0.679702
C	0.257225	0.000896	-1.098182
N	0.474270	1.022112	-0.176792
C	-0.463748	1.043461	0.853233
C	-1.402806	-0.148813	0.673590
O	-0.490140	1.879166	1.736140
O	0.952091	-0.211574	-2.073360
C	1.623865	1.913761	-0.226332
C	2.740659	1.445712	0.720497
O	3.174974	0.121698	0.487637
C	4.004344	-0.033445	-0.655427
C	-2.889735	0.240475	0.840608
C	-0.736037	-2.292519	-0.688181
C	-3.383414	1.239868	-0.170236
C	0.272365	-2.741043	0.336333
C	0.042666	-3.659717	1.276259
C	-4.332277	1.002963	-1.077696
H	-1.758911	-0.544769	-1.437692
H	-1.148379	-0.846979	1.482835
H	1.959918	1.940246	-1.265054
H	1.295561	2.914338	0.071155
H	2.365893	1.464137	1.748381
H	3.582067	2.155428	0.649080
H	4.847722	0.674655	-0.631315
H	4.398002	-1.052484	-0.617356
H	3.445478	0.092701	-1.590645
H	-3.498528	-0.670833	0.795342
H	-3.001580	0.656795	1.850209
H	-0.377470	-2.546112	-1.694748
H	-1.684454	-2.822609	-0.537882
H	-2.916283	2.225057	-0.131718
H	1.252149	-2.262585	0.288817
H	-0.919257	-4.162148	1.363039
H	0.809914	-3.954693	1.987063
H	-4.656642	1.764690	-1.781690
H	-4.831676	0.037845	-1.142200

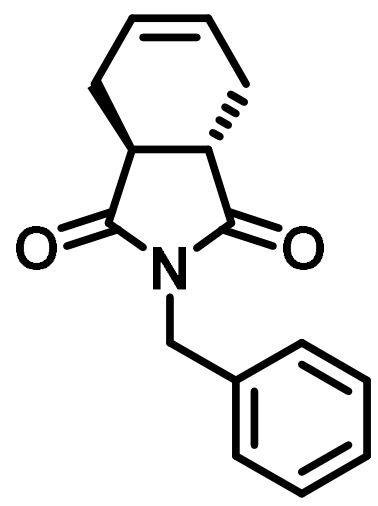

M4

C	3.172451	-1.525364	0.326793
C	2.157837	0.686610	-0.331839
C	4.164698	0.675810	1.096557
C	2.999378	1.525806	0.620665
C	4.253290	-0.653645	0.941348
C	1.891389	-0.705989	0.251542
C	0.682467	-1.186520	-0.536449
N	0.033093	-0.026096	-0.984989
C	0.774372	1.144153	-0.772143
O	0.284202	-2.318124	-0.721057
O	0.366118	2.270400	-0.972102
C	-1.310214	-0.036393	-1.573459
C	-2.410953	-0.004312	-0.530510
C	-2.992928	-1.194360	-0.077409
C	-3.999064	-1.164788	0.889547
C	-4.430646	0.054991	1.413506
C	-3.849950	1.245386	0.969848
C	-2.844535	1.216329	0.003178
H	3.023841	-2.429665	0.929521
H	3.476468	-1.874734	-0.671208
H	2.731131	0.553655	-1.263460
H	4.983763	1.204941	1.581052
H	2.398237	1.870799	1.475120
H	3.367062	2.432543	0.125207
H	5.148147	-1.166030	1.290824
H	1.517849	-0.566598	1.279330
H	-1.380601	-0.942028	-2.180872
H	-1.370003	0.837489	-2.226668
H	-2.647329	-2.143578	-0.478419
H	-4.445671	-2.094524	1.232233
H	-5.216681	0.078299	2.163893
H	-4.182359	2.197841	1.374420
H	-2.383602	2.139747	-0.337822

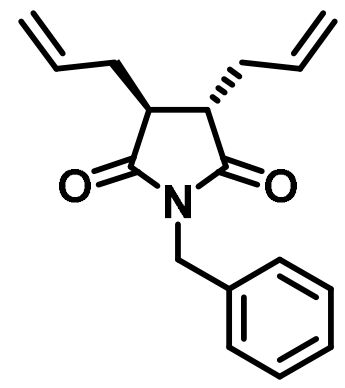

Ring-Opened M4

C	1.800129	0.549691	-0.378072
C	0.529477	1.381637	-0.206441
N	-0.225453	0.817209	0.820500
C	0.389674	-0.277120	1.420682
C	1.776962	-0.448570	0.797844
O	0.203365	2.367551	-0.837954
O	-0.109013	-0.951451	2.301430
C	-1.554742	1.306713	1.202579
C	-2.675526	0.647672	0.422718
C	-3.140161	1.217458	-0.769182
C	-4.167990	0.606713	-1.489209
C	-4.738216	-0.580425	-1.025645
C	-4.274704	-1.156762	0.158821
C	-3.248203	-0.546131	0.879982
C	2.083563	-1.926988	0.461751
C	3.046611	1.453290	-0.482320
C	1.169309	-2.524290	-0.574224
C	4.301121	0.685349	-0.792803
C	5.386528	0.646715	-0.017809
C	1.562054	-2.977571	-1.766168
H	1.680075	0.001006	-1.322829
H	2.498004	-0.132004	1.564449
H	-1.546920	2.385519	1.031389
H	-1.661601	1.113719	2.272672
H	-2.686503	2.135830	-1.133032
H	-4.524240	1.059857	-2.410649
H	-5.541321	-1.053928	-1.584458
H	-4.715057	-2.080989	0.524044
H	-2.879819	-0.997249	1.797574
H	3.126001	-1.996523	0.131442
H	1.994899	-2.494095	1.397644
H	3.174962	2.014390	0.452714
H	2.846598	2.191271	-1.270229
H	0.113849	-2.581852	-0.304713
H	4.296456	0.125853	-1.730023
H	5.435619	1.192458	0.922455
H	6.270519	0.079589	-0.296578
H	0.860162	-3.407360	-2.475880
H	2.606361	-2.949047	-2.072330

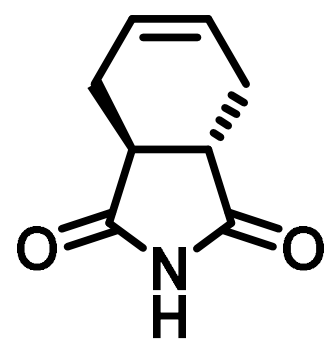

***t*SICH**

C	-0.216706	-1.519137	-1.316866
C	-0.299830	0.706522	-0.136924
C	0.109430	0.661597	-2.565010
C	0.216706	1.519137	-1.316866
C	-0.109430	-0.661597	-2.565010
C	0.299830	-0.706522	-0.136924
C	0.096108	-1.177471	1.298050
N	0.000000	0.000000	2.054283
C	-0.096108	1.177471	1.298050
O	0.069885	-2.302485	1.744649
O	-0.069885	2.302485	1.744649
H	0.360447	-2.443781	-1.439489
H	-1.258080	-1.833587	-1.151569
H	-1.391506	0.602455	-0.247650
H	0.203434	1.179853	-3.517918
H	1.258080	1.833587	-1.151569
H	-0.360447	2.443781	-1.439489
H	-0.203434	-1.179853	-3.517918
H	1.391506	-0.602455	-0.247650
H	0.000000	0.000000	3.067878

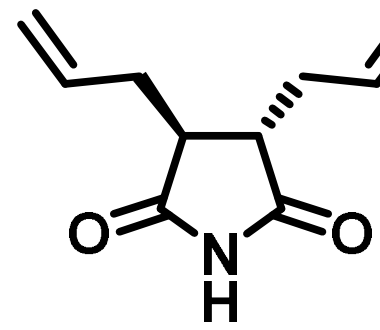

Ring-Opened *t*SICH

C	0.402290	-0.541506	0.410063
C	-0.535757	-1.719407	0.691523
N	-1.683042	-1.525237	-0.073983
C	-1.651642	-0.405170	-0.899630
C	-0.264393	0.229554	-0.751431
O	-2.566445	-0.029935	-1.601259
O	-0.345793	-2.656022	1.436840
C	-0.345629	1.767382	-0.608705
C	1.843631	-1.031798	0.163053
C	-1.107277	2.231520	0.603454
C	2.835383	0.091385	0.039175
C	3.602738	0.323217	-1.027332
C	-0.583267	2.924828	1.615909
H	0.388197	0.080749	1.316375
H	-2.481102	-2.148595	-0.028687
H	0.271383	0.015321	-1.686715
H	0.674180	2.167214	-0.591932
H	-0.835598	2.148238	-1.514146
H	1.869912	-1.653891	-0.741387
H	2.112449	-1.684127	1.004648
H	-2.166825	1.973368	0.627975
H	2.915846	0.752292	0.904525
H	3.560736	-0.310625	-1.911077
H	4.311542	1.146429	-1.054871
H	-1.183620	3.242471	2.464130
H	0.465640	3.215444	1.627921

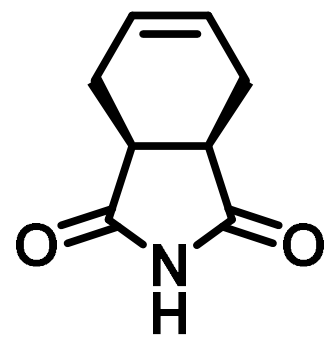

***c*SICH**

C	-1.252712	-0.262764	1.421754
C	-0.115987	0.570244	-0.775080
C	-2.547316	-0.075835	-0.668220
C	-1.252712	-0.262764	-1.421754
C	-2.547316	-0.075835	0.668220
C	-0.115987	0.570244	0.775080
C	1.265894	0.052491	1.176488
N	1.969829	-0.198299	0.000000
C	1.265894	0.052491	-1.176488
O	1.692097	-0.130993	2.296035
O	1.692097	-0.130993	-2.296035
H	-0.971708	-1.326949	1.412163
H	-1.347015	0.012076	2.475744
H	-0.199055	1.596118	-1.150339
H	-3.470529	0.037475	-1.232002
H	-0.971708	-1.326949	-1.412163
H	-1.347015	0.012076	-2.475744
H	-3.470529	0.037475	1.232002
H	-0.199055	1.596118	1.150339
H	2.915714	-0.563081	0.000000

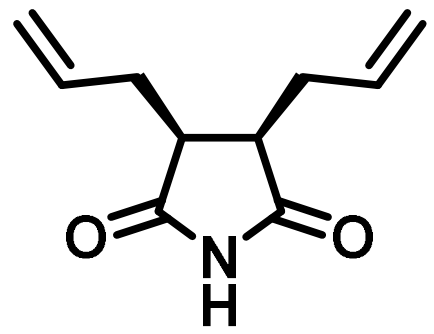

Ring-Opened *c*SICH

C	0.209431	-0.465037	-0.526946
C	-0.426685	-1.850400	-0.355916
N	-1.721895	-1.652973	0.119184
C	-2.124419	-0.323471	0.184061
C	-1.002045	0.509453	-0.446124
O	-3.180862	0.068335	0.630200
O	0.078182	-2.937992	-0.538692
C	-0.845602	1.875369	0.247357
C	1.304218	-0.254955	0.556175
C	2.513389	-1.133082	0.363017
C	-0.018299	2.856974	-0.537962
C	1.003251	3.565532	-0.054543
C	3.754709	-0.666062	0.223968
H	0.693429	-0.424151	-1.507322
H	-2.323037	-2.421040	0.394778
H	-1.346190	0.701510	-1.473958
H	-1.863721	2.269463	0.373626
H	-0.435890	1.754341	1.256758
H	0.862064	-0.438590	1.547373
H	1.610411	0.795149	0.530676
H	2.331636	-2.205470	0.330073
H	-0.312965	2.997099	-1.580094
H	1.545389	4.279959	-0.668090
H	1.329458	3.466039	0.979136
H	4.602739	-1.332518	0.091157
H	3.973699	0.400339	0.241018

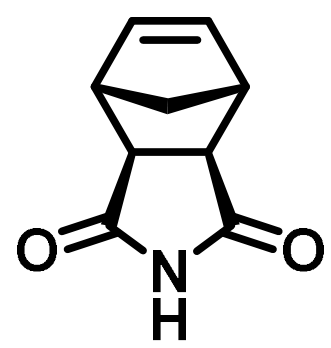

Exo-NBE Imide

C	-1.203080	-0.326141	1.130541
C	-0.032979	0.669113	-0.776720
C	-2.477404	0.371496	-0.670241
C	-1.203080	-0.326141	-1.130541
C	-2.477404	0.371496	0.670241
C	-0.032979	0.669113	0.776720
C	-1.045771	-1.371165	0.000000
C	1.345150	0.148454	1.178372
N	2.050786	-0.102230	-0.000000
C	1.345150	0.148454	-1.178372
O	1.773387	-0.036760	2.296920
O	1.773387	-0.036760	-2.296920
H	-1.175013	-0.680959	2.162085
H	-0.187434	1.648084	-1.237823
H	-3.184504	0.862652	-1.330934
H	-1.175013	-0.680959	-2.162085
H	-3.184504	0.862652	1.330934
H	-0.187434	1.648084	1.237823
H	-1.851392	-2.110456	0.000000
H	-0.081627	-1.892471	0.000000
H	2.991599	-0.480930	-0.000000

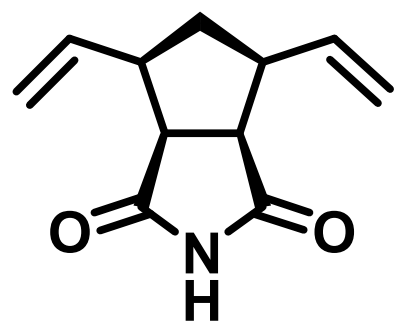

Ring-Opened exo-NBE Imide

C	-0.168943	-0.909948	-1.226733
C	0.672632	0.328893	-0.774268
C	0.672632	0.328893	0.774268
C	-0.168943	-0.909948	1.226733
C	-0.069530	-1.844399	0.000000
C	0.055130	1.664948	1.180597
N	-0.264970	2.336771	0.000000
C	0.055130	1.664948	-1.180597
O	-0.152488	2.086563	-2.296902
O	-0.152488	2.086563	2.296902
C	0.296302	-1.528274	-2.513312
C	0.296302	-1.528274	2.513312
C	-0.451311	-1.684520	3.605435
C	-0.451311	-1.684520	-3.605435
H	-1.216105	-0.601544	-1.345251
H	1.680926	0.270643	-1.197577
H	1.680926	0.270643	1.197577
H	-1.216105	-0.601544	1.345251
H	-0.839263	-2.623238	0.000000
H	0.906140	-2.351967	0.000000
H	-0.712023	3.246634	0.000000
H	1.331680	-1.876637	-2.518330
H	1.331680	-1.876637	2.518330
H	-1.483488	-1.343262	3.646089
H	-0.057413	-2.149514	4.504854
H	-1.483488	-1.343262	-3.646089
H	-0.057413	-2.149514	-4.504854

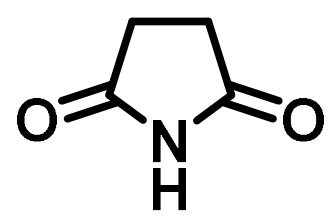

Succinimide

C	-0.000026	0.214563	1.178373
N	-0.000724	0.958627	0.000000
C	-0.000026	0.214563	-1.178373
C	-0.000059	-1.259675	-0.769925
C	-0.000059	-1.259675	0.769925
O	0.000478	0.677158	2.296736
O	0.000478	0.677158	-2.296736
H	-0.000943	1.971955	0.000000
H	0.879321	-1.743982	-1.205670
H	-0.879628	-1.743778	-1.205576
H	0.879321	-1.743982	1.205670
H	-0.879628	-1.743778	1.205576

References

1. Goodridge, R. J.; Hambley, T. W.; Ridley, D. D., Preparations and Crystal Structures of the 2-Oxides of Some Octahydro-3,2,1-benzoxathiazines and Octahydro-2 H-3,1,2-benzoxazaphosphorine. *Aust. J. Chem.* **1986,** *39*, 591-604.
2. Bloomfield, J. J.; Lee, S. L., Control of lithium aluminum hydride reduction of cyclic dicarboxylic acid anhydrides to produce .gamma.-lactones or diols. *J. Org. Chem.* **1967,** *32* (12), 3919-3924.
3. Stellmach, K. A.; Paul, M. K.; Su, Y.-L.; Ramprasad, R.; Engler, A. C.; Gutekunst, W. R., Improving the Accuracy of Ceiling Temperature Measurements: Best Practices and Common Pitfalls. *Macromolecules* **2025,** *58* (8), 3729-3741.
4. Ibrahim, T.; Brown, D.; Sun, H., Entropy-Driven Design of Depolymerizable Polyolefins from Strained Bridged Bicyclic Monomers. *J. Am. Chem. Soc.* **2026,** *148* (15), 16317-16325.
